# Green Anaconda Optimization: A New Bio-Inspired Metaheuristic Algorithm for Solving Optimization Problems

**DOI:** 10.3390/biomimetics8010121

**Published:** 2023-03-14

**Authors:** Mohammad Dehghani, Pavel Trojovský, Om Parkash Malik

**Affiliations:** 1 Department of Mathematics, Faculty of Science, University of Hradec Králové, 500 03 Hradec Králové, Czech Republic; 2 Department of Electrical and Computer Engineering, University of Calgary, Calgary, AB T2N 1N4, Canada

**Keywords:** optimization, bio-inspired, metaheuristic, green anaconda, exploration, exploitation

## Abstract

A new metaheuristic algorithm called green anaconda optimization (GAO) which imitates the natural behavior of green anacondas has been designed. The fundamental inspiration for GAO is the mechanism of recognizing the position of the female species by the male species during the mating season and the hunting strategy of green anacondas. GAO’s mathematical modeling is presented based on the simulation of these two strategies of green anacondas in two phases of exploration and exploitation. The effectiveness of the proposed GAO approach in solving optimization problems is evaluated on twenty-nine objective functions from the CEC 2017 test suite and the CEC 2019 test suite. The efficiency of GAO in providing solutions for optimization problems is compared with the performance of twelve well-known metaheuristic algorithms. The simulation results show that the proposed GAO approach has a high capability in exploration, exploitation, and creating a balance between them and performs better compared to competitor algorithms. In addition, the implementation of GAO on twenty-one optimization problems from the CEC 2011 test suite indicates the effective capability of the proposed approach in handling real-world applications.

## 1. Introduction

Optimization has long been discussed in various branches of science in order to achieve the best solution in multi-solution problems [1]. Optimization is employed in addressing many optimization challenges in technology, engineering, and real-life applications [2]. An optimization problem is modeled from a mathematical point of view using decision variables that must be quantified, problem constraints that must be justified, and the objective function that must be optimized [3]. Problem-solving approaches in the field of optimization are classified into two classes: deterministic and stochastic techniques [4]. Deterministic techniques fall into two categories: gradient-based and non-gradient-based. These techniques are effective in solving linear, convex, simple optimization problems and simple real-world applications [5]. The need for first and second order derivative information and dependence on initial starting points are among the disadvantages of these techniques. This is so because, with the advancement of science and technology, scientists are faced with more complex and emerging optimization problems that are non-linear, non-convex, high-dimensional, and non-differentiable in nature. These characteristics lead to the inability of deterministic techniques to deal with such optimization problems and finally become stuck in local optima solutions [6]. The difficulties of deterministic techniques led scholars to explore new techniques called stochastic approaches to handle complex optimization tasks. Effective performance in nonlinear, discontinuous, complex, high-dimensional, NP-hard, non-convex optimization problems and nonlinear, discrete, and unknown search spaces are among the advantages that have led to the popularity of metaheuristic algorithms [7].

The operation of searching for a solution in metaheuristic algorithms starts by randomly generating a certain number of initial candidate solutions. In each iteration, under the influence of algorithm steps, candidate solutions are improved. After completing the iterations of the algorithm, the best candidate solution is identified among the solutions and presented as the solution to the given problem [8].

Metaheuristic algorithms should search the problem-solving space at both global and local levels well and carefully. The main goal of global search with the concept of exploration is the ability of the metaheuristic algorithm to identify the main optimal region and avoid getting stuck in local optima. The main goal of local search with the concept of exploitation is the ability of the metaheuristic algorithm to converge towards possible better solutions in the vicinity of the solutions discovered in the promising regions of the problem-solving space. Considering that exploration and exploitation pursue opposite goals, balancing them during the search process is the key to the success of metaheuristic algorithms [9]. The search process in metaheuristic algorithms has a random nature, which makes the solutions resulting from these approaches not guaranteed to be global optimal. At the same time, considering that these solutions are close to the global optimum, they are accepted as quasi-optimal solutions. Therefore, in the comparison of several metaheuristic algorithms, the algorithm that provides a quasi-optimal solution closer to the global optimum has superior performance. Achieving better quasi-optimal solutions and solutions closer to the global optimum to more effectively solve optimization problems is the motivation source of scientists in designing numerous metaheuristic algorithms [10].

The main research question is that according to the introduction of numerous metaheuristic algorithms so far, is there still a need to design a new metaheuristic algorithm or not? In response to this question, the No Free Lunch (NFL) [11] theorem explains that the successful performance of a metaheuristic algorithm in a set of optimization problems does not guarantee the same performance of that algorithm in all other optimization problems. In fact, according to the NFL theorem, there is no specific metaheuristic algorithm that is the best optimizer for all optimization applications. This means that there is no preconceived notion about whether the implementation of a metaheuristic algorithm on an optimization problem will be successful or not. The NFL theorem is the main source of the motivation for researchers to search for and provide better solutions for optimization problems by designing newer algorithms.

The novelty and innovation of this paper are in the introduction of a new metaheuristic algorithm called green anaconda optimization (GAO), which is used in dealing with optimization problems and providing solutions for them. The key contributions of this paper are given below:

- GAO is designed based on mimicking the behavior of green anacondas in the wild.
- The fundamental inspiration for GAO is the green anaconda’s tracking mechanism during the mating season and the hunting strategy they have when attacking prey.
- The mathematical model of GAO is presented in two phases with the aim of forming exploration and exploitation in the search process.
- GAO’s performance on optimization tasks is tested on twenty-nine benchmark functions from the CEC 2017 test suite and CEC 2019 test suite.
- GAO’s ability to handle real-world applications is evaluated on twenty-one optimization problems from the CEC 2011 test suite.
- The results obtained from GAO are compared with the performance of twelve well-known metaheuristic algorithms.

The structure of the paper is as follows: the literature review is presented in Section 2. Then, the proposed green anaconda optimization approach is introduced and modeled in Section 3. The simulation studies and results are presented in Section 4. The effectiveness of GAO in solving real-world applications is investigated in Section 5. Conclusions and suggestions for future research are provided in Section 6.

## 2. Literature Review

Metaheuristic algorithms in design are inspired by various natural phenomena, animal behavior in nature, laws of physics, rules of games, biological sciences, human interactions, and any other phenomenon that has an evolutionary process. According to this, in terms of the idea inspired in the design, metaheuristic algorithms are placed in five classes: swarm-based, evolutionary-based, physics-based, human-based, and game-based approaches.

Swarm-based metaheuristic algorithms have been developed based on the simulation of various swarming phenomena in nature, including the behaviors and strategies of animals, insects, birds, aquatic organisms and other living organisms. Among the most famous swarm-based algorithms can be mentioned ant colony optimization (ACO) [12], artificial bee colony (ABC) [13], and particle swarm optimization (PSO) [14]. ACO is inspired by the ability of ants to identify the optimal route between nests and food sources. ABC is developed based on the modeling of honey bee colony activities in obtaining food resources. PSO is designed based on the simulation of flocks of fish and birds searching for food in the environment. Food provision is a basic activity among living organisms in nature, which is obtained through foraging, eating carrion, and hunting. This natural behavior has been the source of inspiration for the design of numerous algorithms, including: grey wolf optimizer (GWO) [15], the orca predation algorithm (OPA) [16], the African vultures optimization algorithm (AVOA) [17], the marine predator algorithm (MPA) [18], white shark optimizer (WSO) [19], the reptile search algorithm (RSA) [20], golden jackal optimization (GJO) [21], the whale optimization algorithm (WOA) [22], the honey badger algorithm (HBA) [23], and the tunicate swarm algorithm (TSA) [24].

Evolutionary-based metaheuristic algorithms are designed based on modeling the concepts of biological, genetic sciences, and natural selection. Genetic algorithm (GA) [25] and differential evolution (DE) [26] are among the most famous evolutionary algorithms whose main idea in their design is to simulate the reproduction process, the concept of survival of the fittest, Darwin’s theory of evolution, random selection, mutation, and crossover operators.

Physics-based metaheuristic algorithms are inspired by the phenomena, laws, concepts, and forces of physics. Simulated annealing (SA) [27] is one of the most famous approaches of this class of metaheuristic algorithms, which is inspired by the metal annealing phenomenon. In this physical process, the metal is first melted and then slowly cooled to achieve the ideal crystal. Physical forces have been a source of inspiration in designing algorithms such as the gravitational search algorithm (GSA) [28] inspired vy gravitational force, the spring search algorithm (SSA) [29] inspired by spring force, and the momentum search algorithm (MSA) [30] inspired by momentum force. The physical water cycle is employed in the design of the water cycle algorithm (WCA) [31]. Some of the other physics-based algorithms are: the Archimedes optimization algorithm (AOA) [32], Henry gas optimization (HGO) [33], the equilibrium optimizer (EO) [34], the Lichtenberg algorithm (LA) [35], nuclear reaction optimization (NRO) [36], electro-magnetism optimization (EMO) [37], the black hole algorithm (BHA) [38], the multi-verse optimizer (MVO) [39], and thermal exchange optimization (TEO) [40].

Human-based metaheuristic algorithms are formed based on the simulation of human behavior, activities, and interactions. Teaching–learning-based optimization (TLBO) is one of the most widely used human-based approaches, which is designed based on imitating the classroom learning environment and interactions between students and teachers [41]. Human interactions in the field of therapy between doctors and patients are employed in the design of doctor and patient optimization (DPO) [42]. The development of society and the improvement of people’s living standards under the influence of the leader of that society has been the origin of following optimization algorithm (FOA) design [43]. The strategy of military troops during ancient wars has been the main inspiration in the design of war strategy optimization (WSO) [44]. Some of the other human-based algorithms are: the teamwork optimization algorithm (TOA) [45], Ali Baba and the forty thieves (AFT) [46], driving-training-based optimization (DTBO) [6], the gaining–sharing-knowledge-based algorithm (GSK) [47], and the Coronavirus herd immunity optimizer (CHIO) [48].

Game-based metaheuristic algorithms have been introduced based on the modeling of game rules, players’ strategies, referees, and other influential persons in games. Players trying to find a hidden object in the game space has been the main idea in the design of the hide object game optimizer (HOGO) [49]. The strategy of players in changing the direction of movement based on the direction determined by the referee in the orientation game is employed in the design of the orientation search algorithm (OSA) [50]. The simulation of the volleyball league and the behavior of the players and coaches during the match are used in the design of the volleyball premier league (VPL) [51]. Some of the other game-based algorithms are: football-game-based optimization (FGBO) [52], the archery algorithm (AA) [7], the dice game optimizer (DGO) [53], ring-toss-game-based optimization (RTGBO) [54], and the puzzle optimization algorithm (POA) [55].

Based on the best knowledge obtained from the literature review, so far, no metaheuristic model has been designed based on simulating the natural behavior of green anacondas. Meanwhile, the strategy of moving male species towards female species in the mating season and the hunting strategy of this animal is an intelligent process that has special potential for designing a meta-heuristic algorithm. Therefore, in order to address this research gap, a new swarm-based metaheuristic algorithm is designed based on mimicking the natural behavior of green anacondas. It is described in the next section.

## 3. Green Anaconda Optimization

In this section, the inspiration source and theory of the proposed green anaconda optimization (GAO) approach for use in optimization tasks is explained and its mathematical modeling is presented.

### 3.1. Inspiration of GAO

**Figure 1:**
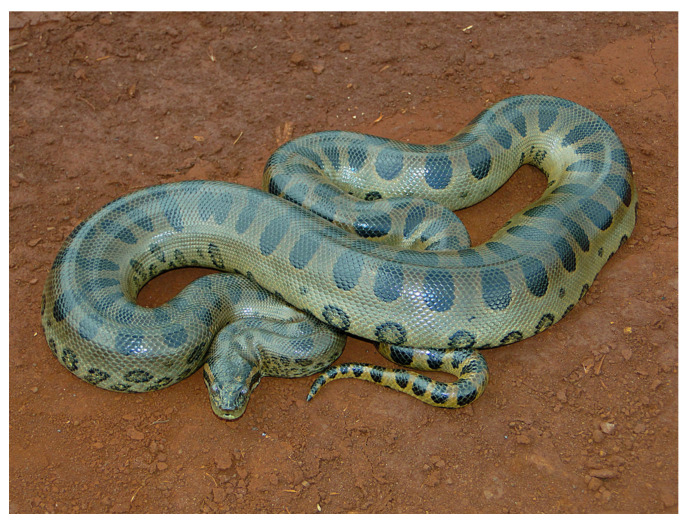
Green anaconda (https://upload.wikimedia.org/wikipedia/commons/b/b4/Sucuri_verde.jpg (accessed on 20 February 2023)) taken from: free media Wikimedia Commons.

The green anaconda (Eunectes murinus) is a boa species that lives in South America, which is also known by other names such as common anaconda, giant anaconda, common water boa, or sucuri. The green anaconda, one of the longest and heaviest snakes in existence, is similar to other boas and is a non-venomous constrictor [56]. The length of green anacondas has been reported to be as long as 5.21 m [57]. In general, the female species with an average length of 4.6 m is usually much larger than the male species with an average length of 3 m [58]. The weight of green anacondas is reported to be between 30 and 70 kg [59]. Green anacondas are olive green in color and have black blotches along their body. Compared to their body size, they have a narrower head that is distinguished by orange–yellow striping. Green anacondas’ eyes are located on its head, giving it the ability to emerge from the water while swimming without exposing its body. Green anacondas have flexible jaw bones that enable it to swallow prey that is larger than the head size of this animal [60]. A picture of a green anaconda is shown in Figure 1.

Green anacondas have a varied diet that they provide by hunting prey. This diet includes fish, birds, reptiles (caimans and turtles), and mammals (agoutes, pacas, tapirs, capybara, peccaries, deer, etc.). There are also reports that green anacondas feed by hunting prey animals over 40 kg, which rarely happens [61]. Green anacondas spend most of their time in or around water. Although they are slow on land, they are very agile in water and are able to swim at high speeds. The green anaconda’s hunting strategy is to hide under the surface of the water while its snouts are placed above the surface of the water. When the prey approaches it or stops to drink water, the green anaconda strikes the prey, wraps around the prey, then contracts to suffocate the prey, and finally swallows it [62].

When the mating season arrives, males look for females. Normally, males are able to identify the position of females and move towards them by following a trail of pheromones that females produce and leave in their path. During this process, males are able to sense chemicals that indicate the presence of the female species by constantly flicking their tongues [63].

Among the natural behaviors of green anacondas in nature, the process of chasing female species by male species during the mating season and their strategy during hunting are much more significant. These natural behaviors of green anacondas are intelligent processes whose mathematical modeling is employed in designing the proposed GAO approach.

### 3.2. Algorithm Initialization

The proposed GOA is a population-based metaheuristic algorithm in which green anacondas are its population members. From a mathematical point of view, each green anaconda is a candidate solution to the problem whose position in the search space determines the values of the decision variables. Hence, each green anaconda can be modeled using a vector, and the population of green anacondas consisting of these vectors can be modeled using a matrix according to Equation (1). The initial position of each green anaconda in the search space is randomly generated at the beginning of the algorithm execution using Equation (2).

(1) $$X={\left[\begin{array}{c} X_{1}\\ \vdots \\ X_{i}\\ \vdots \\ X_{N} \end{array}\right]}_{N\times m}={\left[\begin{array}{ccccc} x_{1,1} & \cdots & x_{1,d} & \cdots & x_{1,m}\\ \vdots & \ddots & \vdots & ⋰ & \vdots \\ x_{i,1} & \cdots & x_{i,d} & \cdots & x_{i,m}\\ \vdots & ⋰ & \vdots & \ddots & \vdots \\ x_{N,1} & \cdots & x_{N,d} & \cdots & x_{N,m} \end{array}\right]}_{N\times m},$$

(2) $$x_{i,d}=lb_{d}+r_{i,d}\cdot \left(ub_{d}-lb_{d}\right),\;i=1,2,\ldots ,N,\;d=1,2,\ldots ,m,$$

where X is the GAO population matrix, $X_{i}$ is the ith green anaconda (candidate solution), $x_{i,d}$ is its dth dimension in the search space (decision variable), N is the number of green anacondas, m is the number of decision variables, $r_{i,d}$ are random numbers in interval $\left[0,1\right]$, and $lb_{d}$ and $ub_{d}$ are the lower bound and upper bound of the dth. decision variable, respectively.

Corresponding to the suggested values of each green anaconda for the decision variables, the objective function of the problem can be evaluated. This set of calculated values for the objective function can be represented from a mathematical point of view using a vector according to Equation (3).

(3) $$F={\left[\begin{array}{c} F_{1}\\ \vdots \\ F_{i}\\ \vdots \\ F_{N} \end{array}\right]}_{N\times 1}={\left[\begin{array}{c} F(X_{1})\\ \vdots \\ F(X_{i})\\ \vdots \\ F(X_{N}) \end{array}\right]}_{N\times 1},$$

where F is the vector of the calculated objective function and $F_{i}$ is the calculated objective function based on the ith green anaconda.

From the comparison of the calculated values for the objective function, the member corresponding to the best value calculated for the objective function is identified as the best member (the best candidate solution). Since in each iteration of GAO, the positions of the green anacondas and thus the values of the objective function are updated, the best member should also be updated.

### 3.3. Mathematical Modelling of GAO

In the GAO design, the position of green anacondas in the search space has been updated based on the simulation of green anaconda behavior in two phases with the aim of providing exploration and exploitation in the search process.

#### 3.3.1. Phase 1: Mating Season (Exploration)

During the mating season, green anaconda female species leave pheromones along their path so that the male species can identify their position. Males use their tongues to sense the chemical effects of pheromones that indicate the presence of a female species and move toward it. In the first phase of GAO, the position of green anacondas is updated based on the male species’ strategy in identifying the female species’ position and moving towards them during the mating season. This strategy leads to large displacements in the position of green anacondas in the search space, which demonstrates the exploration ability of GAO in global search and accurate scanning of the problem-solving space to avoid becoming stuck in optimal local regions.

In order to mathematically simulate this process, it is assumed in the GAO design that for each green anaconda, members of the GAO population who have a better objective function value than it are considered as the female species of green anacondas. The set of candidate female species for each green anaconda is determined using Equation (4).

(4) $$CFL^{i}=\left\{X_{k_{i}}:F_{k_{i}}<F_{i}\;and\;k_{i}\neq i\right\},\;where\;i=1,2,\ldots ,N\;and\;k_{i}\in \left\{1,2,\ldots ,N\right\},$$

where $CFL^{i}$ is the set of candidate females’ locations for the ith green anaconda and $k_{i}$ is the green anaconda row number in the GAO population matrix and the position number of the corresponding element in the objective function vector that has a better objective function value than the ith green anaconda.

The concentration of pheromones has a significant effect on the movement of green anacondas. To simulate the pheromone concentration, the objective function values have been used. Thus, the better the value of the objective function of a member, the higher the chance of selecting it by green anaconda. The probability function of pheromone concentration for the material species corresponding to each GAO member is calculated using Equation (5).

(5) $$PC_{j}^{i}=\frac{{CFF}_{j}^{i}-{CFF}_{max}^{i}}{\sum_{n=1}^{n_{i}}{CFF}_{n}^{i}-{CFF}_{max}^{i}},\;where\;i=1,2,\ldots ,N\;and\;j=1,2,\ldots ,n_{i}$$

where $PC_{j}^{i}$ is the probability of the pheromone concentration of the jth female for the ith green anaconda, ${CFF}^{i}$ is the vector of the set of objective function values of candidate females for the ith green anaconda, ${CFF}_{j}^{i}$ is its jth value, ${CFF}_{max}^{i}$ is its maximum value, and $n_{i}$ is the number of candidate females for the ith green anaconda.

In the GAO design, it is assumed that the green anaconda randomly selects one of the candidate materials and moves towards it. In order to simulate this selection process, first the cumulative probability function of candidate females is calculated using Equation (6). Then, based on the comparison of the cumulative probability function with a random number with a normal distribution in the range of $\left[0,1\right]$, the selected female species for green anaconda is determined according to Equation (7).

(6) $$C_{j}^{i}={PC_{j}^{i}+C}_{j-1}^{i},\;where\;i=1,2,\ldots ,N,\;j=1,2,\ldots ,m,\;and\;C_{0}^{i}=0$$

(7) $$SF^{i}=CFL_{j}^{i}:C_{j-1}^{i}<r_{i,j}<C_{j}^{i}$$

where $C_{j}^{i}$ is the cumulative probability function of the jth candidate female for the ith green anaconda, $SF^{i}$ is the selected female for the ith green anaconda, and r is a random number with a normal distribution in the range of $\left[0,1\right]$.

After determining the selected female species, based on the simulation of green anaconda movement towards it, a random position in the search space for green anaconda is calculated using Equation (8). If the value of the objective function is improved in this new position, according to Equation (9), the position of corresponding green anaconda is updated to this new position, otherwise it remains in the previous position.

(8) $$x_{i,d}^{P1}=x_{i,d}+r_{i,d}\cdot \left(SF_{d}^{i}-I_{i,d}\cdot x_{i,d}\right),\;i=1,2,\ldots ,N,\;and\;d=1,2,\ldots ,m,$$

(9) $$X_{i}=\left\{\begin{array}{c} X_{i}^{P1},\;F_{i}^{P1}<F_{i},\\ X_{i},\;\;else, \end{array}\right.$$

where $X_{i}^{P1}$ is the new suggested position of the ith green anaconda based on the first phase of GAO, $x_{i,d}^{P1}$ is its dth dimension, $F_{i}^{P1}$ is its objective function value, $r_{i,d}$ are random numbers with a normal distribution in the range of $\left[0,1\right]$, $SF_{d}^{i}$ is the dth dimension of the selected female for the ith green anaconda, $I_{i,d}$ are random numbers from the set $\left\{1,2\right\}$, N is the number of green anacondas, and m is the number of decision variables.

#### 3.3.2. Phase 2: Hunting Strategy (Exploitation)

Green anacondas are powerful predators whose hunting strategy is to ambush underwater and wait for prey. When the prey stops drinking water or passes near the green anaconda, the anaconda attacks and surrounds the prey, then contracts to suffocate the prey, and finally swallows it. In the second phase of GAO, the position of the population members is updated based on the green anaconda’s strategy when hunting prey. This strategy leads to small displacements in the position of the green anacondas in the search space, which indicates the exploitation ability of GAO in local search to obtain possible better solutions near the discovered solutions.

In order to simulate the hunting strategy and change the position of the population members towards the prey that has approached them, first a random position is generated near each green anaconda using Equation (10). Then, according to Equation (11), if the value of the objective function is improved in this new position, it is acceptable to update the green anaconda location.

(10) $$x_{i,d}^{P2}=x_{i,d}+\left(1-2r_{i,d}\right)\frac{ub_{d}-lb_{d}}{t},\;i=1,2,\ldots ,N,\;d=1,2,\ldots ,m,\;and\;t=1,2,\ldots ,T$$

(11) $$X_{i}=\left\{\begin{array}{c} X_{i}^{P2},\;F_{i}^{P2}<F_{i},\\ X_{i},\;\;else, \end{array}\right.$$

where $X_{i}^{P2}$ is the new suggested position of the ith green anaconda based on thr second phase of GAO, $x_{i,d}^{P2}$ is its dth dimension, $F_{i}^{P2}$ is its objective function value, t is the iteration counter of the algorithm, and T is the maximum number of algorithm iterations.

### 3.4. Repetition Process, Pseudocode, and Flowchart of GAO

**Figure 2:**
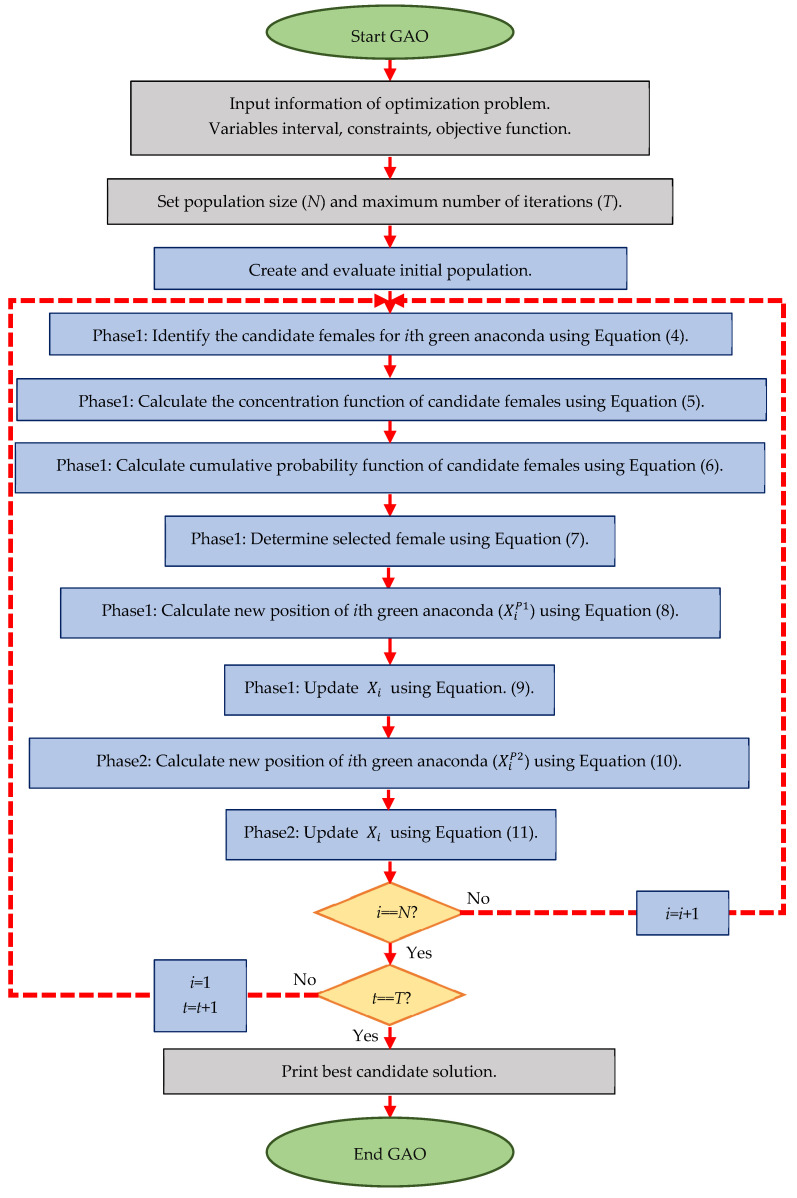
Flowchart of GAO.

Various steps of GAO are presented in the form of a flowchart in Figure 2 and its pseudocode is presented in Algorithm 1. The first iteration of GAO is completed after updating the position of all green anacondas based on the first and second phases. After this, the algorithm enters the next iteration with the new values of the objective function and the new positions of the green anacondas, and the updating process continues according to Equations (4)–(11) until the last iteration of the algorithm. After the full implementation of GAO, the best candidate solution recorded during the execution of the algorithm is presented as a solution for the given problem.
**Algorithm 1.** Pseudocode of GAOStart GAO.1.Input problem information: variables, objective function, and constraints.2.Set GAO population size (*N*) and iterations (*T*).3.Generate the initial population matrix at random using Equation (2). $x_{i,d}\leftarrow lb_{d}+r_{i,d}\cdot (ub_{d}-lb_{d})$4.Evaluate the objective function.5.For t=1 to *T*6.For i=1 to N7.Phase 1: mating season (exploration)8.Identify the candidate females using Equation (4). $CFL^{i}\leftarrow \left\{X_{k_{i}}:F_{k_{i}}<F_{i}\;and\;k_{i}\neq i\right\}.$9.Calculate the concentration function of candidate females using Equation (5). $PC_{j}^{i}\leftarrow \frac{{CFF}_{j}^{i}-{CFF}_{max}^{i}}{\sum_{n=1}^{n_{i}}{CFF}_{n}^{i}-{CFF}_{max}^{i}}.$10.Calculatethe cumulative probability function candidate females using Equation (6). $C_{j}^{i}\leftarrow {PC_{j}^{i}+C}_{j-1}^{i}.$11.Determine the selected female using Equation (7). $SF^{i}\leftarrow CFL_{j}^{i}:C_{j-1}^{i}<r_{i,j}<C_{j}^{i}.$12.Calculate the new position of *i*th GAO member using Equation (8). $x_{i,d}^{P1}\leftarrow x_{i,d}+r_{i,d}\cdot \left(SF_{d}^{i}-I_{i,d}\cdot x_{i,d}\right).$13.Update *i*th GAO member using Equation (9). $X_{i}\leftarrow \left\{\begin{array}{c} X_{i}^{P1},\;F_{i}^{P1}<F_{i},\\ X_{i},\;\;else. \end{array}\right.$14.Phase 2: hunting strategy (exploitation)15.Calculate the new position of *i*th GAO member using Equation (10). $x_{i,d}^{P2}\leftarrow x_{i,d}+(1-2r_{i,d})\frac{ub_{d}-lb_{d}}{t}$16.Update the ith GAO member using Equation (11). $X_{i}\leftarrow \left\{\begin{array}{c} X_{i}^{P2},\;F_{i}^{P2}<F_{i},\\ X_{i},\;\;else. \end{array}\right.$17.End18.Save the best candidate solution so far.19.End20.Output the best quasi-optimal solution obtained with the GAO.End GAO.

### 3.5. Computational Complexity of GAO

In this section, the computational complexity of the proposed GAO approach is evaluated. GAO initialization for a problem with *m* number of decision variables is O(Nm) where *N* is the number of green anacondas. In each iteration of GAO, the position of green anacondas is updated in two different phases, and this process has a computational complexity equal to O(2NmT), where *T* is the maximum iterations of the algorithm. Therefore, the total computational complexity of GAO is equal to O(Nm(1+2T)).

## 4. Simulation Studies and Results

GAO’s ability to solve optimization problems has been evaluated in this section on a set of thirty-nine benchmark functions from the CEC 2017 test suite and CEC 2019 test suite. The CEC 2017 test suite includes 30 objective functions, among which C17-F1 to C17-F3 are unimodal, C17-F4 to C17-F10 are multimodal, C17-F11 to C17-F20 are hybrid, and C17-F21 to C17-F30 are composition. From this set, the function C17-F2 has been left out from the simulations due to the instability of the behavior. The full description of the CEC 2017 test suite is provided in [64]. The CEC 2019 test suite includes ten complex objective functions, the full description of which is provided in [65]. The performance of GAO in optimization is compared with twelve well-known metaheuristic algorithms including: GA [25], PSO [14], GSA [28], TLBO [41], MVO [39], GWO [15], WOA [22], MPA [18], TSA [24], RSA [20], AVOA [17], and WSO [19]. The values used for the control parameters of competitor algorithms are shown in Table 1.

GAO and competing algorithms have been implemented on the mentioned thirty-nine benchmark functions in order to obtain suitable solutions for these functions. The simulation results are presented using six indicators: mean, best, worst, standard deviation (std), median, and rank.

### 4.1. Evaluation the CEC 2017 Test Suite

**Figure 3:**
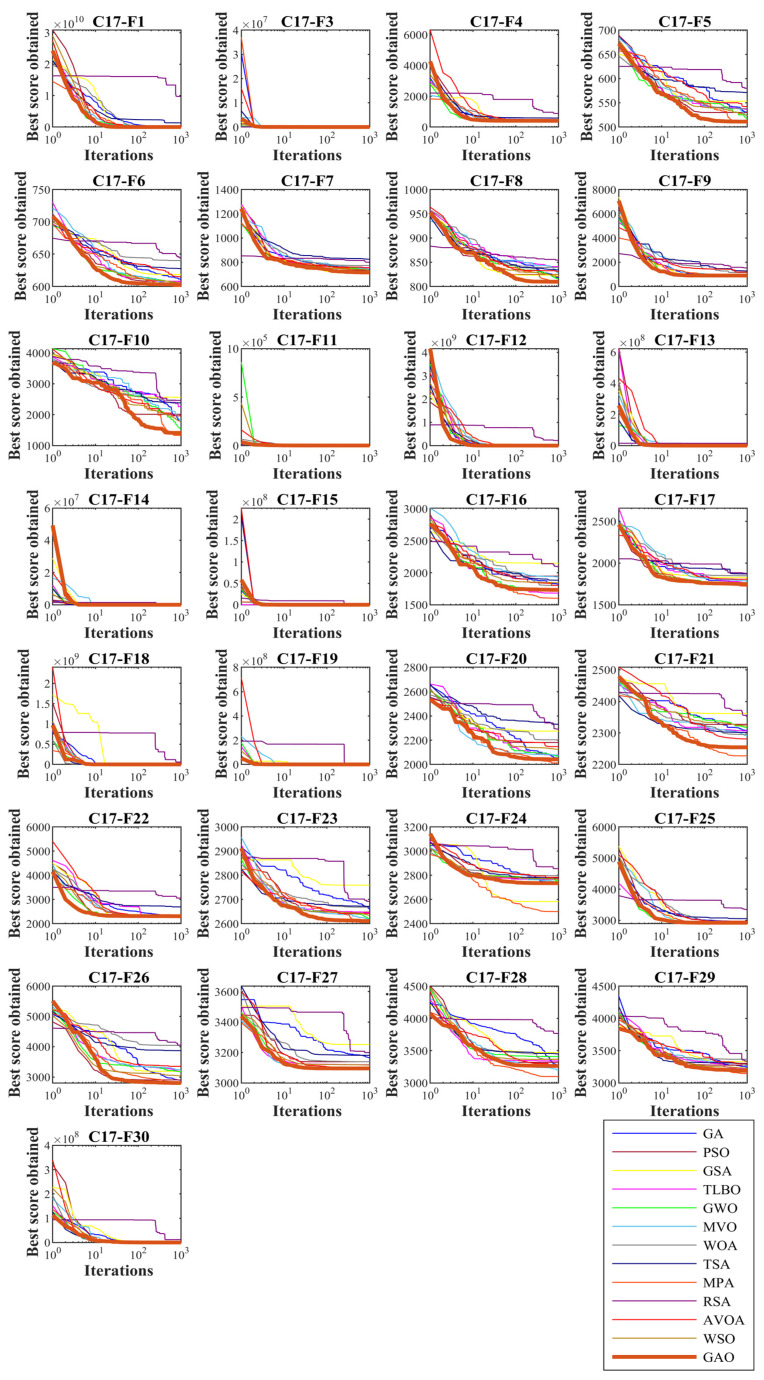
Convergence curves of GAO and competitor algorithms performance on the CEC 2017 test suite (dimension m=10).

**Figure 4:**
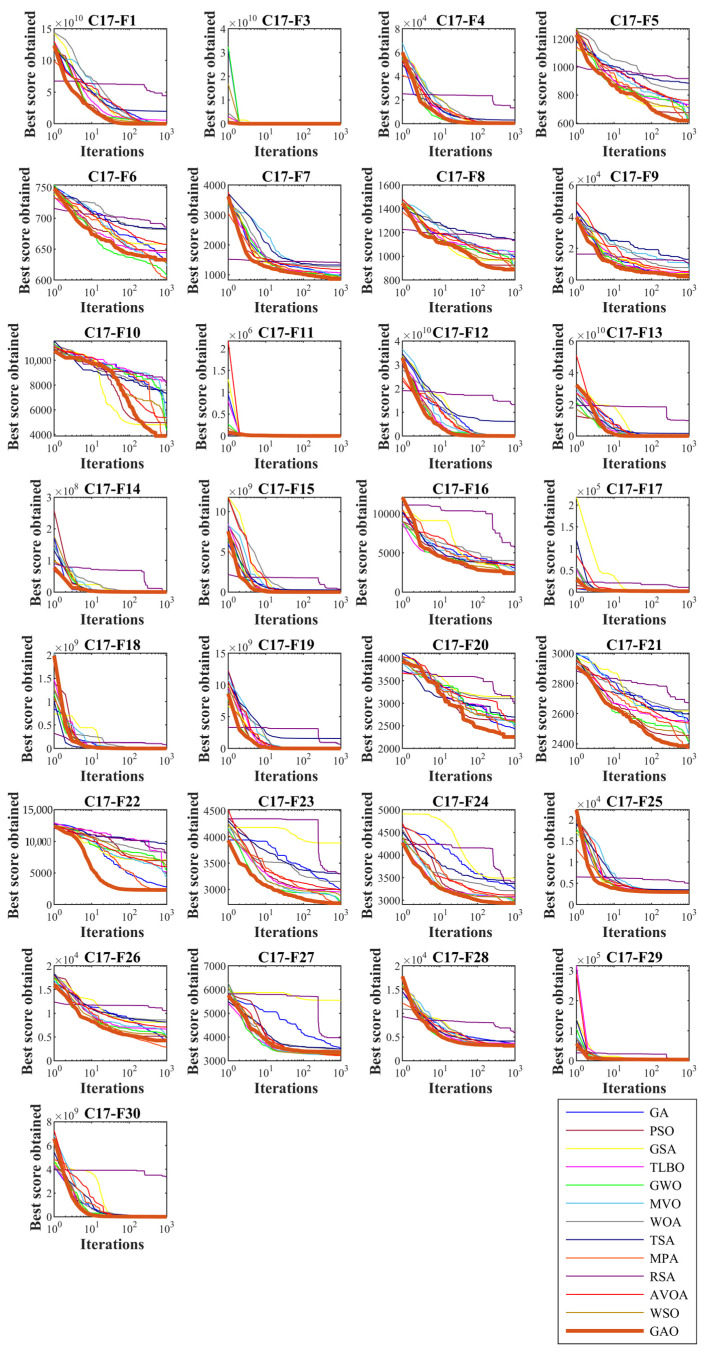
Convergence curves of GAO and competitor algorithms performance on the CEC 2017 test suite (dimension m=30).

**Figure 5:**
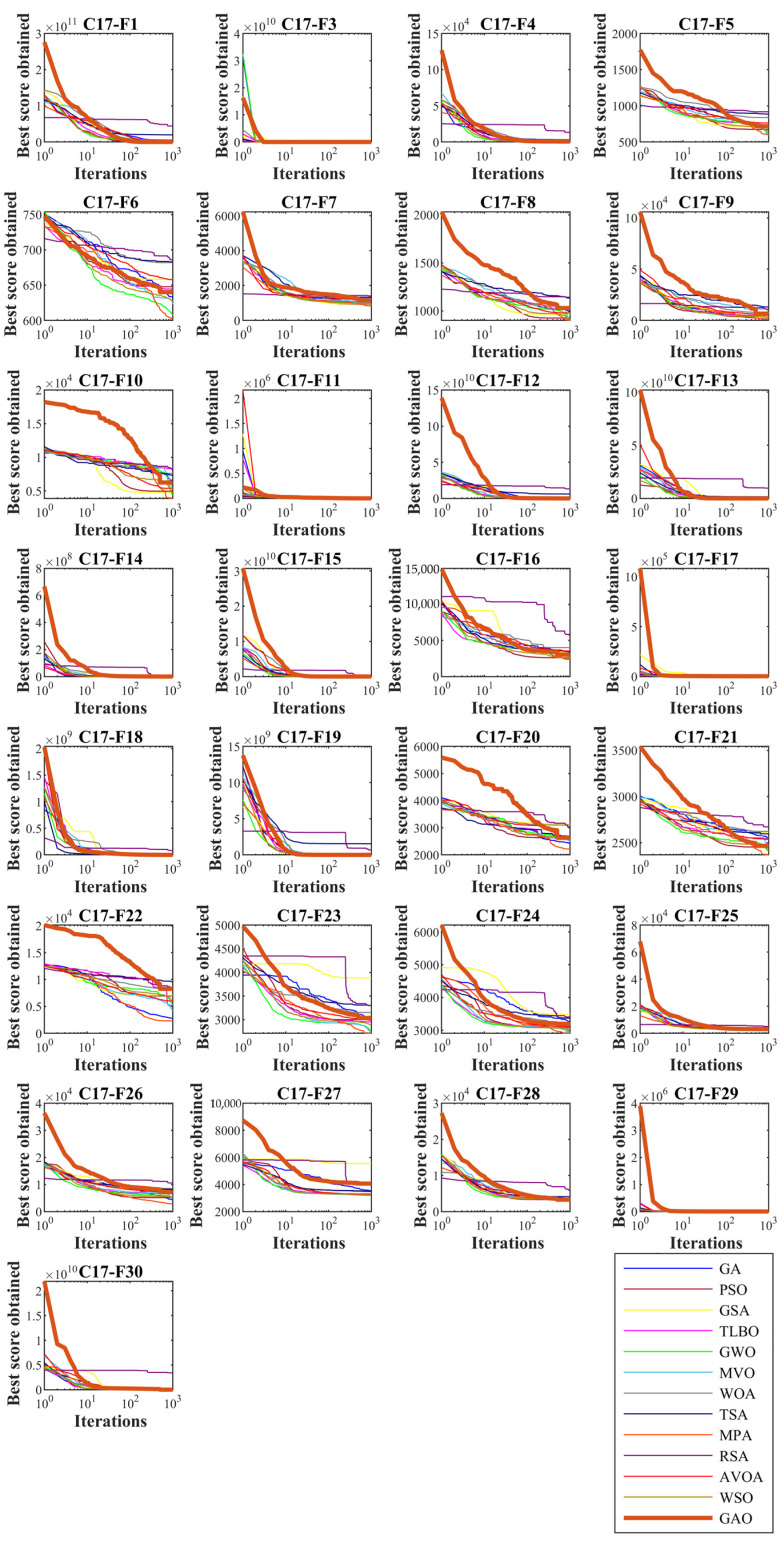
Convergence curves of GAO and competitor algorithms performance on the CEC 2017 test suite (dimension m=50).

**Figure 6:**
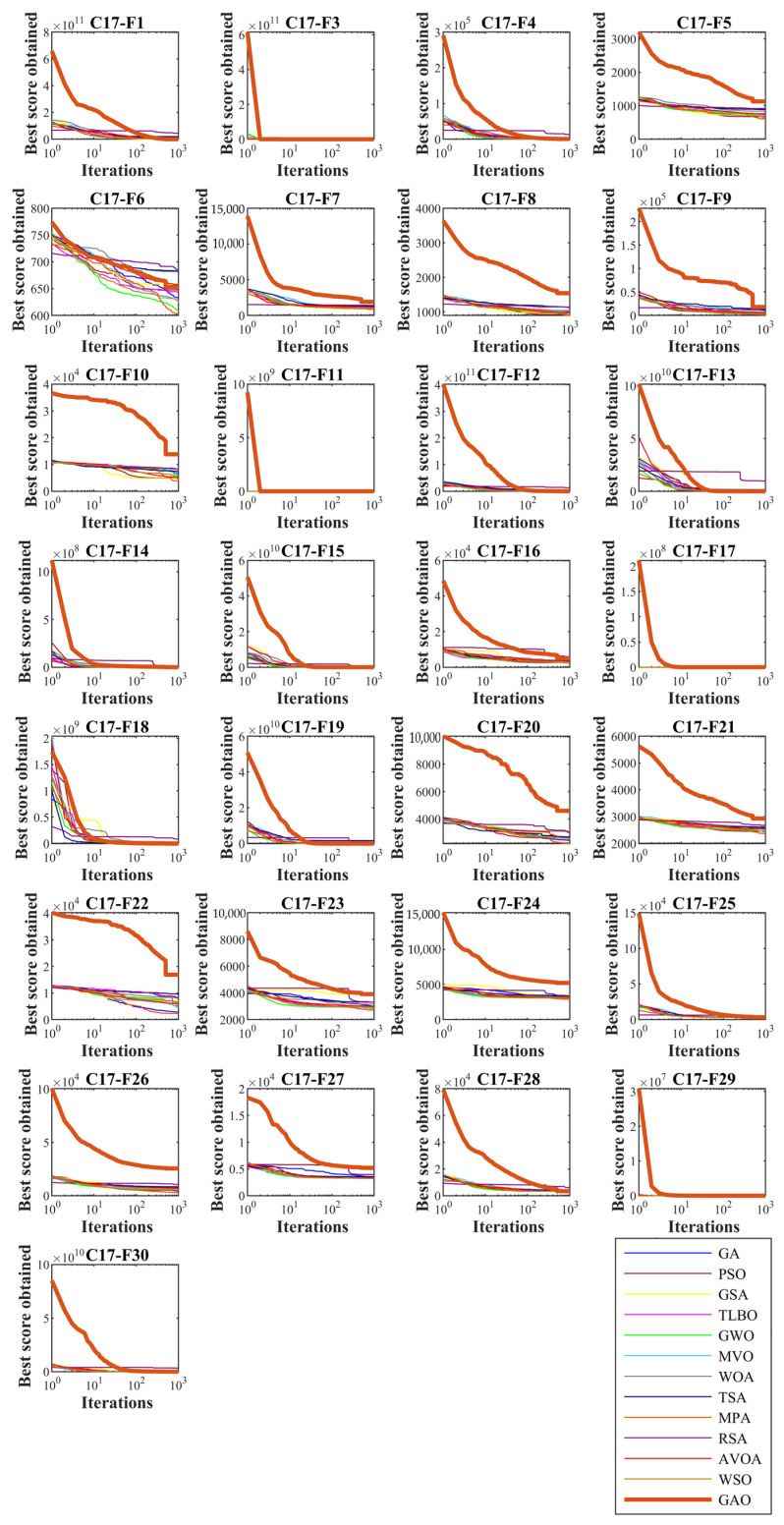
Convergence curves of GAO and competitor algorithms performance on CEC 2017 test suite (dimension m=100).

In this subsection, GAO’s ability to solve optimization problems is tested on the CEC 2017 test suite. In order to analyze the scalability, GAO and competitor algorithms are employed to optimize this set for different dimensions equal to 10, 30, 50, and 100. The simulation results are reported in Table 2, Table 3, Table 4 and Table 5. Convergence curves of the performance of GAO and competitor algorithms on the CEC 2017 test suite for different dimensions are presented in Figure 3, Figure 4, Figure 5 and Figure 6. Simulation results for dimension equal to 10 show that GAO is the first best optimizer for C17-F1, C17-F3, C17-F4, C17-F7, C17-F9, C17-F10, C17-F12 to C17-F14, C17-F16, C17-F18, C17-F19, C17-F21, C17-F22, C17-F25, C17-F26, and C17-F29 compared to competitor algorithms. For dimension equal to 30, GAO is the first best optimizer for C17-F1, C17-F3 to C17-F5, C17-F7, C17-F12 to C17-F14, C17-F16 to C17-F18, C17-F21 to C17-F27, and C17-F29 compared to competitor algorithms. For dimension equal to 50, GAO is the first best optimizer for C17-F1, C17-F3 to C17-F14, C17-F16 to C17-F18, C17-F20, C17-F22 to C17-F26, C17-F28, and C17-F30 compared to competitor algorithms. For dimension equal to 100, GAO is the first best optimizer for C17-F1, C17-F3 to C17-F13, C17-F15 to C17-F23, C17-F26, C17-F27, C17-F29, and C17-F30 compared to competitor algorithms.

The unimodal functions C11-F1 and C11-F3 do not have local optimal. For that reason, they are suitable criteria for measuring the exploitation ability of metaheuristic algorithms in local search and convergence to the global optimal. The optimization results of C17-F1 and C17-F3 functions show that the proposed GAO approach has a high ability in exploitation. Multi-modal functions C17-F4 to C17-F10 have several local optimal in addition to the main optimal. For this reason, they are suitable criteria to measure the exploitation ability of metaheuristic algorithms in the global search and discover the main optimal area. The simulation results show that GAO has a high quality in exploration and decent performance in solving multi-modal functions. Hybrid functions C17-F11 to C17-F20 and composition functions C17-F21 to C17-F30 are suitable options for measuring the ability of metaheuristic algorithms to balance exploration and exploitation during the search process. The optimization results show that GOA achieves acceptable results for these benchmark functions. Thus it can be said that GAO can balance exploration and exploitation in the optimization process. It can be inferred from the simulation results that the proposed GAO approach by balancing exploration and exploitation has performed better compared to competing algorithms in optimizing the CEC 2017 test suite for different dimensions of 10, 30, 50, and 100, and overall, it has been ranked as the best optimizer.

### 4.2. Evaluation the CEC 2019 Test Suite

**Figure 7:**
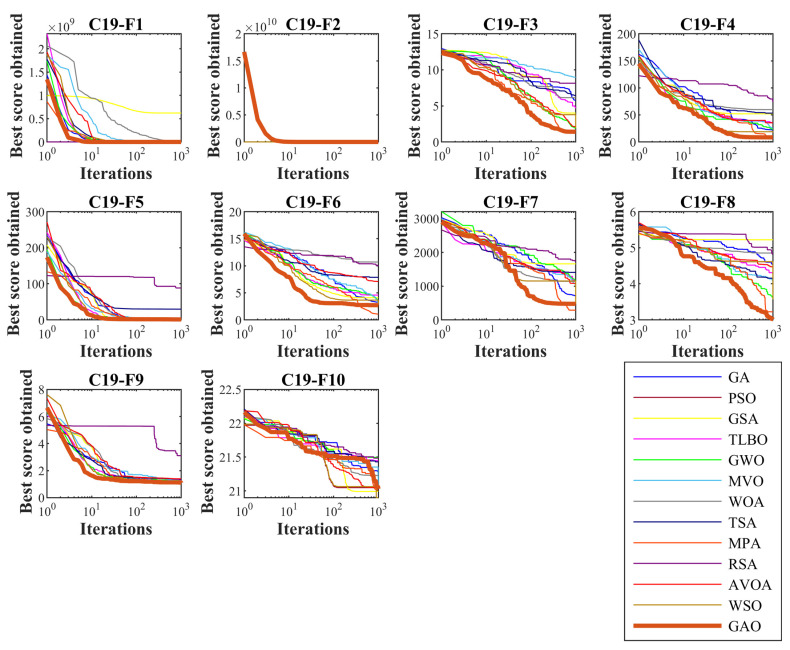
Convergence curves of GAO and competitor algorithms performance on the CEC 2019 test suite.

In this subsection, GAO’s ability to solve optimization problems has been tested on the CEC 2019 test suite. This test suite has ten benchmark functions and its dimensions are 9 for C19-F1, 16 for C19-F2, 18 for C19-F3, and 10 for C19-F4 to C19-F10. The full description and details of the CEC 2019 test suite are provided in [65]. The results of employing the proposed GAO approach and competitor algorithms in dealing with this test suite are reported in Table 6. The simulation results show that GAO is the first best optimizer for C19-F1 to C19-F4, and C19-F6 to C19-F9 compared to the competitor algorithms. Analysis of the simulation results indicates that the proposed GAO approach has performed better by balancing exploration and exploitation compared to the competitor algorithms and has been assigned the first rank of the best optimizer in handling the CEC 2019 test suite. Convergence curves of performance for the GAO and competitor algorithms during optimization of the CEC 2019 test suite are presented in Figure 7.

### 4.3. Statistical Analysis

Presenting the optimization results of the objective functions using mean, best, worst, standard deviation, median, and rank indicators provides valuable information about the performance of metaheuristic algorithms and the proposed GAO approach. However, statistical analysis is necessary to show whether the superiority of GAO’s proposed approach against competitor algorithms is significant or not. In order to deal with this issue, the Wilcoxon rank sum test [66], which is a non-parametric test and is used to determine the significant difference between two data samples, is used. Results of implementing the Wilcoxon rank sum test analysis on the simulation results of the CEC 2017 test suite and the CEC 2019 test suite are reported in Table 7. The results obtained for the *p*-value index indicate that the proposed GAO approach has a significant statistical superiority in comparison to the corresponding competitor algorithms in cases where the p-value is less than 0.05.

## 5. GAO for Real-World Applications

**Figure 8:**
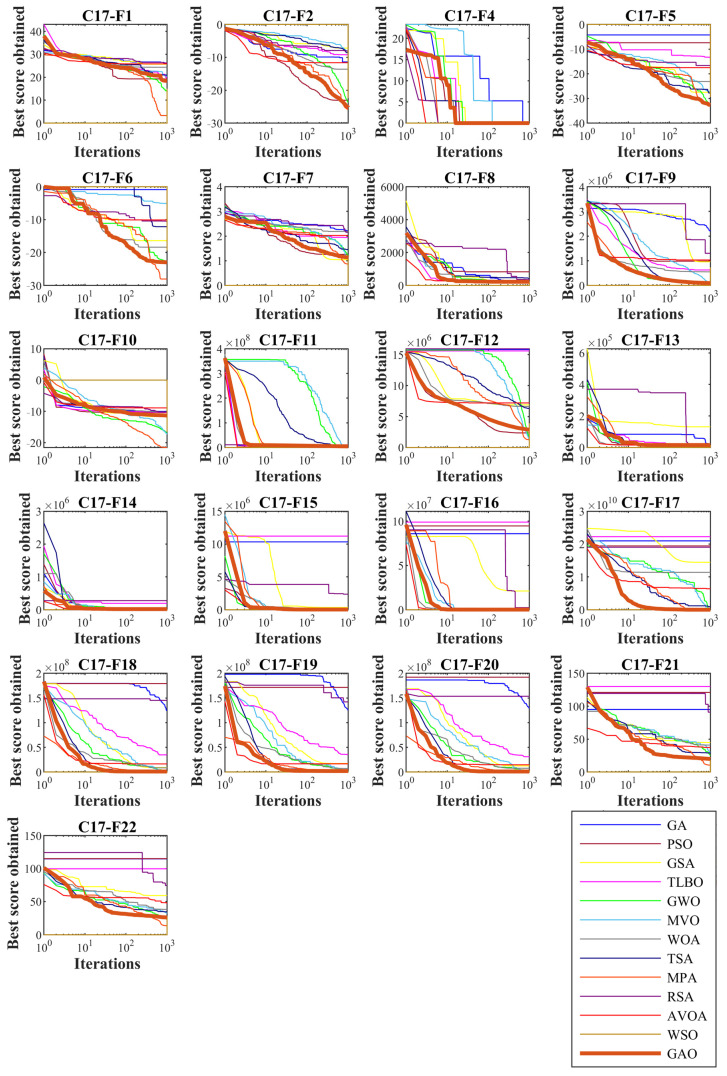
Convergence curves of GAO and competitor algorithms performance on the CEC 2011 test suite.

In this section, the ability of the proposed GAO approach to handle optimization problems in real-world applications is evaluated. For this purpose, the CEC 2011 test suite, which is a collection of 22 real-world optimization applications, is employed. The titles of these real-world optimization problems are as follows: parameter estimation for frequency-modulated (FM) sound waves, the Lennard–Jones potential problem, the bifunctional catalyst blend optimal control problem, optimal control of a non-linear stirred tank reactor, tersoff potential for model Si (B), tersoff potential for model Si (C), spread spectrum radar polly phase code design, the transmission network expansion planning (TNEP) problem, the large-scale transmission pricing problem, the circular antenna array design problem, the ELD problems (consisting of: DED instance 1, DED instance 2, ELD Instance 1, ELD Instance 2, ELD Instance 3, ELD Instance 4, ELD Instance 5, hydrothermal scheduling instance 1, hydrothermal scheduling instance 2, and hydrothermal scheduling instance 3), the messenger: spacecraft trajectory optimization problem, and the Cassini 2: spacecraft trajectory optimization problem. From this set, the C11-F3 function has been removed in the simulation studies. The full description of the CEC 2011 test suite is provided in [67]. The results of implementing the proposed GAO approach and competitor algorithms on the CEC 2011 test suite are reported in Table 8. The simulation results show that GAO is the first best optimizer for C11-F1, C11-F2, C11-F4 to C11-F7, C11-F9 to C11-F12, FC11-14 to C11-F16, and C11-F20 to C11-F22 compared to the competing algorithms. What is clear from the analysis of the simulation results is that the proposed GAO approach has effective performance in dealing with real-world applications and based on the Wilcoxon rank sum test statistical analysis, it has won the first rank of being the best optimizer compared to the competitor algorithms. The convergence curves of the performance of GAO and competitor algorithms during optimization of the CEC 2011 test suite are presented in Figure 8.

## 6. Conclusions and Future Works

A new swarm-based optimization algorithm, called green anaconda optimization (GAO), that can be used in solving optimization problems, is introduced in this paper. The natural behavior of green anacondas, the strategy of the male species in identifying the position of the female species during the mating season, and their hunting strategy are the fundamental inspiration for GAO. The mathematical model of GAO is presented in two phases of exploration and exploitation based on modeling the natural behavior of green anacondas. The ability of the proposed GAO approach in handling the optimization problems is tested on thirty-nine objective functions from the CEC 2017 and CEC 2019 test suites. The performance of GAO is also compared with that of twelve well-known metaheuristic algorithms. The simulation results show that the proposed GAO approach has superior performance compared to competitor algorithms by creating a balance between exploration and exploitation. In addition, the implementation of GAO on twenty-one problems from the CEC 2011 test suite showed the high capability of the proposed approach in handling real-world applications.

Among several suggestions for future research, the design of binary and multi-objective versions of the proposed GAO approach is the most prominent. Employing the proposed GAO approach in order to solve optimization problems in various sciences as well as real-world applications such as image clustering, image segmentation, medical applications, and engineering problems are other suggestion for future research.

## Figures and Tables

**Table 1:** Control parameters values.

Algorithm	Parameter	Value
GA		
	Type	Real coded
	Selection	Roulette wheel (proportionate)
	Crossover	Whole arithmetic (probability = 0.8, $\alpha \in \left[-0.5,1.5\right]$)
	Mutation	Gaussian (probability = 0.05)
PSO		
	Topology	Fully connected
	Cognitive and social constant	(*C*_1_, *C*_2_) =(2,2)
	Inertia weight	Linear reduction from 0.9 to 0.1
	Velocity limit	10% of dimension range
GSA		
	Alpha, *G*_0_, *R_norm_*, *R_power_*	20, 100, 2, 1
TLBO		
	*T_F_*: Teaching factor	*T_F_* = round $\left[\left(1+rand\right)\right]$
	random number	*rand* is a random number between $\left[0-1\right].$
GWO		
	Convergence parameter (*a*)	*a*: Linear reduction from 2 to 0.
MVO		
	Wormhole existence probability (WEP)	Min(WEP) = 0.2 and Max(WEP) = 1.
	Exploitation accuracy over the iterations (*p*)	p=6.
WOA		
	Convergence parameter (*a*)	*a*: Linear reduction from 2 to 0.
	*r* is a random vector in $\left[0-1\right].$	
	*l* is a random number in $\left[-1,1\right].$	
TSA		
	P_min_ and P_max_	1, 4
	*c*1, *c*2, *c*3	random numbers lie in the range of $\left[0-1\right].$
MPA		
	Constant number	P=0.5
	Random vector	*R* is a vector of uniform random numbers in $\left[0,1\right].$
	Fish aggregating devices (*FADs*)	FADs=0.2
	Binary vector	U=0 or 1
RSA		
	Sensitive parameter	β=0.01
	Sensitive parameter	α=0.1
	Evolutionary sense (ES)	ES: randomly decreasing values between 2 and −2
AVOA		
	L_1_, L_2_	0.8, 0.2
	W	2.5
	P_1_, P_2_, P_3_	0.6, 0.4, 0.6
WSO		
	F_min_ and F_max_	0.07, 0.75
	*τ*, *a*_o_, *a*_1_, *a*_2_	4.125, 6.25, 100, 0.0005

**Table 2:** Optimization results of the CEC 2017 test suite (dimension m=10).

		GAO	WSO	AVOA	RSA	MPA	TSA	WOA	MVO	GWO	TLBO	GSA	PSO	GA
C17-F1	mean	100	6977.111	1822.634	9.72 × 10^9^	104.6622	1.33 × 10^9^	7,296,302	11,776.52	18,952.96	1.57 × 10^8^	328.9346	3347.233	20,023,383
	best	100	345.9705	754.1327	7.3 × 10^9^	102.4063	11,665,981	3,349,288	6355.289	11,697.02	69,930,767	109.1846	362.021	6,675,925
	worst	100	12,471.59	3882.122	1.27 × 10^10^	106.0517	3.8 × 10^9^	10,244,428	15,855.06	28,173.75	3.79 × 10^8^	747.2462	9924.345	36,691,453
	std	1.76 × 10^−5^	6313.97	1440.855	2.69 × 10^9^	1.615877	1.69 × 10^9^	3,442,725	4085.323	7694.896	1.49 × 10^8^	290.3269	4427.81	12,404,106
	median	100	7545.442	1327.14	9.47 × 10^9^	105.0955	7.46 × 10^8^	7,795,746	12,447.86	17,970.54	89,666,699	229.6539	1551.284	18,363,078
	rank	1	6	4	13	2	12	9	7	8	11	3	5	10
C17-F3	mean	300	349.9638	333.4122	11,479.03	300	11,447.37	1003.998	300.0247	3182.624	754.5378	11,414.77	314.1376	15,733.23
	best	300	302.6537	300.001	7421.598	300	7260.632	501.6584	300.0095	612.137	482.5901	9317.412	311.5075	4618.157
	worst	300	394.8162	375.47	15,834.53	300	15,343.09	1755.01	300.0433	7521.755	932.1847	12,906.36	317.3719	24,879.84
	std	4.64 × 10^−14^	50.70352	31.21843	4674.285	5.43 × 10^−11^	3304.026	552.1851	0.014095	3284.596	197.091	1554.807	2.491209	10,584.32
	median	300	351.1926	329.0889	11,329.99	300	11,592.88	879.6611	300.023	2298.302	801.6882	11,717.65	313.8355	16,717.47
	rank	1	6	5	12	2	11	8	3	9	7	10	4	13
C17-F4	mean	400.002	407.7045	418.5456	881.8049	417.7576	582.4649	430.8554	404.3984	423.2437	409.7871	406.2592	421.6779	415.7083
	best	400	400.0125	401.1698	586.4966	414.2066	406.9075	406.8525	403.2745	407.2344	408.9495	405.0984	400.1128	412.4635
	worst	400.008	424.6187	468.8402	1517.016	423.186	947.3914	456.5312	405.4712	470.4305	410.3159	406.799	475.105	419.6784
	std	0.004024	11.63975	33.53654	427.6431	4.118432	246.3324	27.24021	0.898639	31.45858	0.585883	0.781423	35.98526	3.157633
	median	400	403.0934	402.0862	711.8537	416.8188	487.7803	430.019	404.4239	407.655	409.9415	406.5697	405.7469	415.3457
	rank	1	4	8	13	7	12	11	2	10	5	3	9	6
C17-F5	mean	520.5609	519.1534	542.2855	580.0791	521.6225	571.2951	538.0164	524.0977	517.8573	536.616	553.2224	529.9867	530.1072
	best	507.9597	509.9497	520.8941	561.3768	509.9496	534.1875	514.506	514.9287	510.3157	530.7196	539.7681	511.9395	525.052
	worst	534.983	528.8541	563.6767	591.9163	544.5154	593.0703	562.2668	542.6545	528.6164	540.4401	566.6616	555.7175	536.3365
	std	14.10965	8.72578	17.58362	13.27221	15.63282	25.97608	19.84514	12.60888	8.11889	4.272868	12.55651	20.21898	5.109001
	median	519.6504	518.905	542.2856	583.5117	516.0124	578.9613	537.6464	519.4038	516.2485	537.6521	553.2301	526.145	529.5201
	rank	3	2	10	13	4	12	9	5	1	8	11	6	7
C17-F6	mean	603.01	600.5606	614.929	644.2993	600.0003	627.6556	640.0763	600.5326	601.6492	607.426	618.4222	608.0398	611.1025
	best	601.6233	600.0005	605.396	641.2874	600.0001	612.1762	634.9033	600.2196	601.1107	605.1494	609.0575	601.4659	607.472
	worst	604.6321	602.0417	635.5747	647.7859	600.0004	644.1825	650.7377	601.0412	603.1359	610.9759	629.7345	620.8405	615.6957
	std	1.3499	0.991847	13.93839	2.8917	0.000133	13.3633	7.437235	0.385881	0.991826	2.656369	8.639876	8.788191	3.64533
	median	602.8922	600.1001	609.3725	644.0619	600.0004	627.1318	637.332	600.4348	601.1752	606.7894	617.4483	604.9264	610.6212
	rank	5	3	9	13	1	11	12	2	4	6	10	7	8
C17-F7	mean	714.9746	727.3328	753.8474	799.9577	720.073	824.0926	772.6133	721.9245	730.1478	755.4194	716.7679	734.5213	738.9913
	best	714.4159	716.9793	727.955	790.3283	715.4835	794.2034	751.0108	711.5214	721.8083	750.5447	711.4275	726.7135	727.7346
	worst	715.7112	737.1411	801.0858	810.4362	724.3538	863.1991	802.0857	728.4637	749.7124	764.2491	725.9128	747.0726	743.9886
	std	0.628096	8.631729	32.39556	8.435769	3.767197	28.68428	25.18788	7.555293	13.11915	6.146001	6.368765	9.270855	7.625192
	median	714.8856	727.6054	743.1745	799.5332	720.2273	819.4839	768.6783	723.8564	724.5352	753.4418	714.8658	732.1495	742.121
	rank	1	5	9	12	3	13	11	4	6	10	2	7	8
C17-F8	mean	818.8348	817.5792	830.8799	854.2832	833.2392	834.1207	839.1509	827.6156	815.564	840.7119	823.3815	824.5363	818.0629
	best	804.9748	807.9599	822.034	840.3486	809.9496	812.4518	825.8119	808.9582	810.3711	833.2718	815.9193	816.9143	813.7883
	worst	851.46	841.4628	837.8083	860.4043	863.7533	856.0983	854.1837	855.7292	822.351	849.4086	831.8386	831.483	826.546
	std	21.88629	16.01194	8.152015	9.478211	26.18452	18.60264	11.62491	19.87598	5.278038	8.256642	7.685449	7.212869	5.753112
	median	809.4521	810.4471	831.8386	858.19	829.6269	833.9664	838.3041	822.8875	814.767	840.0837	822.884	824.8739	815.9586
	rank	4	2	8	13	9	10	11	7	1	12	5	6	3
C17-F9	mean	900	958.0246	1183.273	1521.471	900	1272.429	1145.44	900.1154	900.5917	912.8054	926.2937	904.5932	905.5344
	best	900	906.8134	1031.947	1377.821	900	931.1214	1005.691	900.0008	900.0569	907.8307	920.4252	900.9737	903.0297
	worst	900	1056.588	1387.365	1784.32	900	1701.296	1460.087	900.4561	900.9171	921.6602	931.8898	913.3387	909.8295
	std	0	70.4426	148.8524	184.2871	6.63 × 10^−8^	336.066	211.231	0.227138	0.376198	6.077825	5.95763	5.904611	3.075444
	median	900	934.3484	1156.889	1461.871	900	1228.649	1057.991	900.0023	900.6964	910.8653	926.4299	902.0301	904.6392
	rank	1	9	11	13	2	12	10	3	4	7	8	5	6
C17-F10	mean	1301.559	1482.241	1963.686	2463.959	1343.128	2384.889	2464.88	1794.25	1535.89	2256.198	2564.708	2013.59	1766.646
	best	1148.146	1240.525	1532.027	2279.151	1189.366	2194.314	2123.467	1606.638	1410.302	1836.742	2149.315	1599.934	1443.475
	worst	1457.885	1761.604	2178.649	2817.449	1472.816	2769.978	2906.585	2041.969	1721.165	2565.7	2887.991	2449.501	2191.037
	std	133.5989	219.3419	292.3638	240.3721	116.9823	260.9104	329.2918	213.5238	132.1632	309.9574	332.2394	348.9919	320.4534
	median	1300.103	1463.417	2072.033	2379.618	1355.166	2287.633	2414.733	1764.196	1506.047	2311.176	2610.763	2002.462	1716.037
	rank	1	3	7	11	2	10	12	6	4	9	13	8	5
C17-F11	mean	1110.291	1129.294	1216.327	2847.161	1101.951	3448.614	1271.237	1117.426	1129.288	1154.533	1132.152	1146.624	2474.015
	best	1109.37	1112.659	1137.215	2190.264	1100.106	1227.421	1131.339	1103.416	1113.949	1140.523	1123.071	1134.545	1116.115
	worst	1110.713	1155.055	1382.588	3615.403	1103.709	5693.758	1472.267	1139.468	1144.24	1177.45	1138.753	1169.648	6326.297
	std	0.631109	20.42284	112.1149	598.7897	1.471866	2486.253	151.1479	15.44328	13.40317	15.94047	6.927099	15.80819	2568.811
	median	1110.54	1124.73	1172.752	2791.489	1101.994	3436.639	1240.671	1113.41	1129.481	1150.079	1133.392	1141.152	1226.824
	rank	2	5	9	12	1	13	10	3	4	8	6	7	11
C17-F12	mean	1236.271	5504.867	2,486,523	2.2 × 10^8^	1290.268	271,235.9	8,310,292	182,122.5	1,523,242	5,426,363	526,882.7	8588.212	649,676
	best	1200.472	2544.855	1,327,937	71,378,713	1258.491	90,040.82	1,024,361	52,968.26	348,583.1	1,452,091	86,253.94	2606.745	188,110.4
	worst	1320.393	8397.613	4,285,351	3.92 × 10^8^	1351.6	366,131.1	18,484,802	401,267.3	2,123,298	9,606,371	1,163,364	14,841.02	1,146,930
	std	56.3488	2509.074	1,363,601	1.52 × 10^8^	42.41378	126,890.6	7,346,263	152,346.3	821,342.4	4,319,261	485,164.3	5574.514	393,704.8
	median	1212.11	5538.5	2,166,401	2.08 × 10^8^	1275.49	314,385.8	6,866,002	137,127.3	1,810,543	5,323,495	428,956.2	8452.54	631,832
	rank	1	3	10	13	2	6	12	5	9	11	7	4	8
C17-F13	mean	1304.993	1331.056	8036.958	14,569,791	1381.022	7034.525	20,801.23	23,724.72	13,550.53	17,865.5	11,709.24	7013.944	58,378.41
	best	1300.267	1313.111	3973.009	557,392.2	1369.765	3401.616	8156.543	1416.265	1737.605	16,864.65	9814.234	2458.308	9078.56
	worst	1307.311	1374.61	12,181.24	38,425,092	1393.283	9616.915	33,543.16	32,645.07	29,200.54	20,310.85	13,162.4	17,853.43	193,258.2
	std	3.216697	29.23646	3392.984	17,876,504	9.930585	3048.989	11,159.08	14,913.04	12,554.77	1645.737	1390.591	7306.492	89,972.56
	median	1306.198	1318.252	7996.789	9,648,339	1380.521	7559.784	20,752.61	30,418.78	11,631.99	17,143.25	11,930.17	3872.02	15,588.43
	rank	1	2	6	13	3	5	10	11	8	9	7	4	12
C17-F14	mean	1402.488	1429.937	2267.222	4189.987	1458.09	2493.258	2004.287	1444.479	2191.108	1605.133	6961.758	3115.468	13,829.69
	best	1400.997	1422.745	1465.467	1759.231	1451.345	1483.963	1540.274	1436.906	1505.748	1524.797	4027.829	1434.97	3901.489
	worst	1404.975	1447.049	4113.275	5019.092	1467.112	5421.258	2633.201	1452.614	4188.18	1637.676	9408.754	7252.678	27,663.3
	std	1.722924	11.45666	1238.764	1620.565	7.945331	1952.273	456.8535	8.481639	1331.488	53.81531	2846.787	2780.418	10,065.91
	median	1401.99	1424.977	1745.074	4990.812	1456.951	1533.905	1921.836	1444.197	1535.253	1629.029	7205.224	1887.112	11,876.98
	rank	1	2	8	11	4	9	6	3	7	5	12	10	13
C17-F15	mean	1511.413	1528.001	6200.738	11,809.76	1500.735	8083.926	10,091.39	3322.012	8029.693	1724.884	22,527.21	9559.728	4778.883
	best	1508.477	1501.587	2569.987	7105.858	1500.42	1606.064	2309.799	1533.502	1610.261	1590.433	9704.224	2974.495	1919.88
	worst	1514.151	1561.085	10,787.13	18,686.75	1501.47	23,704.43	18,933.6	6377.536	13,679.32	1821.273	31,769.43	15,791.91	8502.898
	std	2.335106	28.10815	3584.793	5429.859	0.492915	10,485.67	6814.4	2309.765	5227.34	113.2876	10,706.11	5356.91	3272.896
	median	1511.512	1524.667	5722.919	10,723.22	1500.525	3512.605	9561.076	2688.506	8414.598	1743.915	24,317.6	9736.253	4346.377
	rank	2	3	7	12	1	9	11	5	8	4	13	10	6
C17-F16	mean	1601.491	1633.587	1803.613	2096.509	1659.379	1883.829	1801.984	1935.052	1740.482	1682.598	2148.203	1947.807	1817.44
	best	1600.891	1602.605	1729.091	1912.598	1646.361	1687.047	1645.957	1843.116	1659.908	1654.683	1982.965	1839.08	1727.547
	worst	1602.221	1723.653	1908.663	2254.739	1673.281	2182.963	1909.181	2069.264	1867.991	1740.941	2270.276	2119.513	1850.824
	std	0.559227	60.05047	88.07985	173.5591	14.04957	223.369	126.0866	110.7665	95.72114	40.19961	120.5145	129.9046	59.98088
	median	1601.426	1604.046	1788.349	2109.349	1658.936	1832.652	1826.399	1913.913	1717.016	1667.383	2169.786	1916.318	1845.695
	rank	1	2	7	12	3	9	6	10	5	4	13	11	8
C17-F17	mean	1736.831	1747.593	1805.19	1874.792	1723.586	1869.789	1847.103	1780.828	1743.952	1762.775	1827.495	1756.302	1760.194
	best	1730.528	1725.836	1771.453	1802.824	1720.806	1768.912	1768.602	1731.348	1730.034	1751.879	1749.789	1749.136	1756.835
	worst	1740.08	1759.723	1868.404	1931.055	1726.376	2008.081	1910.392	1803.882	1754.053	1773.479	2038.379	1763.5	1762.82
	std	4.42058	14.96716	45.71854	61.71314	2.675517	108.6038	70.05953	33.45616	10.06638	10.69879	140.7927	6.142389	2.707453
	median	1738.357	1752.406	1790.452	1882.645	1723.581	1851.081	1854.708	1794.041	1745.86	1762.871	1760.905	1756.286	1760.561
	rank	2	4	9	13	1	12	11	8	3	7	10	5	6
C17-F18	mean	1800.837	1841.26	13,800.96	58,981,931	1877.88	22,558.16	11,714.7	16,999.55	28,058.53	31,504.64	6693.863	23,325.42	13,609.97
	best	1800.382	1813.308	7389.737	1,161,205	1855.98	7482.826	4795.665	3018.931	6287.974	25,593.54	2757.193	2958.931	3554.508
	worst	1801.23	1866.786	30,487.91	2.29 × 10^8^	1925.128	38,281.51	18,261.51	28,200.17	43,428.11	39,435.71	11,618.87	43,550.36	19,687.74
	std	0.425238	23.52028	11,179.23	1.13 × 10^8^	32.02221	16,834.36	5938.542	10,954.3	16,105.16	6367.121	3682.255	20,955.05	7046.641
	median	1800.869	1842.473	8663.093	2,922,614	1865.207	22,234.15	11,900.81	18,389.54	31,259.02	30,494.65	6199.696	23,396.19	15,598.82
	rank	1	2	7	13	3	9	5	8	11	12	4	10	6
C17-F19	mean	1900.699	1907.221	17,358.96	878,425.8	1988.542	69,005.95	90,424.78	2040.642	9820.11	4898.742	36,643.02	26,610.54	6492.073
	best	1900.02	1901.521	11,828.97	259,265.4	1939.983	1970.087	2408.219	1915.575	1928.924	2053.504	19,411.47	2676.945	2235.907
	worst	1901.018	1917.582	22,721.81	1,443,245	2029.189	26,983.7	299,607.6	2164.619	14,822.13	13,254.21	55,613.04	82,298.3	10,459.09
	std	0.469791	7.145903	4482.402	563,481.2	37.74168	131,992.5	140,090.8	137.2124	5786.879	5570.514	16,664.04	37,542.78	3392.754
	median	1900.878	1904.891	17,442.54	905,596.3	1992.498	3535.02	29,841.65	2041.187	11,264.69	2143.627	35,773.79	10,733.45	6636.648
	rank	1	2	8	13	3	11	12	4	7	5	10	9	6
C17-F20	mean	2026.662	2034.642	2147.227	2292.086	2012.062	2331.222	2203.439	2070.82	2063.92	2077.176	2275.049	2181.152	2053.815
	best	2013.865	2021.307	2076.582	2232.916	2000.995	2226.58	2194.177	2040.177	2040.405	2065.354	2190.026	2155.286	2038.374
	worst	2038.307	2044.877	2266.378	2360.461	2022.277	2500.44	2222.75	2152.489	2101.134	2088.349	2394.247	2215.298	2062.141
	std	13.07228	11.03511	84.87342	54.33571	10.64651	129.4526	13.04085	54.64582	26.46466	9.641058	98.96337	29.82688	10.95787
	median	2027.237	2036.192	2122.974	2287.484	2012.488	2298.934	2198.414	2045.306	2057.07	2077.501	2257.962	2177.013	2057.373
	rank	2	3	8	12	1	13	10	6	5	7	11	9	4
C17-F21	mean	2230.958	2255.077	2281.079	2353.337	2300.981	2301.662	2295.278	2291.145	2317.044	2306.962	2362.034	2327.466	2305.331
	best	2200	2201.126	2203.848	2268.27	2276.646	2206.365	2238.668	2200.011	2305.236	2203.991	2342.354	2318.817	2228.495
	worst	2323.833	2308.936	2367.125	2393.638	2319.224	2398.948	2357.396	2332.814	2323.9	2348.473	2373.923	2335.569	2342.504
	std	61.91636	60.47791	89.24471	57.35467	18.20708	102.8811	62.9355	61.28073	8.151	69.14021	13.6687	8.239775	51.87787
	median	2200	2255.124	2276.672	2375.72	2304.028	2300.667	2292.523	2315.878	2319.52	2337.692	2365.929	2327.739	2325.162
	rank	1	2	3	12	6	7	5	4	10	9	13	11	8
C17-F22	mean	2266.732	2309.061	2307.691	3057.272	2301.898	2714.923	2319.448	2304.337	2311.086	2321.01	2361.062	2314.243	2319.245
	best	2225.162	2304.818	2302.728	2789.931	2300.346	2334.84	2312.699	2303.309	2301.875	2314.271	2300	2300.685	2316.136
	worst	2300	2313.926	2319.708	3303.004	2306.031	3150.748	2327.601	2305.381	2325.309	2333.618	2445.235	2348.82	2324.026
	std	39.00767	3.810058	8.080836	227.6039	2.763264	410.991	6.415496	0.846824	11.19175	8.848757	72.99001	23.1002	3.37026
	median	2270.884	2308.75	2304.165	3068.076	2300.607	2687.051	2318.745	2304.328	2308.58	2318.076	2349.508	2303.733	2318.408
	rank	1	5	4	13	2	12	9	3	6	10	11	7	8
C17-F23	mean	2573.243	2573.12	2641.556	2691.514	2634.124	2671.267	2667.856	2655.194	2619.849	2645.742	2758.891	2647.616	2660.372
	best	2300.003	2377.404	2624.533	2680.076	2609.415	2628.779	2650.955	2614.194	2610.113	2634.093	2688.971	2639.73	2638.96
	worst	2776.963	2646.48	2672.977	2714.233	2699.371	2720.796	2687.563	2759.569	2626.772	2655.608	2923.019	2660.513	2669.42
	std	198.8117	130.6378	21.45668	15.59204	43.6031	40.64644	15.13134	69.72568	8.312072	9.556135	109.9465	9.372923	14.49226
	median	2608.003	2634.299	2634.356	2685.874	2613.855	2667.747	2666.454	2623.507	2621.255	2646.634	2711.786	2645.111	2666.553
	rank	2	1	5	12	4	11	10	8	3	6	13	7	9
C17-F24	mean	2517.265	2628.48	2782.755	2853.722	2500	2779.188	2796.677	2743.409	2751.65	2765.308	2582.671	2775.772	2730.119
	best	2513.188	2500.074	2762.559	2835.68	2500	2643.075	2767.067	2739.998	2734.241	2760.859	2500	2757.415	2523.272
	worst	2520.307	2751.903	2814.627	2869.479	2500	2870.335	2829	2752.217	2778.471	2769.47	2830.685	2790.675	2817.267
	std	2.967858	140.9207	22.81526	15.09578	0.000208	97.28497	25.56641	5.900329	19.28029	3.526871	165.3426	14.04169	138.498
	median	2517.783	2630.972	2776.916	2854.864	2500	2801.671	2795.32	2740.711	2746.944	2765.451	2500	2777.5	2789.97
	rank	2	4	11	13	1	10	12	6	7	8	3	9	5
C17-F25	mean	2862.423	2910.104	2973.2	3346.147	2980.623	3053.727	2933.158	2921.475	2938.295	2933.596	2932.066	2922.641	2953.708
	best	2756.464	2897.743	2948.764	3256.737	2897.743	2949.278	2907.26	2897.897	2913.367	2913.96	2897.94	2898.714	2941.202
	worst	2897.743	2945.28	3024.363	3420.107	3093.93	3308.727	2957.932	2946.111	2947.324	2952.974	2943.456	2946.61	2964.225
	std	70.63967	23.4674	35.52094	69.42892	82.11035	171.5326	28.38164	27.07956	16.62652	20.74106	22.7505	27.11627	9.803424
	median	2897.743	2898.696	2959.837	3353.872	2965.409	2978.451	2933.72	2920.945	2946.245	2933.724	2943.434	2922.619	2954.702
	rank	1	2	10	13	11	12	6	3	8	7	5	4	9
C17-F26	mean	2754.982	2943.073	3351.736	4051.78	2825.003	3869.985	4044.972	3225.067	3169.235	3229.716	3254.197	2904.365	2947.415
	best	2692.913	2800.885	3053.663	3728.9	2800.002	3472.705	3115.481	2900.125	2900.207	2912.962	2800	2800	2773.079
	worst	2800	3133.611	4095.261	4287.423	2900	4259.634	4697.221	4199.868	3803.627	3949.226	4356.049	3017.461	3125.39
	std	53.81419	167.603	500.4959	235.0147	49.99818	428.5773	676.5947	649.8669	424.9744	482.8277	738.0429	88.92102	164.2531
	median	2763.508	2918.898	3129.009	4095.399	2800.005	3873.799	4183.593	2900.139	2986.552	3028.338	2930.37	2900	2945.596
	rank	1	4	10	13	2	11	12	7	6	8	9	3	5
C17-F27	mean	3095.651	3155.778	3100.402	3198.815	3089.302	3181.938	3139.425	3092.936	3096.247	3117.018	3252.796	3139.581	3165.314
	best	3093.138	3107.813	3094.856	3132.671	3088.978	3153.813	3092.968	3089.738	3092.809	3095.827	3241.634	3097.666	3121.59
	worst	3096.979	3188.745	3104.844	3327.562	3089.706	3211.238	3246.443	3095.297	3103.359	3177.422	3260.285	3190.464	3228.683
	std	1.711133	34.28873	5.10199	87.45869	0.366278	27.96866	71.87095	2.364432	4.918617	40.27925	8.221604	39.02481	45.27772
	median	3096.244	3163.277	3100.954	3167.514	3089.262	3181.35	3109.144	3093.354	3094.41	3097.412	3254.632	3135.097	3155.491
	rank	3	9	5	12	1	11	7	2	4	6	13	8	10
C17-F28	mean	3113.935	3177.694	3259.269	3768.182	3100	3451.178	3368.732	3199.942	3410.869	3341.745	3473.798	3320.885	3257.183
	best	3100.15	3100.001	3100	3605.245	3100	3217.585	3174.627	3100.127	3383.764	3222.407	3413.862	3182.82	3148.201
	worst	3130.273	3217.332	3411.822	4044.617	3100	3652.941	3475.963	3383.75	3434.275	3412.081	3510.006	3412.053	3543.984
	std	16.04256	54.9365	130.9643	198.0034	7.84 × 10^−5^	178.7025	132.8519	134.107	20.73145	90.53327	42.51919	103.9299	191.9303
	median	3112.659	3196.722	3262.628	3711.433	3100	3467.092	3412.168	3157.946	3412.719	3366.247	3485.663	3344.334	3168.273
	rank	2	3	6	13	1	11	9	4	10	8	12	7	5
C17-F29	mean	3144.189	3217.825	3295.537	3338.434	3236.274	3303.244	3368.142	3262.597	3176.277	3218.734	3328.987	3276.182	3245.186
	best	3136.956	3165.889	3189.716	3274.636	3152.918	3217.457	3258.78	3200.595	3160.275	3168.356	3236.83	3170.757	3192.605
	worst	3153.72	3349.479	3456.253	3373.345	3303.413	3456.374	3507.331	3310.243	3196.453	3242.962	3525.19	3365.159	3298.151
	std	7.730902	87.95734	124.8115	43.68494	64.01218	108.1025	103.1848	51.8816	15.04045	35.00934	132.3611	88.34778	44.37193
	median	3143.039	3177.967	3268.089	3352.879	3244.383	3269.572	3353.228	3269.775	3174.189	3231.809	3276.964	3284.406	3244.995
	rank	1	3	9	12	5	10	13	7	2	4	11	8	6
C17-F30	mean	3422.278	9969.272	284,851.7	11,811,886	3399.757	803,656.6	1,269,588	390,395.5	778,432.3	64,677.52	925,304.9	414,393.2	1,635,020
	best	3418.586	4191.371	30,391.26	1,919,018	3395.483	20,061.19	228,475.9	15,899.26	6287.419	31,122.82	570,711.4	6603.207	562,734.7
	worst	3426.636	23,642.72	677,453.5	30,929,943	3406.359	1,678,417	3,019,728	1,479,145	2,878,412	108,709.8	1,272,741	821,874.7	3,725,142
	std	3.707695	9240.159	313,955.1	13,044,105	4.745776	901,821.7	1,308,347	726,003.4	1,402,476	37,893.81	286,632.7	469,896.9	1,490,657
	median	3421.945	6021.499	215,781	7,199,292	3398.593	758,074.1	915,074.1	33,268.63	114,515	59,438.72	928,883.5	414,547.5	1,126,102
	rank	2	3	5	13	1	9	11	6	8	4	10	7	12
Sum rank		48	104	218	363	88	303	280	152	178	217	258	207	223
Mean rank		1.655172	3.586207	7.517241	12.51724	3.034483	10.44828	9.655172	5.241379	6.137931	7.482759	8.896552	7.137931	7.689655
Total rank		1	3	8	13	2	12	11	4	5	7	10	6	9

**Table 3:** Optimization results of the CEC 2017 test suite (dimension m=30).

		GAO	WSO	AVOA	RSA	MPA	TSA	WOA	MVO	GWO	TLBO	GSA	PSO	GA
C17-F1	mean	2965.641	5.61 × 10^9^	96,586.16	4.35 × 10^10^	86,759	1.96 × 10^10^	1.67 × 10^9^	505,100.8	1.3 × 10^9^	5.71 × 10^9^	32,552,559	4.2 × 10^8^	1.92 × 10^8^
	best	105.4059	1.63 × 10^9^	3775.707	3.92 × 10^10^	7863.15	1.85 × 10^10^	9.03 × 10^8^	347,301.5	5.83 × 10^8^	3.66 × 10^9^	104.8029	6710.215	1.56 × 10^8^
	worst	6756.63	8.1 × 10^9^	363,121.4	4.78 × 10^10^	217,079.1	2.11 × 10^10^	2.61 × 10^9^	809,113.2	1.73 × 10^9^	1.01 × 10^10^	1.3 × 10^8^	1.03 × 10^9^	2.35 × 10^8^
	std	2967.156	2.79 × 10^9^	177,756.2	3.59 × 10^9^	99,555.51	1.16 × 10^9^	7.07 × 10^8^	208,219	5.09 × 10^8^	2.94 × 10^9^	65,065,475	5.08 × 10^8^	33,433,720
	median	2500.265	6.36 × 10^9^	9723.769	4.34 × 10^10^	61,046.89	1.95 × 10^10^	1.59 × 10^9^	431,994.3	1.45 × 10^9^	4.55 × 10^9^	29,684.6	3.26 × 10^8^	1.89 × 10^8^
	rank	1	10	3	13	2	12	9	4	8	11	5	7	6
C17-F3	mean	742.8731	57,920.14	52,951.58	77,211.19	789.4722	62,088.78	210,060.4	1624.497	65,596.78	35,343.56	103,615.2	44,565.91	149,306.7
	best	513.4013	48,314.06	42,383.07	70,955.93	540.9163	44,706.74	154,454.5	726.4012	56,524.54	28,173.29	98,570.69	15,600.4	111,040.5
	worst	1141.321	81,363.34	63,002.48	82,275.53	1214.161	73,753.25	254,369.3	2486.051	79,838.62	44,107.98	108,512.9	88,268.11	182,709.5
	std	289.4161	15,759.39	8486.713	4692.112	309.0768	12,486.41	48,983.66	787.5179	10,425.3	7060.413	4665.24	32,704.73	37,175.16
	median	658.3851	51,001.57	53,210.38	77,806.65	701.4055	64,947.56	215,709	1642.768	63,011.97	34,546.48	103,688.7	37,197.58	151,738.5
	rank	1	7	6	10	2	8	13	3	9	4	11	5	12
C17-F4	mean	491.1351	837.3914	534.7466	13,336.8	509.4025	3117.836	848.5098	503.0084	661.7039	748.9401	624.7152	675.6909	735.2118
	best	468.6344	725.9771	521.2662	7325.298	486.7772	868.6846	745.6112	488.4792	538.4455	668.2051	567.1673	499.2964	707.5575
	worst	512.6268	1058.681	549.2611	26,000.81	542.6027	5006.098	936.1373	517.528	754.3157	849.7198	752.0121	1076.728	761.2708
	std	18.01092	154.883	12.25453	8716.748	25.44004	1808.51	95.9796	15.12204	92.02884	80.56382	86.61078	271.7502	26.1284
	median	491.6396	782.4537	534.2294	10,010.54	504.115	3298.28	856.1454	503.0133	677.0271	738.9177	589.8407	563.3699	736.0095
	rank	1	10	4	13	3	12	11	2	6	9	5	7	8
C17-F5	mean	585.6623	644.6729	762.3586	917.5582	604.7663	884.8544	838.1558	594.886	619.214	731.8464	717.6456	674.1303	706.7469
	best	582.592	616.8786	735.8031	894.1469	594.6117	796.6634	807.3832	579.0498	588.317	719.8893	702.9702	660.1907	686.0723
	worst	591.1983	687.2621	786.545	948.5521	629.3993	1049.042	900.7655	631.5963	646.9154	747.028	727.844	685.1118	720.6189
	std	4.041704	31.89212	22.97672	23.33033	16.49975	112.9375	43.3928	24.77706	26.59695	12.88375	11.92192	11.58915	14.84429
	median	584.4295	637.2754	763.5432	913.7669	597.527	846.8561	822.2373	584.4489	620.8117	730.2343	719.8841	675.6094	710.1482
	rank	1	5	10	13	3	12	11	2	4	9	8	6	7
C17-F6	mean	606.7204	637.6933	657.7141	685.7894	602.6388	682.6748	681.8045	622.9356	606.9295	643.482	658.2771	647.6491	631.1395
	best	605.1486	633.5361	641.1756	679.3235	600.8551	671.0576	669.5708	615.361	604.7665	632.1329	653.7776	637.6309	630.2327
	worst	607.4042	644.45	667.264	688.9118	603.6152	695.4225	697.3843	628.5635	608.055	659.2047	662.2351	661.9608	632.1984
	std	1.05629	4.867309	11.70441	4.513135	1.261537	10.19072	12.05372	5.52481	1.476224	11.71106	3.483087	10.33603	0.976799
	median	607.1643	636.3935	661.2084	687.4612	603.0425	682.1095	680.1315	623.9089	607.4483	641.2951	658.5478	645.5023	631.0635
	rank	2	6	9	13	1	12	11	4	3	7	10	8	5
C17-F7	mean	820.8024	1089.043	1182.495	1395.582	854.1701	1325.59	1270.217	845.7349	881.1955	1068.159	949.8174	930.2214	984.2242
	best	813.7177	995.5985	1059.063	1322.22	835.6078	1266.912	1240.955	833.2816	854.9027	1041.927	922.6795	868.6313	961.3759
	worst	827.619	1184.217	1248.513	1438.018	869.8823	1349.314	1329.113	860.5127	902.7389	1092.014	976.0323	992.083	1011.485
	std	6.229666	77.96311	88.58013	50.57298	17.30967	39.38619	40.02886	13.81518	19.79133	26.48992	26.05838	50.43402	23.39094
	median	820.9365	1088.178	1211.201	1411.044	855.5951	1343.067	1255.4	844.5726	883.5701	1069.347	950.2788	930.0856	982.0181
	rank	1	9	10	13	3	12	11	2	4	8	6	5	7
C17-F8	mean	877.6456	916.3291	978.0973	1133.102	901.0738	1140.52	1017.573	902.8113	902.7396	1038.544	966.9033	924.7556	993.9056
	best	866.6767	887.4457	936.3094	1125.885	884.5957	1053.955	979.1085	863.1024	872.8583	1023.917	946.2582	887.5568	959.312
	worst	892.3077	942.7474	1015.905	1146.021	911.7185	1205.168	1089.695	917.7493	928.7814	1053.247	1004.96	976.1195	1021.463
	std	12.03403	24.70294	32.73762	8.879199	11.97364	64.32434	49.15648	26.50395	29.49711	12.9849	27.48129	43.15555	26.37234
	median	875.7989	917.5616	980.0876	1130.251	903.9904	1151.478	1000.745	915.1968	904.6592	1038.506	958.1975	917.6731	997.4234
	rank	1	5	8	12	2	13	10	4	3	11	7	6	9
C17-F9	mean	1165.65	6118.317	5285.631	10,677.51	1310.315	13,059.8	7819.018	6584.038	2283.474	4651.679	4123.625	3535.136	1379.814
	best	1048.058	3812.091	4583.34	8370.654	1103.222	8931.468	6525.942	1125.644	1241.751	3242.499	3402.813	2404.144	1225.076
	worst	1337.301	7705.346	5737.959	12,502.83	1788.264	19,283.94	9256.488	11,627.78	3578.942	5837.91	4747.383	5875.287	1543.585
	std	130.0098	1697.137	556.4414	1715.525	325.1294	4428.538	1255.761	4991.033	982.1215	1196.281	613.4941	1593.562	137.1979
	median	1138.621	6477.916	5410.614	10,918.27	1174.887	12,011.89	7746.822	6791.365	2156.601	4763.153	4172.152	2930.556	1375.297
	rank	1	9	8	12	2	13	11	10	4	7	6	5	3
C17-F10	mean	3686.42	5097.435	5376.248	8165.39	3966.598	7339.579	7588.39	5284.051	4409.938	8329.399	4799.841	4968.059	6555.299
	best	3486.984	4046.474	5210.865	8063.814	3600.688	6817.31	6285.025	4499.451	4009.121	8216.554	4150.098	4099.397	5554.57
	worst	3851.9	7007.736	5672.714	8257.688	4371.786	7861.61	8551.306	6421.91	5039.747	8519.895	5515.315	5511.443	7161.94
	std	189.092	1365.454	209.6517	80.84512	346.6865	426.3938	989.7159	811.0246	458.9879	131.8935	622.5674	670.3383	706.2351
	median	3703.398	4667.766	5310.706	8170.029	3946.959	7339.699	7758.615	5107.422	4295.441	8290.573	4766.976	5130.698	6752.344
	rank	1	6	8	12	2	10	11	7	3	13	4	5	9
C17-F11	mean	1176.86	1510.898	1316.714	7335.945	1209.9	3557.005	7893.704	1361.737	1683.239	1916.548	3506.789	1349.558	5022.123
	best	1156.088	1422.613	1266.473	6658.404	1160.183	2246.748	3014.369	1289.335	1556.019	1726.753	3036.57	1298.049	3707.247
	worst	1200.911	1658.534	1437.228	8165.895	1237.557	4990.051	12,541.55	1445.222	1913.274	2124.247	4371.403	1394.515	7220.175
	std	18.56114	109.8245	81.3137	622.5615	34.54917	1165.235	3987.813	71.07825	158.4847	166.7819	629.2975	51.30956	1525.397
	median	1175.22	1481.223	1281.577	7259.74	1220.931	3495.611	8009.447	1356.196	1631.832	1907.597	3309.592	1352.835	4580.536
	rank	1	6	3	12	2	10	13	5	7	8	9	4	11
C17-F12	mean	85,555.87	25,949,230	10,375,592	1.32 × 10^10^	89,184.91	6.12 × 10^9^	3.34 × 10^8^	28,219,307	1.35 × 10^8^	3.08 × 10^8^	84,031,918	62,898,515	8,506,616
	best	25,576.26	1,213,875	1,853,642	9.56 × 10^9^	26,351.25	4.1 × 10^9^	44,146,877	9,447,872	77,445,909	1.76 × 10^8^	3,464,275	201,962.8	3,266,660
	worst	191,112.3	73,085,854	30,139,971	1.66 × 10^10^	200,262	8.4 × 10^9^	8.95 × 10^8^	71,379,451	2.74 × 10^8^	4.53 × 10^8^	1.6 × 10^8^	2 × 10^8^	12,986,786
	std	72,522.38	32,367,345	13,248,399	2.96 × 10^9^	76,266.98	2.02 × 10^9^	3.84 × 10^8^	29,109,113	93,894,353	1.37 × 10^8^	77,920,027	94,482,882	5,007,932
	median	62,767.48	14,748,596	4,754,377	1.34 × 10^10^	65,063.21	5.98 × 10^9^	1.99 × 10^8^	16,024,953	93,914,719	3.01 × 10^8^	86,487,233	25,671,885	8,886,510
	rank	1	5	4	13	2	12	11	6	9	10	8	7	3
C17-F13	mean	1679.051	65,307.57	196,312.1	9.71 × 10^9^	1663.41	1.62 × 10^9^	1,218,387	169,962.5	107,891.8	1.05 × 10^8^	34,883.97	1,140,606	9,001,402
	best	1509.439	10,167.41	64,177.33	5.02 × 10^9^	1496.899	9,775,852	190,877.6	61,271.86	80,912.23	73,593,614	29,396.19	27,575.57	2,238,017
	worst	1846.767	222,691.8	385,695.2	1.73 × 10^10^	1828.612	4.01 × 10^9^	3,666,218	384,298.9	157,114.6	1.61 × 10^8^	47,660	4,444,295	13,874,918
	std	138.2768	104,969.9	146,282.3	5.43 × 10^9^	136.0205	1.9 × 10^9^	1,643,068	146,086.5	34,141.59	38,514,757	8575.928	2,202,480	5,734,672
	median	1679.999	14,185.55	167,687.9	8.27 × 10^9^	1664.065	1.23 × 10^9^	508,225.6	117,139.5	96,770.14	92,927,829	31,239.85	45,277.58	9,946,336
	rank	2	4	7	13	1	12	9	6	5	11	3	8	10
C17-F14	mean	1445.686	5029.534	310,318	3,166,786	1506.721	944,929.1	1,449,403	8360.852	312,592.2	104,927.4	1,488,236	17,259.01	2,471,053
	best	1439.939	1874.271	190,232.1	1,938,929	1490.417	43,337.9	831,823.4	5657.736	44,267.26	55,063.1	589,278.8	5235.579	1,453,463
	worst	1458.422	13,681.86	385,275.7	5,133,496	1521.294	1,897,719	1,916,237	11,991.96	1,004,397	156,646	2,344,763	25,836.68	3,521,220
	std	8.727275	5779.772	87,907.64	1,445,810	13.57225	1,040,334	532,404.3	3010.837	464,006.7	57,133.45	881,268.4	9220.18	1,034,268
	median	1442.191	2281.001	332,882.1	2,797,359	1507.587	919,329.9	1,524,776	7896.855	100,852.1	104,000.2	1,509,451	18,981.88	2,454,764
	rank	1	3	7	13	2	9	10	4	8	6	11	5	12
C17-F15	mean	1588.785	2511.297	45,133.53	3.52 × 10^8^	1577.262	2.66 × 10^8^	1,282,549	56,902.87	629,660.2	2,881,979	18,208.94	8283.199	986,862.4
	best	1560.073	1925.339	26,535.07	2.27 × 10^8^	1550.94	206,063.4	348,434.1	38,106.18	21,681.95	1,030,920	8605.33	2205.324	367,843.7
	worst	1624.03	3215.395	59,058.66	5.16 × 10^8^	1612.701	1.03 × 10^9^	2,123,573	96,468.19	2,007,235	4,849,628	22,396.17	15,491.71	1,359,201
	std	26.94309	601.986	14,789.58	1.28 × 10^8^	26.55107	5.06 × 10^8^	948,175.3	27,305.97	939,116.3	1,581,134	6440.914	5566.237	447,634.1
	median	1585.518	2452.228	47,470.2	3.32 × 10^8^	1572.705	19,428,469	1,329,094	46,518.56	244,862	2,823,685	20,917.14	7717.879	1,110,203
	rank	2	3	6	13	1	12	10	7	8	11	5	4	9
C17-F16	mean	2157.589	2621.237	3504.832	5684.392	2411.321	3393.39	4006.918	2640.575	2419.743	3554.599	3382.484	2628.98	3048.951
	best	2037.231	2230.767	3285.167	4800.654	2317.615	2965.308	3410.842	2505.972	2097.616	3255.021	3093.147	2304.9	2816.301
	worst	2228.757	2887.896	3750.51	7790.203	2527.256	3870.972	4848.864	2829.698	2713.406	4153.135	3550.905	3171.968	3285.631
	std	87.98998	277.6318	202.3443	1409.831	88.86949	388.2075	675.2394	145.1525	262.9818	420.4846	201.6026	375.8814	209.7332
	median	2182.184	2683.142	3491.826	5073.356	2400.206	3368.641	3883.982	2613.315	2433.974	3405.121	3442.941	2519.526	3046.937
	rank	1	4	10	13	2	9	12	6	3	11	8	5	7
C17-F17	mean	1871.456	2005.135	2453.821	10,597.65	1909.902	2475.363	2640.065	2224.961	2019.268	2264.054	2517.678	2212.343	2210.214
	best	1831.18	1929.821	2053.719	3574.957	1846.274	2095.324	2481.288	1977.931	1914.84	2076.245	2318.989	1853.445	2079.712
	worst	1906.218	2165.417	2812.059	31,274.61	1967.102	2698.859	2719.99	2613.998	2144.187	2524.232	2753.058	2559.711	2400.234
	std	34.6833	108.3281	340.2979	13,785.77	50.24343	276.5611	108.6442	275.7001	97.66128	187.9479	216.4953	294.7102	143.1001
	median	1874.214	1962.652	2474.753	3770.522	1913.116	2553.635	2679.491	2153.958	2009.023	2227.87	2499.333	2218.109	2180.455
	rank	1	3	9	13	2	10	12	7	4	8	11	6	5
C17-F18	mean	1884.562	90,919.87	1,389,568	80,080,025	1950.484	2,428,856	8,909,556	517,529.6	1,541,680	3,526,422	240,227.7	198,781.2	5,054,893
	best	1857.253	26,791.6	299,648	52,474,251	1905.935	122,389.8	852,143	118,784.1	100,638.4	1,433,012	99,249.04	68,546.56	1,605,174
	worst	1941.743	266,184.6	3,384,215	99,570,904	1995.318	5,934,470	21,737,586	984,081.8	3,883,479	6,498,024	404,021.8	406,261.2	7,996,236
	std	38.65561	117,099.7	1,452,873	20,359,139	39.19088	2,482,090	9,892,121	367,838.4	1,668,754	2,264,944	137,125	149,305.7	3,298,551
	median	1869.625	35,351.63	937,204.9	84,137,472	1950.342	1,829,282	6,524,247	483,626.2	1,091,301	3,087,327	228,820.1	160,158.4	5,309,081
	rank	1	3	7	13	2	9	12	6	8	10	5	4	11
C17-F19	mean	1950.499	3335.472	91,572.27	6.82 × 10^8^	1933.262	1.54 × 10^9^	14,619,971	1,421,627	276,846.2	5,881,714	160,121	14,630.44	485,098.8
	best	1940.669	2030.43	14,294.76	4.52 × 10^8^	1921.712	41,342,458	5,450,995	72,186	73,307.97	4,590,769	121,887.8	8707.597	171,700.8
	worst	1958.422	5624.54	144,128.7	8.89 × 10^8^	1939.359	5.66 × 10^9^	32,121,702	2,690,213	490,870.3	7,719,442	215,531.7	18,279.15	847,776
	std	7.641626	1581.365	55,588.55	2.04 × 10^8^	7.885053	2.75 × 10^9^	12,527,996	1,129,134	170,582.7	1,476,862	44,979.38	4145.322	295,341.7
	median	1951.452	2843.459	103,932.8	6.94 × 10^8^	1935.989	2.35 × 10^8^	10,453,594	1,462,055	271,603.3	5,608,322	151,532.2	15,767.5	460,459.2
	rank	2	3	5	12	1	13	11	9	7	10	6	4	8
C17-F20	mean	2218.661	2321.351	2605.831	3021.328	2217.587	2692.641	3100.685	2494.312	2542.181	2581.204	3143.322	2630.276	2434.181
	best	2172.115	2237.079	2338.79	2931.969	2153.46	2543.643	3041.176	2281.39	2348.435	2429.147	2904.297	2303.65	2279.759
	worst	2243.554	2411.444	2769.648	3102.604	2258.994	2862.524	3229.713	2750.27	2743.214	2770.632	3264.54	3049.42	2518.498
	std	31.80584	86.94046	185.8798	72.53707	47.02587	131.2394	87.90274	219.2801	170.086	148.2222	167.9671	309.2781	106.8744
	median	2229.487	2318.44	2657.442	3025.37	2228.948	2682.199	3065.925	2472.794	2538.537	2562.518	3202.225	2584.017	2469.234
	rank	2	3	8	11	1	10	12	5	6	7	13	9	4
C17-F21	mean	2403.602	2455.2	2537.576	2671.54	2451.798	2596.489	2624.108	2464.941	2410.178	2548.284	2616.671	2454.839	2547.871
	best	2355.743	2413.064	2515.265	2616.289	2381.612	2592.025	2590.862	2410.388	2368.967	2531.453	2609.737	2435.622	2516.184
	worst	2519.24	2491.044	2565.94	2732.226	2527.027	2603.754	2654.393	2581.813	2499.48	2569.234	2620.676	2473.227	2568.211
	std	77.40211	32.04248	25.48828	50.22592	66.98331	5.380629	34.4848	80.83535	60.28216	15.62456	5.011353	20.69181	22.45604
	median	2369.713	2458.347	2534.549	2668.822	2449.276	2595.088	2625.589	2433.781	2386.132	2546.225	2618.136	2455.253	2553.544
	rank	1	5	7	13	3	10	12	6	2	9	11	4	8
C17-F22	mean	2300.912	3420.821	6016.585	8171.962	2435.215	9572.129	8317.425	4285.503	5403.623	5171.095	7079.976	6949.861	2764.351
	best	2300.607	2826.629	2312.315	7003.963	2423.944	9378.809	8097.021	2307.191	2612.314	3144.675	6045.552	5921.042	2717.751
	worst	2301.75	4314.511	7897.47	9708.421	2442.782	9901.309	8511.15	6555.56	7165.865	10,311.14	7937.065	7465.019	2810.387
	std	0.559452	694.6689	2514.73	1125.176	7.982382	241.5606	169.8963	2295.564	1953.817	3437.178	779.0079	696.902	43.96047
	median	2300.646	3271.073	6928.279	7987.733	2437.066	9504.198	8330.764	4139.63	5918.157	3614.284	7168.643	7206.692	2764.634
	rank	1	4	8	11	2	13	12	5	7	6	10	9	3
C17-F23	mean	2730.471	3215.293	2991.025	3298.124	2774.497	3300.559	3153.664	2764.772	2761.243	2948.592	3884.844	3000.973	2999.073
	best	2691.157	3096.518	2917.382	3122.908	2725.57	3189.956	2894.171	2719.353	2741.353	2935.222	3783.583	2871.538	2969.309
	worst	2806.588	3435.044	3070.868	3564.946	2861.898	3476.408	3302.155	2829.515	2784.476	2959.248	4063.831	3136.266	3021.883
	std	52.25212	154.119	62.77169	188.2427	62.9445	122.8799	183.8363	49.77987	21.54624	10.06741	123.1166	135.0474	22.61169
	median	2712.069	3164.806	2987.924	3252.32	2755.26	3267.937	3209.165	2755.109	2759.571	2949.949	3845.981	2998.045	3002.549
	rank	1	10	6	11	4	12	9	3	2	5	13	8	7
C17-F24	mean	2880.63	3259.635	3118.144	3406.151	2907.605	3367.598	3213.517	2920.928	2939.198	3069.087	3490.473	3079.172	3259.002
	best	2809.71	2786.217	3088.489	3350.229	2894.427	3255.152	3129.533	2904.032	2880.904	3027.837	3441.392	2972.894	3201.354
	worst	2905.164	3458.681	3144.198	3521.157	2932.457	3464.58	3330.582	2949.794	3057.925	3100.627	3538.605	3258.899	3339.613
	std	47.28496	317.1838	24.04771	77.69108	16.93679	96.09487	84.67507	20.89335	83.43905	36.60211	50.18546	132.8847	59.21531
	median	2903.824	3396.821	3119.945	3376.608	2901.769	3375.33	3196.976	2914.944	2908.981	3073.941	3490.948	3042.448	3247.52
	rank	1	10	7	12	2	11	8	3	4	5	13	6	9
C17-F25	mean	2887.905	3057.34	2938.705	5015.747	3023.956	3466.492	3091.419	2918.07	3043.638	3150.619	2979.409	2922.536	3114.78
	best	2883.986	2987.626	2901.366	4384.512	2890.06	3332.176	3007.826	2888.094	3016.157	3077.278	2952.687	2897.356	3072.749
	worst	2891.073	3119.548	2992.388	6638.173	3089.805	3555.045	3148.11	2971.717	3100.885	3309.807	3001.141	2942.863	3167.876
	std	2.933447	54.63684	40.01622	1085.805	92.49693	95.26176	59.59986	36.8016	39.61029	109.4526	25.09905	20.98515	39.36999
	median	2888.281	3061.094	2930.532	4520.151	3057.98	3489.374	3104.87	2906.234	3028.756	3107.696	2981.904	2924.962	3109.247
	rank	1	8	4	13	6	12	9	2	7	11	5	3	10
C17-F26	mean	2932.162	5792.551	7112.147	10,557.33	2983.243	8157.806	8582.206	5399.407	5149.351	6657.232	8101.595	5167.292	4370.346
	best	2887.387	4244.662	6655.78	9945.558	2900.722	7082.87	8242.061	4798.038	4796.37	6422.825	7075.436	3618.219	4134.387
	worst	3041.078	7147.491	7659.167	10,890.32	3093.717	9406.218	8827.586	5939.213	5750.536	6899.911	8712.858	6951.381	4757.879
	std	72.85753	1451.242	494.8827	426.6739	91.42468	1081.231	249.6577	480.6749	416.3707	201.3114	711.6418	1720.183	269.6611
	median	2900.091	5889.027	7066.82	10,696.71	2969.267	8071.068	8629.589	5430.189	5025.249	6653.096	8309.043	5049.785	4294.56
	rank	1	7	9	13	2	11	12	6	4	8	10	5	3
C17-F27	mean	3212.022	3534.047	3250.925	3962.572	3298.17	3496.265	3494.564	3236.161	3231.383	3307.425	5546.879	3314.279	3512.212
	best	3195.721	3352.081	3233.082	3632.976	3229.714	3407.483	3358.285	3206.726	3216.532	3261.276	5064.765	3233.265	3430.828
	worst	3222.351	3740.069	3276.898	4849.667	3327.071	3595.241	3813.546	3291.474	3237.716	3362.276	6389.948	3428.025	3576.987
	std	11.44629	170.5496	18.90387	592.3009	45.85752	89.95278	214.1498	37.69573	9.949078	41.58353	595.0611	83.74264	66.5742
	median	3215.009	3522.018	3246.861	3683.823	3317.949	3491.168	3403.213	3223.222	3235.643	3303.073	5366.403	3297.913	3520.516
	rank	1	11	4	12	5	9	8	3	2	6	13	7	10
C17-F28	mean	3302.861	3546.371	3285.617	5932.203	3221.572	4134.473	3494.575	3246.338	3424.764	3666.029	3691.62	3314.008	3627.343
	best	3254.533	3469.684	3267.981	5550.003	3202.752	3948.554	3413.911	3220.435	3327.276	3536.347	3489.929	3218.089	3539.319
	worst	3362.259	3604.984	3309.976	6420.009	3252.13	4537.352	3626.089	3280.945	3471.122	3927.693	4071.334	3392.695	3680.818
	std	45.60338	57.61343	20.43417	429.9783	22.14656	274.6438	92.90273	29.05037	67.28895	179.3281	270.4868	85.12066	65.47424
	median	3297.327	3555.407	3282.255	5879.401	3215.704	4025.993	3469.15	3241.986	3450.329	3600.038	3602.609	3322.625	3644.617
	rank	4	8	3	13	1	12	7	2	6	10	11	5	9
C17-F29	mean	3560.528	3889.112	4479.882	5919.125	3675.267	5353.658	5306.859	4004.846	4025.698	4349.599	5280.968	4218.421	4293.605
	best	3490.151	3584.398	4313.698	4909.763	3542.422	5046.272	4630.521	3646.815	3755.698	4237.691	4911.985	3808.914	4140.599
	worst	3611.59	4163.922	4589.025	6982.043	3823.444	5681.6	5963.599	4220.558	4263.946	4591.053	5740.286	4865.097	4443.725
	std	54.88954	237.8957	118.5045	847.9831	117.1898	292.4008	561.9992	254.8365	247.9412	166.1172	389.2517	452.4459	158.5205
	median	3570.185	3904.065	4508.403	5892.347	3667.601	5343.38	5316.658	4076.006	4041.574	4284.826	5235.801	4099.837	4295.048
	rank	1	3	9	13	2	12	11	4	5	8	10	6	7
C17-F30	mean	7742.305	179,776.5	1,269,100	3.38 × 10^9^	7679.743	60,768,716	32,463,948	3,549,182	6,660,655	28,705,893	2,737,261	99,984.65	992,091.5
	best	6292.261	22,142.69	383,760.8	2.52 × 10^9^	6248.998	13,475,637	9,172,643	1,518,619	5,440,686	19,256,126	1,341,586	17,644.38	266,268.1
	worst	9407.668	459,312.2	2,509,342	4.45 × 10^9^	9322.427	1.18 × 10^8^	54,601,906	7,489,490	8,069,479	44,576,334	4,164,795	290,688.3	2,346,103
	std	1283.647	191,970.5	921,032.2	9.95 × 10^8^	1265.365	43,813,635	23,396,892	2,771,717	1,213,043	11,210,682	1,399,201	129,358	925,161.8
	median	7634.645	118,825.6	1,091,648	3.27 × 10^9^	7573.774	55,723,914	33,040,622	2,594,310	6,566,227	25,495,556	2,721,331	45,802.95	677,997.4
	rank	2	4	6	13	1	12	11	8	9	10	7	3	5
Sum rank		38	174	195	361	64	324	309	141	157	249	244	166	217
Mean rank		1.310345	6	6.724138	12.44828	2.206897	11.17241	10.65517	4.862069	5.413793	8.586207	8.413793	5.724138	7.482759
Total rank		1	6	7	13	2	12	11	3	4	10	9	5	8

**Table 4:** Optimization results of the CEC 2017 test suite (dimension m=50).

		GAO	WSO	AVOA	RSA	MPA	TSA	WOA	MVO	GWO	TLBO	GSA	PSO	GA
C17-F1	mean	14,553.5	2.52 × 10^10^	12,002,919	9.79 × 10^10^	8,328,904	4.48 × 10^10^	8.24 × 10^9^	4,657,064	1.08 × 10^10^	2.21 × 10^10^	1.79 × 10^10^	4.29 × 10^9^	7.95 × 10 ^9^
	best	11,540.48	1.57 × 10^10^	4,081,906	8.7 × 10^10^	5,232,940	2.5 × 10^10^	4.23 × 10^9^	4,298,380	4.37 × 10^9^	1.9 × 10^10^	1.41 × 10^10^	1.63 × 10^9^	4.59 × 10 ^9^
	worst	18,594.35	2.97 × 10^10^	30,750,213	1.1 × 10^11^	11,801,662	5.49 × 10^10^	1.26 × 10^10^	5,533,840	1.52 × 10^10^	3.05 × 10^10^	2.24 × 10^10^	7.79 × 10^9^	1.06 × 10 ^10^
	std	2982.205	6.44 × 10^9^	12,587,166	9.2 × 10^9^	2,866,669	1.37 × 10^10^	3.45 × 10^9^	589,793.4	4.81 × 10^9^	5.58 × 10^9^	3.67 × 10^9^	2.59 × 10^9^	2.62 × 10 ^9^
	median	14,039.59	2.77 × 10^10^	6,589,779	9.76 × 10^10^	8,140,507	4.96 × 10^10^	8.08 × 10^9^	4,398,018	1.19 × 10^10^	1.95 × 10^10^	1.76 × 10^10^	3.87 × 10^9^	8.29 × 10 ^9^
	rank	1	11	4	13	3	12	7	2	8	10	9	5	6
C17-F3	mean	17,143.34	105,001.8	165,391.3	175,765.8	17,990.03	104,172.2	256,160.7	47,195.6	132,271.4	96,821.27	198,666.9	189,249.9	295,551.5
	best	14,095.85	82,400.47	158,593.6	163,034.7	14,878.3	83,456.61	210,096.4	38,369.45	116,283.4	93,036.56	180,821	150,802.2	233,017.7
	worst	20,804.6	125,914.7	184,587.1	189,487.5	21,815.8	126,814.1	322,045.9	57,270.58	143,028.9	101,964.3	206,713.8	224,403.1	349,331.2
	std	2926.758	18,840.53	12,801.09	10,869.46	3124.825	21,210.52	48,332.24	9608.053	12,184.08	3778.635	12,005.96	33,873.53	47,753.24
	median	16,836.45	105,845.9	159,192.3	175,270.5	17,633.01	103,209.1	246,250.2	46,571.19	134,886.7	96,142.11	203,566.5	190,897.1	299,928.5
	rank	1	6	8	9	2	5	12	3	7	4	11	10	13
C17-F4	mean	555.9812	3922.348	668.4579	24,374.45	581.4708	8455.619	2572.61	568.3633	1370.46	2192.35	3087.765	1068.314	1748.455
	best	533.964	2936.023	594.6227	18,669.14	558.7435	5004.304	1750.866	541.0309	1262.635	1176.683	2613.392	672.7473	1533.777
	worst	583.575	5345.994	743.6194	26,403.46	624.1394	12,427.34	3482.656	620.8195	1468.316	4872.366	3772.885	1687.916	2035.707
	std	24.79482	1017.416	64.95445	3805.576	30.36838	3309.715	712.975	37.57762	86.09834	1789.646	561.3773	475.3013	222.7235
	median	553.193	3703.689	667.7947	26,212.6	571.5002	8195.416	2528.459	555.8013	1375.445	1360.176	2982.392	956.2969	1712.169
	rank	1	11	4	13	3	12	9	2	6	8	10	5	7
C17-F5	mean	714.1266	802.4974	901.4393	1166.905	759.3991	1220.453	1069.522	804.4484	760.2189	1044.878	846.7404	815.0165	947.1408
	best	681.6816	764.8877	884.7392	1146.007	709.5423	1156.624	1000.118	709.3516	745.8673	1019.009	830.3242	751.8603	890.8249
	worst	764.4116	869.0053	924.423	1209.405	814.5279	1274.963	1176.858	1007.618	789.3257	1089.369	879.0758	927.8653	977.0538
	std	38.65027	48.52667	17.12741	28.97798	49.22487	54.88339	81.04347	140.6692	19.80102	30.78979	21.95627	77.49311	40.3683
	median	705.2067	788.0483	898.2975	1156.103	756.7632	1225.113	1050.556	750.412	752.8413	1035.567	838.7808	790.1702	960.3423
	rank	1	4	8	12	2	13	11	5	3	10	7	6	9
C17-F6	mean	611.2095	657.5897	660.8563	698.9123	638.1769	695.1733	700.1844	638.8012	621.6137	668.3177	663.8223	650.4509	646.5533
	best	609.7563	648.602	654.2488	696.8387	626.523	684.2707	696.5667	629.1829	617.123	658.1773	657.2969	645.7214	638.6868
	worst	612.6181	671.2048	667.3298	700.4788	649.3564	703.0004	705.4916	651.6013	627.9207	678.5366	669.878	658.6062	655.6196
	std	1.225993	10.35935	5.352443	1.667333	9.503886	8.42664	4.266327	9.615536	4.92571	9.30888	5.167045	5.886562	6.951281
	median	611.2318	655.276	660.9234	699.1658	638.4141	696.711	699.3397	637.2102	620.7055	668.2784	664.0572	648.7379	645.9535
	rank	1	7	8	12	3	11	13	4	2	10	9	6	5
C17-F7	mean	1025.881	1721.176	1662.322	1979.496	1061.397	1869.978	1952.088	1122.436	1175.817	1555.17	1489.926	1294.5	1340.208
	best	1000.164	1616.331	1507.457	1926.181	1041.209	1737.332	1856.546	1041.995	1075.823	1497.521	1409.155	1193.467	1306.069
	worst	1057.179	1834.234	1814.065	2015.581	1070.832	2015.128	2040.279	1205.173	1337.112	1655.586	1569.725	1373.801	1369.909
	std	24.35707	112.7002	141.5327	40.63313	13.62912	119.6811	90.20122	66.70626	112.9076	74.73389	70.13841	77.66052	26.6831
	median	1023.091	1717.069	1663.884	1988.111	1066.772	1863.727	1955.764	1121.288	1145.167	1533.787	1490.412	1305.365	1342.426
	rank	1	10	9	13	2	11	12	3	4	8	7	5	6
C17-F8	mean	995.6338	1199.155	1187.135	1475.118	1047.464	1481.246	1287.263	1091.472	1048.355	1328.305	1167.883	1138.155	1266.072
	best	983.0873	1157.66	1104.657	1442.163	1029.584	1373.77	1255.154	1019.53	978.9894	1293.787	1142.263	1056.075	1220.748
	worst	1013.932	1243.697	1273.021	1518.174	1071.654	1617.331	1339.21	1149.662	1111.758	1373.885	1229.819	1194.697	1319.763
	std	14.65683	42.67117	70.45638	31.82365	20.59496	105.9277	37.15687	54.45485	55.44733	36.7254	41.67991	60.67326	47.71119
	median	992.7582	1197.632	1185.432	1470.067	1044.309	1466.941	1277.345	1098.349	1051.337	1322.774	1149.726	1150.925	1261.889
	rank	1	8	7	12	2	13	10	4	3	11	6	5	9
C17-F9	mean	3508.77	28,911.38	14,239.23	36,756.45	4405.262	40,698.39	27,774.69	16,769.14	9682.165	21,676.54	11,727.2	10,563.16	12,676.64
	best	2346.403	23,517.24	12,456.48	35,478.1	2454.814	25,476.68	20,652.83	6137.118	4087.611	13,214.73	9840.69	7224.243	10,662.33
	worst	4108.611	33,883.7	16,745.19	38,570.8	7193.914	49,967.28	36,542.78	25,502.92	19,344.7	24,926.32	13,118.38	15,004.49	18,314.94
	std	802.4975	5435.387	1862.83	1513.347	2006.523	10,591.15	6557.658	8003.097	6849.259	5651.353	1499.96	3310.715	3760.074
	median	3790.033	29,122.29	13,877.63	36,488.46	3986.16	43,674.8	26,951.58	17,718.27	7648.173	24,282.56	11,974.87	10,011.96	10,864.65
	rank	1	11	7	12	2	13	10	8	3	9	5	4	6
C17-F10	mean	5767.859	8659.802	8739.107	14,657.63	6478.647	12,244.83	12,459.21	8006.238	7533.535	14,827.86	8542.661	7895.046	12,313.92
	best	5366.673	6200.492	8098.813	13,640.76	5496.015	11,283.26	11,117.82	6719.282	6798.832	14,503.11	8163.447	6177.5	11,437.17
	worst	6104.604	14,286.54	9637.659	15,377.43	7166.545	12,768.73	13,353.26	9065.671	7954.246	15,217.52	9146.476	9662.261	13,740.61
	std	335.1443	3810.289	691.5653	853.5516	711.7507	657.4143	1024.224	968.6225	546.4662	334.1279	446.1412	1429.865	1006.377
	median	5800.079	7076.087	8609.978	14,806.17	6626.014	12,463.65	12,682.88	8120	7690.53	14,795.4	8430.361	7870.211	12,038.94
	rank	1	7	8	12	2	9	11	5	3	13	6	4	10
C17-F11	mean	1263.481	6096.42	1626.641	20,780.93	1282.969	11,745.2	6389.68	1437.742	5679.881	4963.278	16,289.4	2583.926	24,961.02
	best	1232.308	3844.968	1430.406	19,217.1	1246.454	5214.658	4045.917	1275.734	3552.577	3857.778	11,845.87	1564.863	14,294.87
	worst	1283.051	8972.834	1722.341	22,088.57	1308.7	19,864.68	10,059.87	1576.555	8211.372	6388.745	19,867.07	4627.567	50,045.37
	std	23.13329	2347.125	132.8201	1396.216	26.16396	6685.04	2575.006	132.8975	2117.415	1050.716	3574.724	1389.351	16,806.27
	median	1269.283	5783.94	1676.908	20,909.02	1288.361	10,950.73	5726.466	1449.339	5477.787	4803.294	16,722.33	2071.638	17,751.93
	rank	1	8	4	12	2	10	9	3	7	6	11	5	13
C17-F12	mean	3,759,630	3.08 × 10^9^	63,614,775	8.18 × 10^10^	15,933,816	2.13 × 10^10^	1.83 × 10^9^	92,770,078	4.76 × 10^8^	4.26 × 10^9^	2.06 × 10^9^	2.03 × 10^9^	1.93 × 10 ^8^
	best	3,120,877	6.07 × 10^8^	25,515,066	6.07 × 10^10^	3,269,247	1.42 × 10^10^	1.01 × 10^9^	66,570,980	68,637,499	2.05 × 10^9^	1.07 × 10^9^	1.29 × 10^8^	1.54 × 10 ^8^
	worst	5,026,634	6.48 × 10^9^	1.03 × 10^8^	1.01 × 10^11^	29,463,946	2.83 × 10^10^	2.57 × 10^9^	1.43 × 10^8^	8.17 × 10^8^	6.51 × 10^9^	2.67 × 10^9^	4.68 × 10^9^	2.25 × 10 ^8^
	std	867,286.6	2.54 × 10^9^	32,204,702	1.87 × 10^10^	11,215,199	7.09 × 10^9^	7.33 × 10^8^	33,904,696	3.49 × 10^8^	1.88 × 10^9^	6.94 × 10^8^	1.97 × 10^9^	35,866,307
	median	3,445,505	2.61 × 10^9^	63,038,260	8.28 × 10^10^	15,501,035	2.14 × 10^10^	1.87 × 10^9^	80,957,677	5.1 × 10^8^	4.24 × 10^9^	2.25 × 10^9^	1.66 × 10^9^	1.98 × 10 ^8^
	rank	1	10	3	13	2	12	7	4	6	11	9	8	5
C17-F13	mean	20,543.43	7.13 × 10^8^	141,800.9	3.67 × 10^10^	21,237.92	7.59 × 10^9^	2.11 × 10^8^	247,156.6	2.47 × 10^8^	5.88 × 10^8^	11,242,163	6.16 × 10^8^	26,005,027
	best	15,376.38	19,099,120	78,023.61	2.48 × 10^10^	16,056.18	5.03 × 10^9^	1.2 × 10^8^	194,166.2	2.17 × 10^8^	3.8 × 10^8^	45,145.04	26,221.25	12,818,531
	worst	27,661.65	1.43 × 10^9^	224,553	5.03 × 10^10^	28,607.16	1.12 × 10^10^	3.77 × 10^8^	308,694.8	3.16 × 10^8^	7.42 × 10^8^	32,680,141	2.46 × 10^9^	54,704,377
	std	5265.513	7.92 × 10^8^	65,454.08	1.08 × 10^10^	5377.922	2.79 × 10^9^	1.18 × 10^8^	52,336.76	46,903,226	1.59 × 10^8^	15,396,915	1.23 × 10^9^	19,804,620
	median	19,567.85	7.03 × 10^8^	132,313.6	3.59 × 10^10^	20,144.16	7.08 × 10^9^	1.75 × 10^8^	242,882.7	2.27 × 10^8^	6.14 × 10^8^	6,121,682	149,734	18,248,601
	rank	1	11	3	13	2	12	7	4	8	9	5	10	6
C17-F14	mean	1597.307	600,173.8	2,218,022	34,471,586	1678.635	5,093,227	4,192,458	279,639.4	1,793,375	1,267,223	4,946,211	240,948.6	11,575,392
	best	1562.632	185,224.1	110,573.9	12,465,227	1639.592	3,093,492	1,735,897	207,111.7	212,073.5	812,745.7	1,782,005	74,420.19	4,799,281
	worst	1617.968	1,430,924	6,732,015	79,830,753	1725.082	9,688,319	7,361,760	412,568.6	5,453,517	2,266,987	8,569,881	688,466.4	14,217,124
	std	24.00143	575,089	3,063,340	31,173,317	35.3101	3,084,397	2,727,676	96,387.31	2,463,724	679,539.8	2,853,620	299,315.4	4,540,074
	median	1604.315	392,273.7	1,014,750	22,795,182	1674.933	3,795,549	3,836,088	249,438.6	753,954.7	994,580.2	4,716,480	100,453.9	13,642,580
	rank	1	5	8	13	2	11	9	4	7	6	10	3	12
C17-F15	mean	2278.122	1,918,426	56,625.99	5.26 × 10^9^	2259.208	1.83 × 10^9^	14,219,521	194,386.3	1.31 × 10^8^	66,651,092	2.11 × 10^8^	17,821,961	12,226,060
	best	2183.166	13,016.53	35,125.41	4.45 × 10^9^	2168.222	1.08 × 10^9^	4,542,309	46,119.64	54,273.51	17,897,687	12,105.45	8042.893	3,671,989
	worst	2463.577	7,144,595	86,519.19	7.14 × 10^9^	2443.118	3.61 × 10^9^	32,284,842	458,767.1	4.32 × 10^8^	1.36 × 10^8^	8.44 × 10^8^	71,236,824	20,439,957
	std	126.4823	3,491,216	21,600.89	1.27 × 10^9^	125.174	1.21 × 10^9^	12,437,330	192,355	2.05 × 10^8^	51,513,207	4.22 × 10^8^	35,609,909	9,283,812
	median	2232.872	258,046.4	52,429.69	4.73 × 10^9^	2212.746	1.31 × 10^9^	10,025,466	136,329.3	45,952,682	56,383,284	21,026.03	21,487.86	12,396,147
	rank	2	5	3	13	1	12	7	4	10	9	11	8	6
C17-F16	mean	2523.35	3142.7	4273.055	7676.857	2524.843	5581.054	7341.921	3652.628	3194.303	5172.976	3615.108	3531.027	3790.935
	best	2248.739	2865.182	3720.895	6111.503	2235.572	4317.16	6419.835	3314.145	2513.073	4937.946	3348.535	3133.521	3640.193
	worst	2849.941	3306.808	4619.853	9059.164	2831.915	6921.292	9089.87	3852.877	3580.118	5485.408	3817.586	4237.175	3978.839
	std	248.4592	191.8193	386.0529	1416.194	249.8163	1340.965	1189.901	237.443	472.1091	246.3174	233.5936	495.8781	140.9372
	median	2497.361	3199.406	4375.736	7768.381	2515.943	5542.882	6928.989	3721.744	3342.01	5134.274	3647.156	3376.706	3772.355
	rank	1	3	9	13	2	11	12	7	4	10	6	5	8
C17-F17	mean	2707.987	2980.2	3569.067	12,417.77	2739.995	8694.875	3849.098	3174.238	2960.83	4242.461	3788.797	3327.726	3554.822
	best	2573.454	2826.593	3286.215	8706.209	2669.604	3961.759	3373.148	2874.678	2707.394	3907.595	3039.955	3129.702	2939.71
	worst	2872.458	3175.288	4177.956	16,710.1	2782.582	20,149.38	4113.558	3356.334	3450.413	4386.915	4362.678	3628.122	3915.836
	std	123.7582	167.3508	419.5302	3897.022	49.62537	7728.123	326.2007	214.7127	335.2494	225.1485	649.6461	218.2146	425.0624
	median	2693.018	2959.46	3406.048	12,127.38	2753.897	5334.178	3954.842	3232.971	2842.756	4337.667	3876.278	3276.54	3681.87
	rank	1	4	8	13	2	12	10	5	3	11	9	6	7
C17-F18	mean	15,216.7	1,365,955	7,779,299	2.14 × 10^8^	15,772.3	25,039,603	63,038,334	2,399,810	4,407,874	6,375,307	6,213,809	688,185.2	9,525,765
	best	6740.69	322,064.1	1,590,180	91,874,841	7191.04	2,203,537	20,713,331	647,337.2	1,422,857	2,612,645	2,233,690	405,300.8	3,454,730
	worst	29,018.95	3,065,480	10,900,590	3.18 × 10^8^	29,848.79	58,520,893	1.17 × 10^8^	4,811,940	7,682,387	10,961,389	13,622,036	1,012,622	22,724,999
	std	9963.231	1,292,998	4,375,760	95,362,782	10,116.69	24,185,228	39,852,952	1,806,889	2,901,609	3,444,641	5,332,511	295,490.3	8,953,276
	median	12,553.59	1,038,138	9,313,213	2.24 × 10^8^	13,024.68	19,716,992	57,291,737	2,069,982	4,263,127	5,963,596	4,499,756	667,408.8	5,961,666
	rank	1	4	9	13	2	11	12	5	6	8	7	3	10
C17-F19	mean	2293.943	5,336,995	217,953.8	5.72 × 10^9^	2277.617	1.71 × 10^9^	6,512,698	3,003,168	615,864.9	52,611,378	339,457.9	14,857.85	1,227,039
	best	2066.933	10,891.04	70,004.28	3.11 × 10^9^	2054.853	7,948,977	775,698.7	387,452.4	129,486.9	28,462,150	167,430.6	9629.5	432,756.2
	worst	2609.612	21,264,994	401,879.8	8.28 × 10^9^	2595.726	4.84 × 10^9^	14,711,626	6,128,420	980,956.3	69,545,870	512,814.9	17,868.24	2,255,991
	std	261.3956	10,618,678	154,738.8	2.17 × 10^9^	261.1937	2.28 × 10^9^	6,365,958	2,718,342	394,103.7	17,554,774	151,196.4	3865.085	866,614.6
	median	2249.614	36,046.9	199,965.6	5.74 × 10^9^	2229.945	9.91 × 10^8^	5,281,734	2,748,399	676,508.2	56,218,747	338,793.1	15,966.83	1,109,704
	rank	2	9	4	13	1	12	10	8	6	11	5	3	7
C17-F20	mean	2513.866	3018.161	3872.479	4208.551	2653.995	3777.941	3867.633	3270.537	3050.774	3581.661	3631.741	3607.874	2793.115
	best	2409.529	2704.176	3404.592	3915.663	2423.39	3344.414	3676.826	3068.736	2867.013	3097.769	3256.531	3122.52	2397.439
	worst	2774.471	3568.838	4297.382	4450.765	2826.888	4059.381	4083.729	3457.862	3422.019	3990.042	4149.514	4033.223	3117.089
	std	174.2807	387.3215	451.866	222.377	182.1288	333.4411	169.5709	187.6622	255.2918	434.451	387.0701	393.726	305.5244
	median	2435.732	2899.815	3893.97	4233.888	2682.851	3853.985	3854.989	3277.775	2957.032	3619.417	3560.461	3637.876	2828.967
	rank	1	4	12	13	2	10	11	6	5	7	9	8	3
C17-F21	mean	2531.346	2696.004	2776.417	3098.527	2534.35	3032.349	3039.543	2554.921	2511.337	2851.206	2879.907	2627.296	2766.011
	best	2463.183	2582.783	2688.564	3029.315	2514.342	2940.893	3024.393	2461.154	2476.733	2807.612	2788.54	2584.675	2734.224
	worst	2606.947	2846.445	2883.738	3183.214	2583.284	3134.125	3059.281	2633.857	2563.453	2927.856	2960.196	2651.486	2811.403
	std	60.93002	117.389	88.55727	64.69718	32.75536	90.8218	14.51323	86.35629	36.99942	54.08873	71.85458	31.25404	32.51022
	median	2527.628	2677.395	2766.683	3090.79	2519.886	3027.189	3037.249	2562.336	2502.581	2834.677	2885.445	2636.512	2759.209
	rank	2	6	8	13	3	11	12	4	1	9	10	5	7
C17-F22	mean	2317.943	10,515.57	9803.788	16,741.71	2454.545	14,725.05	15,009.33	8671.283	10,896.57	16,462.37	11,782.89	10,731.79	8677.909
	best	2313.226	8675.879	8893.579	15,427.18	2415.808	13,406.04	13,752.53	7356.599	8490.107	15,962.22	10,801.92	8673.583	3773.449
	worst	2325.262	12,098.55	10,695.35	17,493.49	2477.608	15,661.92	17,232.92	10,096.67	15,253.11	17,147.82	12,737.27	12,193.1	13,745.36
	std	5.169666	1606.074	736.9605	907.5177	27.54961	992.7556	1638.035	1163.704	2983.966	496.1757	803.0463	1540.999	5393.703
	median	2316.642	10,643.92	9813.11	17,023.08	2462.383	14,916.13	14,525.93	8615.933	9921.535	16,369.72	11,796.17	11,030.25	8596.412
	rank	1	6	5	13	2	10	11	3	8	12	9	7	4
C17-F23	mean	2881.727	3704.484	3392.839	3986.698	3001.709	3953.136	3708.9	3024.442	3027.14	3336.887	4966.53	3421.272	3401.637
	best	2862.544	3619.689	3259.762	3827.63	2935.197	3720.133	3372.6	3000.34	2979.011	3299.768	4857.063	3323.802	3353.64
	worst	2913.196	3884.09	3547.399	4145.13	3064.136	4185.486	4086.312	3040.22	3075.466	3403.308	5023.978	3480.774	3485.028
	std	23.70954	123.6058	119.1142	130.9662	55.49515	198.0927	295.5549	19.24806	39.38033	45.64418	75.0103	67.75003	60.1368
	median	2875.584	3657.078	3382.098	3987.017	3003.752	3953.461	3688.345	3028.604	3027.041	3322.235	4992.539	3440.257	3383.94
	rank	1	9	6	12	2	11	10	3	4	5	13	8	7
C17-F24	mean	3038.787	4028.224	3679.079	4130.017	3146.731	4133.094	3877.877	3172.559	3222.95	3477.013	4262.321	3738.539	3806.884
	best	3027.411	3823.858	3574.61	4017.829	3102.63	3875.483	3746.093	3115.712	3119.397	3454.19	4030.677	3605.374	3716.276
	worst	3060.731	4152.973	3759.596	4253.614	3198.408	4287.089	4065.552	3232.535	3367.039	3517.98	4424.4	3876.766	3921.35
	std	15.66003	149.4732	77.96719	111.4572	41.67069	178.3878	134.3584	54.22258	112.9554	29.21587	172.8838	111.256	85.83412
	median	3033.503	4068.032	3691.055	4124.313	3142.943	4184.901	3849.931	3170.995	3202.681	3467.94	4297.103	3736.008	3794.955
	rank	1	10	6	11	2	12	9	3	4	5	13	7	8
C17-F25	mean	3104.831	4915.11	3209.755	13,225.84	3136.75	6281.772	4139.484	3122.747	3999.108	4985.944	4236.589	3220.005	4438.616
	best	3044.755	3892.863	3148.285	11,160.31	3102.92	5338.942	3508.061	3073.832	3882.131	4335.129	4097.495	3108.568	4286.427
	worst	3234.692	6364.993	3270.78	15,046.82	3206.335	7123.475	4551.233	3182.13	4122.234	5844.257	4494.352	3400.709	4641.84
	std	87.52477	1144.015	52.82601	1623.073	47.17018	867.0227	452.2604	53.32415	134.017	636.2107	183.2711	126.1991	148.101
	median	3069.938	4701.292	3209.978	13,348.12	3118.874	6332.336	4249.321	3117.513	3996.034	4882.195	4177.254	3185.372	4413.098
	rank	1	10	4	13	3	12	7	2	6	11	8	5	9
C17-F26	mean	3777.101	11,784.36	11,856.67	16,041.26	4830.795	14,285.51	15,000.12	5856.907	7468.213	9127.18	13,003.15	7521.003	7269.406
	best	3003.992	10,212.35	11,426.28	14,539.68	3279.002	12,496.3	13,779.67	5489.546	6929.365	7084.218	12,617.15	3631.76	6595.043
	worst	5801.625	13,060.54	12,428.67	17,195.64	6671.97	15,802.77	17,606.04	6441.522	7971.815	10,346.31	14,125.17	11,719.42	8427.224
	std	1351.612	1181.164	483.5606	1116.16	1803.358	1363.859	1798.803	446.8491	447.8152	1472.329	748.1652	3669.866	858.8363
	median	3151.393	11,932.28	11,785.87	16,214.85	4686.104	14,421.48	14,307.38	5748.281	7485.836	9539.094	12,635.14	7366.417	7027.678
	rank	1	8	9	13	2	11	12	3	5	7	10	6	4
C17-F27	mean	3346.281	4472.026	3786.595	5452.493	3327.914	5182.676	4227.945	3352.327	3611.413	3851.884	8321.828	3939.635	4390.701
	best	3271.559	4447.754	3592.211	4995.852	3246.939	4586.378	4036.159	3266.144	3538.938	3636.979	8118.6	3602.202	4114.634
	worst	3437.758	4503.122	3971.15	6241.117	3409.39	6827.707	4419.18	3459.869	3763.451	4242.572	8560.416	4205.319	4525.522
	std	75.91211	27.94272	170.6025	550.8051	68.06794	1097.226	205.0116	84.48407	102.5436	269.3932	215.9268	249.6819	193.2365
	median	3337.904	4468.614	3791.51	5286.501	3327.664	4658.31	4228.22	3341.647	3571.632	3763.992	8304.147	3975.511	4461.325
	rank	2	10	5	12	1	11	8	3	4	6	13	7	9
C17-F28	mean	3306.815	6158.968	3569.591	10,561.72	3346.278	7427.443	4944.849	3307.979	4701.016	5398.659	6125.631	3938.473	5225.484
	best	3283.8	5408.513	3476.534	10,041.12	3295.493	5461.916	4735.216	3275.069	4029.702	4696.886	5596.048	3523.454	4981.759
	worst	3327.69	6870.392	3696.643	11,014.54	3375.563	8220.855	5168.117	3326.009	5536.271	6090.742	6562.538	4583.801	5552.983
	std	19.22675	604.3744	92.23122	410.2028	36.42665	1318.663	177.6043	22.52966	649.6887	667.5129	490.3233	452.8714	282.6891
	median	3307.886	6178.482	3552.594	10,595.6	3357.028	8013.501	4938.032	3315.42	4619.046	5403.504	6171.97	3823.319	5183.596
	rank	1	11	4	13	3	12	7	2	6	9	10	5	8
C17-F29	mean	4205.854	5355.257	5319.407	49,098.17	4174.684	8686.831	7487.548	4850.099	4854.946	6606.589	7835.099	5294.549	6323.442
	best	3962.386	5001.086	4933.961	15,947.98	3927.712	7669.135	6357.075	4589.776	4704.047	6391.657	7520.297	4554.894	5749.058
	worst	4527.838	5546.877	6107.551	118,514.9	4502.275	10,907.45	9208.731	5255.592	5139.24	6864.138	8483.466	5688.888	6721.22
	std	255.5898	243.3059	534.441	47,222.41	261.5826	1524.258	1215.726	285.2666	201.9437	211.9127	438.8483	515.1086	438.2013
	median	4166.596	5436.532	5118.058	30,964.87	4134.375	8085.372	7192.193	4777.515	4788.248	6585.281	7668.318	5467.208	6411.745
	rank	2	7	6	13	1	12	10	3	4	9	11	5	8
C17-F30	mean	1,618,080	48,059,983	21,703,729	5.82 × 10^9^	1,780,483	1.03 × 10^9^	3.21 × 10^8^	71,527,639	1.65 × 10^8^	3.21 × 10^8^	2.11 × 10^8^	5,128,712	68,405,882
	best	1,424,086	17,155,339	15,119,492	4.73 × 10^9^	1,414,569	1.63 × 10^8^	1.89 × 10^8^	35,867,005	1.3 × 10^8^	2.34 × 10^8^	1.61 × 10^8^	1,827,095	51,385,322
	worst	1,836,627	1.01 × 10^8^	25,861,408	6.83 × 10^9^	2,524,633	2.59 × 10^9^	6.72 × 10^8^	1.02 × 10^8^	1.91 × 10^8^	4.15 × 10^8^	3.12 × 10^8^	9,012,936	1.08 × 10 ^8^
	std	218,934.4	36,779,462	5,053,503	8.63 × 10^8^	521,656.5	1.07 × 10^9^	2.34 × 10^8^	29,861,371	26,124,552	79,867,440	69,575,182	3,196,822	26,878,690
	median	1,605,803	36,826,199	22,917,007	5.86 × 10^9^	1,591,365	6.78 × 10^8^	2.12 × 10^8^	74,309,997	1.69 × 10^8^	3.18 × 10^8^	1.86 × 10^8^	4,837,409	56,976,059
	rank	1	5	4	13	2	12	10	7	8	11	9	3	6
Sum rank		34	220	183	363	60	326	285	119	151	255	258	167	218
Mean rank		1.172414	7.586207	6.310345	12.51724	2.068966	11.24138	9.827586	4.103448	5.206897	8.793103	8.896552	5.758621	7.517241
Total rank		1	8	6	13	2	12	11	3	4	9	10	5	7

**Table 5:** Optimization results of the CEC 2017 test suite (dimension m=100).

		GAO	WSO	AVOA	RSA	MPA	TSA	WOA	MVO	GWO	TLBO	GSA	PSO	GA
C17-F1	mean	20,912.44	1.22 × 10^11^	7.46 × 10^9^	2.51 × 10^11^	3.74 × 10^8^	1.34 × 10^11^	6.7 × 10^10^	73,581,256	5.68 × 10^10^	9.84 × 10^10^	1.4 × 10^11^	2.75 × 10^10^	6.05 × 10 ^10^
	best	16,541.69	1.13 × 10^11^	2.3 × 10^9^	2.35 × 10^11^	2.41 × 10^8^	1.17 × 10^11^	6.23 × 10^10^	64,783,107	3.7 × 10^10^	8.32 × 10^10^	1.32 × 10^11^	1.52 × 10^10^	5.23 × 10 ^10^
	worst	27,717.39	1.4 × 10^11^	1.49 × 10^10^	2.57 × 10^11^	5.14 × 10^8^	1.51 × 10^11^	7.38 × 10^10^	92,516,372	7.17 × 10^10^	1.15 × 10^11^	1.59 × 10^11^	4.11 × 10^10^	6.66 × 10 ^10^
	std	4828.014	1.26 × 10^10^	5.76 × 10^9^	1.06 × 10^10^	1.19 × 10^8^	1.37 × 10^10^	4.92 × 10^9^	12,778,487	1.48 × 10^10^	1.31 × 10^10^	1.3 × 10^10^	1.1 × 10^10^	6.37 × 10 ^9^
	median	19,695.34	1.19 × 10^11^	6.33 × 10^9^	2.56 × 10^11^	3.71 × 10^8^	1.34 × 10^11^	6.59 × 10^10^	68,512,773	5.92 × 10^10^	9.77 × 10^10^	1.34 × 10^11^	2.68 × 10^10^	6.15 × 10 ^10^
	rank	1	10	4	13	3	11	8	2	6	9	12	5	7
C17-F3	mean	167,218.3	311,047.9	458,990.6	337,914.4	171,436.5	374,031.9	967,631.7	518,487.4	420,776.6	335,622.8	364,793.5	552,885.3	685,800.1
	best	148,279.5	293,703.8	336,492.7	328,256.1	154,520.4	301,173.3	885,141.3	412,763.8	351,353.7	306,622.4	351,921.6	434,606.2	550,559.2
	worst	180,601	322,076.4	771,655.5	357,719.6	201,283.7	505,054.8	1,131,352	670,584.5	474,359.5	367,271.7	373,433.4	627,862.4	796,742.2
	std	14,839.17	12,529.05	209,003.2	13,446.05	21,330.5	90,345.93	111,751.1	108,653.9	51,046.89	26,918.59	9128.358	86,478.11	101,484.8
	median	169,996.3	314,205.6	363,907	332,840.9	164,970.9	344,949.8	927,016.5	495,300.6	428,696.6	334,298.5	366,909.6	574,536.2	697,949.5
	rank	1	3	9	5	2	7	13	10	8	4	6	11	12
C17-F4	mean	655.7039	21,774.05	1662.224	73,321.34	1186.412	17,907.78	11,191.2	739.5621	5093.534	12,385.77	31,842.3	4276.123	8698.021
	best	595.8195	17,433.85	1398.495	43,521.41	1108.667	13,665.86	9760.209	694.4664	3321.202	9715.491	25,588.62	1435.661	6860.208
	worst	696.4627	29,917.72	1851.454	94,391.08	1306.893	22,129.5	12,369.16	790.3274	6945.488	15,480.88	39,465.68	6678.925	9846.347
	std	44.90914	5564.829	192.679	22,193.54	94.64766	3742.318	1075.33	47.49333	1541.883	2413.397	5724.487	2370.731	1284.409
	median	665.2666	19,872.32	1699.474	77,686.43	1165.044	17,917.87	11,317.72	736.7272	5053.723	12,173.36	31,157.44	4494.953	9042.765
	rank	1	11	4	13	3	10	8	2	6	9	12	5	7
C17-F5	mean	1166.05	1504.914	1350.044	2009.134	1210.08	2172.868	1906.086	1221.411	1205.445	1820.496	1355.463	1507.352	1723.655
	best	1139.731	1380.87	1304.366	1986.939	1179.843	2032.825	1870.109	1129.691	1156.931	1742.453	1278.82	1430.764	1639.415
	worst	1218.839	1589.623	1380.923	2020.928	1252.769	2302.787	1925.408	1314.041	1262.361	1892.273	1402.39	1585.082	1847.619
	std	36.21496	88.21671	34.10216	15.29979	32.3499	110.4274	25.54385	87.66381	43.43123	62.82961	57.11354	78.18066	88.17999
	median	1152.816	1524.582	1357.444	2014.335	1203.855	2177.931	1914.413	1220.955	1201.244	1823.63	1370.32	1506.78	1703.792
	rank	1	7	5	12	3	13	11	4	2	10	6	8	9
C17-F6	mean	633.3793	669.854	666.8515	714.5884	662.3248	728.0069	694.2372	669.198	643.6109	689.9683	667.5834	666.0791	665.0116
	best	628.2662	661.8741	663.6147	710.3046	653.4113	712.535	688.9922	662.0385	636.1507	680.4107	666.9654	660.5087	657.8043
	worst	639.7835	676.3993	672.4559	718.0482	666.7799	762.8446	700.7457	676.3073	652.1949	697.2029	668.868	669.1201	672.3328
	std	4.822856	6.562621	3.937282	3.511939	6.083093	23.39071	4.905194	7.80358	7.689992	7.061908	0.87649	3.82012	6.884498
	median	632.7338	670.5712	665.6676	715.0005	664.554	718.324	693.6055	669.223	643.049	691.1298	667.2501	667.3438	664.9546
	rank	1	9	6	12	3	13	11	8	2	10	7	5	4
C17-F7	mean	1911.916	3541.744	3243.381	3801.276	2020.946	3797.222	3490.289	2081.918	2095.396	3191.277	2858.94	2791.773	2563.793
	best	1816.72	3175.644	3123.082	3776.709	1843.88	3595.851	3387.53	1987.152	1985.72	3087.528	2696.081	2652.524	2490.104
	worst	1996.955	3681.703	3366.049	3834.327	2186.48	4075.989	3651.572	2150.043	2257.854	3285.241	3105.104	2969.625	2644.502
	std	73.84701	245.1298	120.3608	24.22757	156.5285	202.1429	117.843	76.32504	121.5581	87.36305	177.2088	131.4316	65.7947
	median	1916.994	3654.815	3242.197	3797.034	2026.713	3758.525	3461.026	2095.239	2069.004	3196.17	2817.288	2772.471	2560.284
	rank	1	11	9	13	2	12	10	3	4	8	7	6	5
C17-F8	mean	1479.939	1799.874	1773.64	2533.999	1539.319	2622.936	2272.001	1532.856	1557.506	2176.459	1849.688	1766.43	2092.383
	best	1390.582	1630.939	1719.477	2493.042	1470.086	2469.493	2151.262	1404.611	1497.673	2098.286	1770.871	1656.793	2059.065
	worst	1576.255	1873.937	1874.707	2623.134	1600.872	2768.885	2436.535	1648.465	1614.262	2245.317	1887.851	1900.201	2137.38
	std	81.26873	114.9677	72.14627	61.50208	67.5484	155.8935	120.0276	99.83087	52.78256	71.14839	53.28624	101.1641	32.69375
	median	1476.459	1847.311	1750.188	2509.911	1543.158	2626.682	2250.103	1539.175	1559.044	2181.117	1870.014	1754.364	2086.543
	rank	1	7	6	12	3	13	11	2	4	10	8	5	9
C17-F9	mean	18,988.95	75,301.69	27,474.52	78,515.61	20,581.98	119,729.8	76,615.32	69,359.17	51,589.73	73,206.16	25,144.88	31,675.48	49,133.87
	best	17,534.05	70,403.42	24,021.24	75,751.77	17,157.09	78,515.21	44,853.75	52,525.18	37,346.56	69,094.37	24,224.53	28,350.56	47,316.16
	worst	19,864.72	82,522.17	30,100.94	80,276.88	22,978.62	170,562.3	120,610.8	82,118.82	62,334.65	81,821.5	26,765.88	34,019.72	50,386.03
	std	1014.979	5698.842	2533.883	2044.079	2478.55	38,430.28	31,945.82	15,039.91	10,826.22	5903.298	1129.28	2717.236	1313.108
	median	19,278.51	74,140.59	27,887.94	79,016.89	21,096.12	114,920.9	70,498.37	71,396.33	53,338.85	70,954.38	24,794.55	32,165.82	49,416.65
	rank	1	10	4	12	2	13	11	8	7	9	3	5	6
C17-F10	mean	14,359.39	19,399.22	17,935.55	30,430.88	15,397.45	29,410.51	27,898.61	15,980.88	15,828.8	31,963.1	17,938.09	18,456.5	27,359.06
	best	13,588.52	17,136.26	16,133.09	29,343.9	13,863.46	27,642.05	25,792.07	15,470.12	13,970.59	30,839.26	17,158.98	17,362.09	26,489.39
	worst	15,288.83	20,569.25	20,986.91	30,959.34	17,011.82	31,013.61	29,273.82	17,003.14	17,570.76	32,443.01	19,079.19	19,693.2	28,130.44
	std	700.8977	1553.247	2192.594	750.2732	1304.291	1380.703	1485.612	698.9395	1896.195	753.3583	914.5259	1156.665	729.5295
	median	14,280.1	19,945.69	17,311.1	30,710.13	15,357.26	29,493.18	28,264.27	15,725.13	15,886.93	32,285.06	17,757.09	18,385.36	27,408.21
	rank	1	8	5	12	2	11	10	4	3	13	6	7	9
C17-F11	mean	2283.281	82,550.02	70,967.33	208,606.7	5155.297	79,645.63	196,177.6	4901.862	64,611.18	53,294.21	167,260.4	56,864.81	173,971.6
	best	2174.672	64,588.68	57,187.83	155,577.6	3452.397	53,901.98	138,002.8	4238.748	45,058.24	35,164.28	151,438	41,683.31	118,220.4
	worst	2372.435	109,454.1	86,372.24	274,283.1	9341.941	107,434.5	324,831.2	5676.522	89,892.73	70,199.98	178,425.9	81,149.72	229,857
	std	85.50501	19,373.98	12,757.85	50,594.08	2813.694	28,079.87	87,120.08	627.6838	19,176.61	14,322.73	12,269.74	17,149.44	55,041.7
	median	2293.009	78,078.65	70,154.63	202,283	3913.425	78,623	160,938.1	4846.089	61,746.87	53,906.3	169,588.8	52,313.1	173,904.5
	rank	1	9	7	13	3	8	12	2	6	4	10	5	11
C17-F12	mean	13,549,934	4.59 × 10^10^	6.22 × 10^8^	1.7 × 10^11^	2.54 × 10^8^	5.69 × 10^10^	1.14 × 10^10^	5.16 × 10^8^	1.23 × 10^10^	3.82 × 10^10^	6.69 × 10^10^	1.9 × 10^10^	1.1 × 10 ^10^
	best	11,051,434	1.56 × 10^10^	4.81 × 10^8^	1.48 × 10^11^	1.19 × 10^8^	5.06 × 10^10^	7.91 × 10^9^	3.3 × 10^8^	3.83 × 10^9^	2.95 × 10^10^	5.8 × 10^10^	3.22 × 10^9^	7.79 × 10 ^9^
	worst	17,094,242	5.88 × 10^10^	8.18 × 10^8^	1.91 × 10^11^	3.27 × 10^8^	6.74 × 10^10^	1.74 × 10^10^	6.22 × 10^8^	1.97 × 10^10^	4.86 × 10^10^	7.9 × 10^10^	4.81 × 10^10^	1.58 × 10 ^10^
	std	2,575,896	2.03 × 10^10^	1.43 × 10^8^	1.9 × 10^10^	92,551,357	7.83 × 10^9^	4.29 × 10^9^	1.3 × 10^8^	6.92 × 10^9^	7.93 × 10^9^	9.52 × 10^9^	2 × 10^10^	3.38 × 10 ^9^
	median	13,027,031	5.45 × 10^10^	5.94 × 10^8^	1.71 × 10^11^	2.85 × 10^8^	5.48 × 10^10^	1.02 × 10^10^	5.56 × 10^8^	1.27 × 10^10^	3.74 × 10^10^	6.54 × 10^10^	1.24 × 10^10^	1.02 × 10 ^10^
	rank	1	10	4	13	2	11	6	3	7	9	12	8	5
C17-F13	mean	36,350.96	9.2 × 10^9^	61,970.35	4.42 × 10^10^	89,467.32	1.92 × 10^10^	5.41 × 10^8^	442,954.8	8.32 × 10^8^	3.03 × 10^9^	7.97 × 10^9^	2.78 × 10^9^	2.57 × 10 ^8^
	best	32,150.66	4.58 × 10^9^	33,152.4	3.64 × 10^10^	65,042.21	1.75 × 10^10^	5.04 × 10^8^	308,678.7	5.15 × 10^8^	2.32 × 10^9^	4.54 × 10^9^	1.19 × 10^8^	70,258,995
	worst	43,505.81	1.16 × 10^10^	86,223.3	5.01 × 10^10^	146,358.2	2.2 × 10^10^	6.07 × 10^8^	718,468.5	1.05 × 10^9^	4.29 × 10^9^	1.21 × 10^10^	6.47 × 10^9^	5.14 × 10 ^8^
	std	5095.066	3.22 × 10^9^	22,345.65	5.9 × 10^9^	38,124.21	2.16 × 10^9^	46,063,980	192,117.4	2.25 × 10^8^	8.9 × 10^8^	3.36 × 10^9^	2.68 × 10^9^	1.89 × 10 ^8^
	median	34,873.67	1.03 × 10^10^	64,252.85	4.51 × 10^10^	73,234.46	1.86 × 10^10^	5.27 × 10^8^	372,336	8.84 × 10^8^	2.74 × 10^9^	7.64 × 10^9^	2.28 × 10^9^	2.21 × 10 ^8^
	rank	1	11	2	13	3	12	6	4	7	9	10	8	5
C17-F14	mean	37,460.72	4,489,152	4,456,875	1.33 × 10^8^	37,144.92	12,044,625	12,300,656	1,746,857	8,738,866	9,298,250	11,237,202	2,132,081	14,691,061
	best	16,361.15	1,757,714	1,824,839	85,227,980	16,203.21	2,383,941	4,250,213	436,362.4	4,548,999	4,212,378	7,377,841	774,496.8	13,230,443
	worst	75,561.08	6,961,340	6,473,059	1.59 × 10^8^	74,945.98	23,254,221	21,219,928	3,559,704	17,471,114	13,688,403	14,581,126	4,060,274	16,041,918
	std	26,110.58	2,232,142	2,151,003	33,511,442	25,905.58	8,976,986	7,301,106	1,485,338	6,023,595	4,478,970	3,814,456	1,471,762	1,151,485
	median	28,960.31	4,618,777	4,764,802	1.44 × 10^8^	28,715.24	11,270,170	11,866,241	1,495,681	6,467,675	9,646,109	11,494,920	1,846,777	14,745,941
	rank	2	6	5	13	1	10	11	3	7	8	9	4	12
C17-F15	mean	18,882.03	98,686,181	48,987.56	2.29 × 10^10^	57,030.81	6.86 × 10^9^	1.36 × 10^8^	164,097.1	4.37 × 10^8^	9.37 × 10^8^	1.5 × 10^9^	2.37 × 10^8^	11,419,474
	best	13,638.59	3,225,436	33,301.29	1.34 × 10^10^	31,982.54	2.06 × 10^9^	87,407,866	138,892.2	1.87 × 10^8^	5.5 × 10^8^	9.05 × 10^8^	15,041.64	9,550,801
	worst	26,884.85	3.12 × 10^8^	63,801.66	2.79 × 10^10^	83,136.76	1.02 × 10^10^	2.33 × 10^8^	196,300.4	8.23 × 10^8^	1.93 × 10^9^	1.96 × 10^9^	9.46 × 10^8^	14,186,872
	std	5961.071	1.46 × 10^8^	12,585.04	6.49 × 10^9^	21,039.18	3.58 × 10^9^	67,067,445	27,529.44	2.72 × 10^8^	6.66 × 10^8^	4.45 × 10^8^	4.73 × 10^8^	2,084,089
	median	17,502.33	39,800,118	49,423.64	2.51 × 10^10^	56,501.97	7.6 × 10^9^	1.13 × 10^8^	160,597.8	3.68 × 10^8^	6.31 × 10^8^	1.56 × 10^9^	91,930.12	10,970,111
	rank	1	6	2	13	3	12	7	4	9	10	11	8	5
C17-F16	mean	5159.521	7911.144	7237.618	20,529.48	5775.412	13,231.82	17,536.37	6208.623	6155.142	11,501.78	11,288.39	6462.738	10,775.66
	best	4482.443	7594.345	6297.251	18,189.53	4964.557	11,674.5	13,541.34	6171.325	5459.267	10,272.69	10,267.51	6118.349	9633.615
	worst	5632.458	8242.705	8100.535	25,016.12	6480.428	15,062.96	23,606.65	6271.911	6685.123	13,117.57	13,583.71	6976.423	12,105.7
	std	548.9745	326.0461	743.9669	3052.189	627.8321	1402.246	4344.409	43.90544	606.2982	1190.737	1543.044	368.3862	1022.479
	median	5261.591	7903.763	7276.342	19,456.13	5828.332	13,094.92	16,498.74	6195.627	6238.088	11,308.44	10,651.17	6378.091	10,681.67
	rank	1	7	6	13	2	11	12	4	3	10	9	5	8
C17-F17	mean	4143.881	17,256.29	6278.188	7,660,577	4214.135	244,517.8	11,453.29	5751.307	4819.8	9430.942	33,565.25	9477.168	7289.964
	best	3746.746	6565.42	5371.806	814,252.4	3859.68	9857.97	7790.231	5185.425	4232.158	8686.086	15,047.03	5453.346	7193.519
	worst	4504.818	34,680.9	7197.238	14,282,162	4387.087	380,666	15,834.67	6161.67	5523.21	10,606.71	50,021.65	19,192.23	7395.844
	std	313.4527	12,940.4	753.5212	7,595,286	239.6748	174,764	3629.996	477.3327	646.7451	822.6179	14,668.01	6500.52	105.6253
	median	4161.981	13,889.42	6271.853	7,772,947	4304.886	293,773.5	11,094.13	5829.067	4761.917	9215.487	34,596.15	6631.55	7285.246
	rank	1	10	5	13	2	12	9	4	3	7	11	8	6
C17-F18	mean	140,921.8	3,264,581	2,476,386	1.01 × 10^8^	334,853.4	17,184,831	12,263,458	5,264,912	10,835,731	18,034,198	8,025,842	13,669,910	10,129,900
	best	137,676.2	1,736,452	2,182,566	68,971,163	178,243.2	5,271,540	12,006,141	2,407,412	3,654,670	13,158,106	5,426,248	952,614.7	7,733,772
	worst	142,889	5,059,186	2,906,906	1.51 × 10^8^	527,595.6	42,365,816	12,508,264	8,765,635	17,524,717	22,851,226	13,174,384	36,223,091	12,868,662
	std	2286.571	1,383,907	339,182.7	38,420,398	167,720.4	17,004,610	246,545.2	2,622,353	7,333,488	4,899,214	3,491,428	16,329,859	2,130,627
	median	141,561	3,131,343	2,408,037	92,865,432	316,787.5	10,550,984	12,269,714	4,943,301	11,081,769	18,063,731	6,751,367	8,751,967	9,958,583
	rank	1	4	3	13	2	11	9	5	8	12	6	10	7
C17-F19	mean	161,069.6	8.42 × 10^8^	1,205,021	2.31 × 10^10^	169,321.7	8.41 × 10^9^	1.11 × 10^8^	20,443,070	66,702,321	4.82 × 10^8^	4.09 × 10^8^	4,904,826	17,196,651
	best	38,442.77	97,622,306	718,063.4	2.01 × 10^10^	40,312.61	1.21 × 10^9^	56,321,277	11,725,752	13,898,824	4.36 × 10^8^	1.57 × 10^8^	142,122.3	13,449,014
	worst	402,093.3	1.63 × 10^9^	1,913,021	2.63 × 10^10^	411,397	1.62 × 10^10^	1.63 × 10^8^	26,686,851	1.47 × 10^8^	5.08 × 10^8^	6.21 × 10^8^	10,867,115	23,529,997
	std	165,447.5	6.67 × 10^8^	507,442.8	2.86 × 10^9^	167,915.7	6.13 × 10^9^	57,996,119	6,580,111	57,324,646	31,934,561	2.08 × 10^8^	5,311,723	4,425,910
	median	101,871.1	8.2 × 10^8^	1,094,500	2.29 × 10^10^	112,788.6	8.13 × 10^9^	1.13 × 10^8^	21,679,838	52,857,305	4.93 × 10^8^	4.3 × 10^8^	4,305,033	15,903,796
	rank	1	11	3	13	2	12	8	6	7	10	9	4	5
C17-F20	mean	4329.772	5183.389	6066.485	7643.89	4432.637	7040.717	6551.851	5158.041	7077.601	7324.316	6241.91	5196.925	6328.018
	best	3875.227	3852.122	5777.4	7182.534	4170.89	6256.34	6048.655	4700.095	4372.886	6860.291	5444.499	4783.879	5957.546
	worst	4639.907	7866.15	6394.717	7875.16	4595.62	7699.216	6923.305	5389.048	8042.899	7811.819	6908.555	6022.863	7197.281
	std	323.251	1819.666	277.5492	321.5951	185.1203	658.1345	389.4126	319.7632	1804.006	486.1558	772.4079	560.5865	583.2411
	median	4401.977	4507.641	6046.912	7758.933	4482.019	7103.656	6617.722	5271.51	7947.309	7312.577	6307.294	4990.479	6078.622
	rank	1	4	6	13	2	10	9	3	11	12	7	5	8
C17-F21	mean	2764.742	4027.213	3664.091	4458.728	2881.877	4335.331	4326.582	3036.657	2992.63	3790.589	4708.698	3666.636	3675.619
	best	2699.974	3704.178	3568.045	4347.035	2766.476	4068.898	4124.048	2916.202	2896.614	3678.986	4581.198	3504.164	3582.929
	worst	2816.339	4369.58	3764.376	4614.097	3011.426	4510.539	4598.794	3226.835	3070.04	3883.529	4980.425	3849.175	3751.677
	std	48.11086	278.4377	108.195	113.2218	101.1698	209.0187	200.4712	133.2488	85.81598	85.11658	185.487	143.4907	73.69636
	median	2771.327	4017.547	3661.971	4436.889	2874.802	4380.944	4291.742	3001.796	3001.933	3799.921	4636.585	3656.602	3683.934
	rank	1	9	5	12	2	11	10	4	3	8	13	6	7
C17-F22	mean	13,298.62	21,305.75	19,844.61	34,680.71	15,016.95	31,613.32	30,881.75	18,865.96	19,485.72	34,416.29	21,926.57	22,621.27	27,306.94
	best	2440.431	20,323.85	19,177.65	34,605.55	2499.552	31,067.87	29,276.12	18,173.68	18,244.98	34,250.53	19,895.16	20,370.87	16,116.15
	worst	18,623.41	22,434.61	21,090.76	34,718.52	20,013.91	32,297.65	33,319.26	19,981.28	21,249.71	34,868.52	23,369.69	25,034.51	32,184.45
	std	7377.823	876.5613	869.2077	51.0533	8365.608	508.4395	1796.155	858.4155	1379.083	301.7408	1591.5	1915.238	7562.745
	median	16,065.31	21,232.28	19,555.01	34,699.38	18,777.17	31,543.87	30,465.81	18,654.43	19,224.09	34,273.06	22,220.71	22,539.84	30,463.59
	rank	1	6	5	13	2	11	10	3	4	12	7	8	9
C17-F23	mean	3259.665	5159.671	4303.583	5617.314	3459.061	5770.667	5175.464	3603.464	3674.039	4347.467	7897.46	4725.29	4346.781
	best	3226.557	4861.07	4085.871	5421.661	3407.754	5295.597	4895.709	3346.847	3584.29	4256.869	7254.263	4629.852	4136.554
	worst	3296.342	5699.833	4502.895	5715.713	3515.118	6042.383	5395.633	3720.109	3785.023	4413.904	8483.524	4814.118	4536.905
	std	32.90116	386.3156	175.7608	135.368	51.20413	343.2099	210.6716	176.5002	101.0517	65.61731	525.3169	81.32892	167.7969
	median	3257.88	5038.891	4312.782	5665.941	3456.686	5872.344	5205.258	3673.45	3663.42	4359.547	7926.026	4728.594	4356.833
	rank	1	9	5	11	2	12	10	3	4	7	13	8	6
C17-F24	mean	3846.516	7096.235	5033.276	9584.647	3813.223	6948.276	6335.232	4062.592	4365.648	4951.757	10,763.26	5983.513	5331.613
	best	3726.817	6637.348	4771.243	7955.331	3700.374	6119.945	5877.806	3977.801	4253.26	4854.884	10,430.78	5485.013	4888.407
	worst	3971.617	7846.225	5494.416	13,585.21	3934.351	7597.833	6803.973	4155.626	4547.109	5135.507	11,247.97	6457.987	5566.537
	std	124.4288	523.1172	316.9777	2688.925	120.7238	648.2421	447.6772	84.17781	128.9378	128.2864	373.6677	414.727	304.9964
	median	3843.816	6950.683	4933.723	8399.024	3809.084	7037.664	6329.574	4058.471	4331.112	4908.319	10,687.15	5995.526	5435.753
	rank	2	11	6	12	1	10	9	3	4	5	13	8	7
C17-F25	mean	3469.147	11,520.64	4010.435	24,401.33	3902.052	11,610.18	8130.608	3434.111	6741.259	10,248.33	10,694.92	4519.774	8918.261
	best	3336.562	9524.987	3859.072	21,905.88	3795.043	10,384.3	6094.109	3337.136	5476.952	9143.045	9641.655	4026.406	8522.142
	worst	3674.345	13,862.05	4141.137	27,073.78	4001.032	13,671.79	9181.291	3482.374	8276.049	12,322.1	11,956.99	4767.238	9227.662
	std	151.6605	1810.362	145.9816	2285.452	87.68001	1426.465	1425.184	68.04092	1159.786	1421.174	1081.266	340.7136	293.0999
	median	3432.84	11,347.75	4020.765	24,312.84	3906.067	11,192.31	8623.515	3458.468	6606.018	9764.085	10,590.51	4642.725	8961.621
	rank	2	11	4	13	3	12	7	1	6	9	10	5	8
C17-F26	mean	11,776.87	32,319.52	25,892.01	50,965.07	12,256.12	35,938.6	37,175.46	13,381.42	16,826.64	23,075.68	35,000.86	19,788.26	25,645.44
	best	11,355.12	29,278.54	21,112	45,933.69	11,587.21	33,402.26	31,343.9	12,486.47	14,857.57	21,221.76	33,204.49	18,887.49	22,138.29
	worst	12,047.28	35,287.08	28,834.86	53,243.92	12,666.16	37,240.48	42,580.56	13,918.71	18,847.41	25,247.56	36,279.13	20,917.45	29,733.71
	std	298.4586	2627.895	3410.424	3447.125	509.1323	1732.521	4886.416	654.5382	1660.057	1677.974	1380.682	881.7495	3999.153
	median	11,852.55	32,356.22	26,810.59	52,341.34	12,385.56	36,555.83	37,388.69	13,560.26	16,800.79	22,916.7	35,259.91	19,674.06	25,354.89
	rank	1	9	8	13	2	11	12	3	4	6	10	5	7
C17-F27	mean	3575.611	6742.95	4375.433	11,679.1	3675.833	6411.141	5820.97	3703.331	4205.741	4165.307	14,697.54	4056.367	5266.088
	best	3545.734	6081.846	4098.366	8782.023	3592.46	4900.306	4987.661	3563.203	4069.706	4008.832	13,470.22	3649.808	4973.669
	worst	3648.183	7055.009	4747.855	15,162.9	3770.239	7570.592	6846.795	3795.476	4329.609	4332.615	15,679.32	4475.324	5397.056
	std	48.70852	449.6358	313.9328	3294.659	72.86347	1111.333	817.6242	111.1698	109.3868	175.8536	998.4654	370.4368	198.3472
	median	3554.263	6917.473	4327.755	11,385.73	3670.317	6586.834	5724.713	3727.322	4211.825	4159.891	14,820.31	4050.168	5346.814
	rank	1	11	7	12	2	10	9	3	6	5	13	4	8
C17-F28	mean	3602.453	14,553.69	5002.45	29,423.48	3822.111	16,592.32	13,193.5	3503.023	8836.034	12,235.73	20,761.73	7955.561	12,025.11
	best	3543.474	12,197.62	4606.601	25,865.71	3780.886	15,763.65	11,910.22	3439.876	8379.33	10,859.02	18,468.59	5177.514	11,155.88
	worst	3703.404	17,079.89	5431.228	31,600.92	3884.097	17,453.01	14,204.6	3559.923	9513.755	13,945.58	23,291.08	14,077.41	12,859.14
	std	70.29083	2305.749	348.1724	2544.344	49.01329	689.9281	1102.437	49.75649	505.5543	1309.35	2054.307	4125.024	773.8027
	median	3581.466	14,468.62	4985.986	30,113.64	3811.731	16,576.32	13,329.6	3506.147	8725.526	12,069.16	20,643.62	6283.658	12,042.7
	rank	2	10	4	13	3	11	9	1	6	8	12	5	7
C17-F29	mean	7186.205	12,015.6	9814.17	748,982.1	7395.086	66,078.18	18,573.42	7827.296	8639.945	13,867.9	24,083.25	8587.316	12,369.37
	best	6818.963	11,515.44	9111.304	624,851.1	7048.406	28,968.98	17,146.3	7510.98	8235.103	12,396	18,510.53	7668.281	12,005.44
	worst	7577.929	12,378.38	10,408.74	914,832.3	7728.099	123,745.8	20,612.32	8300.293	9067.168	16,357.95	31,591.21	9711.056	12,680.52
	std	325.5348	384.1453	544.8654	120,932	292.5936	42,192.39	1496.769	380.1452	341.9508	1721.132	6312.584	882.8139	286.5164
	median	7173.964	12,084.29	9868.321	728,122.6	7401.919	55,798.97	18,267.52	7748.955	8628.755	13,358.83	23,115.64	8484.963	12,395.76
	rank	1	7	6	13	2	12	10	3	5	9	11	4	8
C17-F30	mean	3,304,314	7.07 × 10^9^	47,858,131	4.4 × 10^10^	6,031,607	1.4 × 10^10^	1.16 × 10^9^	1.25 × 10^8^	2.67 × 10^9^	3.03 × 10^9^	1.04 × 10^10^	6.76 × 10^8^	5.53 × 10 ^8^
	best	1,736,868	4.42 × 10^9^	22,468,989	3.76 × 10^10^	4,802,998	9.63 × 10^9^	8.74 × 10^8^	84,489,086	78,558,419	1.3 × 10^9^	7.8 × 10^9^	10,552,576	3.46 × 10 ^8^
	worst	4,405,575	9.95 × 10^9^	74,195,320	4.81 × 10^10^	6,809,589	1.85 × 10^10^	1.82 × 10^9^	2.01 × 10^8^	7.59 × 10^9^	5.85 × 10^9^	1.31 × 10^10^	1.75 × 10^9^	7.86 × 10 ^8^
	std	1,122,253	2.38 × 10^9^	21,902,086	4.55 × 10^9^	864,695.5	3.76 × 10^9^	4.52 × 10^8^	52,735,484	3.38 × 10^9^	1.99 × 10^9^	2.32 × 10^9^	8.38 × 10^8^	1.89 × 10 ^8^
	median	3,537,407	6.96 × 10^9^	47,384,107	4.51 × 10^10^	6,256,920	1.39 × 10^10^	9.62 × 10^8^	1.06 × 10^8^	1.51 × 10^9^	2.5 × 10^9^	1.03 × 10^10^	4.72 × 10^8^	5.41 × 10 ^8^
	rank	1	10	3	13	2	12	7	4	8	9	11	6	5
Sum rank		33	247	148	359	66	324	275	109	160	251	274	181	212
Mean rank		1.137931	8.517241	5.103448	12.37931	2.275862	11.17241	9.482759	3.758621	5.517241	8.655172	9.448276	6.241379	7.310345
Total rank		1	8	4	13	2	12	11	3	5	9	10	6	7

**Table 6:** Optimization results of the CEC 2019 test suite.

		GAO	WSO	AVOA	RSA	MPA	TSA	WOA	MVO	GWO	TLBO	GSA	PSO	GA
C19-F1	mean	1	71,810.88	1	1	1	20,217.93	11,711,987	1,133,810	27,216.59	68.42838	6.21 × 10^8^	149,696.8	7,924,820
	best	1	1162.052	1	1	1	2.756454	2,455,600	376,289.6	1.004815	1.001003	1.49 × 10^8^	14,365.34	2,790,162
	worst	1	205,671.3	1	1	1	80,699.62	16,695,679	2,287,378	94,807.29	270.6854	1.07 × 10^9^	476,077	16,698,761
	std	0	92,810.22	0	0	0	40,361.5	6,381,017	842,939.7	45,586.06	134.9729	4.66 × 10^8^	218,680.9	6,548,692
	median	1	40,205.1	1	1	1	84.67389	13,848,335	935,786	7029.039	1.013541	6.33 × 10^8^	54,172.42	6,105,179
	rank	1	5	1	1	1	3	9	7	4	2	10	6	8
C19-F2	mean	1	173.085	4.697685	5.00375	3.016487	801.2333	6143.321	564.3356	454.8589	667.6561	27,762.24	364.2709	858.2781
	best	1	98.73351	4.308172	5	2.553251	629.09	2910.908	286.6219	210.5741	5.569583	9861.117	227.0451	578.5317
	worst	1	267.0959	5.005	5.005	3.5079	938.0872	9275.1	838.9716	658.7508	1191.552	42,469.39	545.4075	1136.55
	std	2.07 × 10^−10^	70.52461	0.361508	0.002502	0.391792	137.0911	2634.042	228.0778	185.095	538.9384	15,004.15	133.6998	290.7565
	median	1	163.2553	4.738785	5.005	3.002399	818.8781	6193.637	565.8743	475.0553	736.7513	29,359.23	342.3155	859.0152
	rank	1	5	3	4	2	10	12	8	7	9	13	6	11
C19-F3	mean	1.252026	1.893453	2.128289	8.144879	1.464204	6.458567	6.143913	8.967309	1.907945	4.71987	4.00648	3.78097	5.784606
	best	1.055806	1.411199	1.410605	6.294517	1.173223	1.7408	3.067264	7.719486	1.208206	4.153685	2.763422	1.409135	3.599784
	worst	1.448438	2.377011	3.342791	9.221102	1.756207	9.724014	9.656947	10.71926	3.410963	5.801678	5.917239	7.694728	7.707829
	std	0.226445	0.555772	0.920676	1.305179	0.242174	3.405598	2.745714	1.499883	1.012835	0.749746	1.386399	3.016785	1.854326
	median	1.25193	1.8928	1.87988	8.531949	1.463694	7.184728	5.925721	8.715246	1.506306	4.462058	3.672631	3.010009	5.915405
	rank	1	3	5	12	2	11	10	13	4	8	7	6	9
C19-F4	mean	8.490857	17.43753	35.35101	74.01385	11.45606	53.14016	59.81028	25.57658	23.21939	35.45653	52.03346	18.92539	22.37215
	best	5.004053	13.94927	8.968627	55.9555	7.972665	41.50046	33.01698	22.91293	10.11592	32.7219	39.80328	10.94959	19.12583
	worst	9.98565	21.89463	57.77001	94.37194	14.94393	65.757	92.985	29.60309	38.84724	41.06531	60.75789	36.8552	27.50774
	std	2.373925	4.092957	20.27293	16.5466	3.503048	11.06681	26.38785	2.961748	13.72705	3.919584	9.132214	12.07601	3.595136
	median	9.486861	16.95311	37.33271	72.86398	11.45382	52.6516	56.61956	24.89516	21.95719	34.01946	53.78634	13.94839	21.42752
	rank	1	3	8	13	2	11	12	7	6	9	10	4	5
C19-F5	mean	1.040448	1.68947	1.202223	88.34774	1.035245	29.63456	2.041447	1.393396	1.59657	2.936446	1.177378	1.214415	1.727054
	best	1.021119	1.211649	1.109628	66.19828	1.008407	13.56856	1.695794	1.240528	1.348675	2.641299	1.155782	1.180765	1.552317
	worst	1.058037	2.265097	1.285169	109.1006	1.062662	62.12907	2.361459	1.756175	1.895775	3.143506	1.211316	1.254888	1.843677
	std	0.016864	0.456601	0.075539	17.55507	0.028366	22.90052	0.280528	0.246247	0.258672	0.224998	0.026365	0.038036	0.125116
	median	1.041318	1.640568	1.207046	89.04604	1.034956	21.42031	2.054267	1.28844	1.570914	2.980489	1.171207	1.211003	1.756112
	rank	2	8	4	13	1	12	10	6	7	11	3	5	9
C19-F6	mean	1.070443	2.903442	7.096602	9.950166	1.149309	7.862816	10.69014	3.026315	3.496531	4.579968	4.098299	3.646271	3.230283
	best	1.035309	1.803016	6.220347	9.465644	1.074	4.400796	9.384775	1.226604	1.337298	3.590748	1.072484	1.533005	2.493256
	worst	1.153231	3.607175	9.331887	10.76195	1.238936	10.55354	11.98097	4.368123	4.995392	5.235327	5.658356	6.246968	4.3958
	std	0.055523	0.795235	1.500173	0.609953	0.070639	2.602494	1.064884	1.374214	1.550139	0.724406	2.146191	1.984798	0.837016
	median	1.046616	3.101788	6.417086	9.786536	1.14215	8.248463	10.6974	3.255266	3.826717	4.746899	4.831178	3.402555	3.016039
	rank	1	3	10	12	2	11	13	4	6	9	8	7	5
C19-F7	mean	269.8869	491.5369	1083.674	1732.261	294.3786	1407.933	1142.598	1057.209	1178.452	1176.09	1661.754	1152.37	708.3514
	best	147.327	297.7043	804.5591	1578.7	156.2455	1023.022	703.3226	723.7527	1021.721	708.6485	1504.425	658.1489	491.0823
	worst	426.5517	759.5538	1547.829	1817.424	451.5611	1687.386	1794.3	1850.827	1377.603	1523.433	1797.54	1608.384	1094.838
	std	115.8062	207.68	353.8715	108.7197	123.085	279.7652	463.1718	533.431	167.8284	371.3769	133.0367	445.3167	282.551
	median	252.8345	454.4447	991.1533	1766.46	284.8538	1460.662	1036.385	827.1279	1157.242	1236.14	1672.527	1171.473	623.7426
	rank	1	3	6	13	2	11	7	5	10	9	12	8	4
C19-F8	mean	3.020737	3.56846	4.472057	4.821922	3.222844	4.153506	4.853111	4.062034	3.612028	4.304852	5.220798	4.611625	4.53273
	best	1.93757	3.019294	3.931216	4.542727	2.751649	3.564661	4.611607	3.565193	3.366284	4.059899	5.124828	4.373981	4.30402
	worst	3.832277	3.846303	4.899725	4.990379	3.684781	4.907803	5.087082	4.484265	4.04011	4.874945	5.373209	4.983297	4.673832
	std	0.81643	0.37258	0.457621	0.199997	0.491333	0.564	0.211666	0.435816	0.304162	0.383203	0.120079	0.26615	0.166595
	median	3.156551	3.704121	4.528644	4.877291	3.227473	4.07078	4.856877	4.09934	3.520859	4.142282	5.192578	4.544612	4.576533
	rank	1	3	8	11	2	6	12	5	4	7	13	10	9
C19-F9	mean	1.084898	1.176728	1.394348	3.107928	1.107921	1.351812	1.381376	1.17732	1.221403	1.325084	1.212712	1.19474	1.13892
	best	1.061955	1.123308	1.078336	2.403669	1.052251	1.199298	1.192892	1.151336	1.1062	1.253609	1.07988	1.090717	1.114774
	worst	1.118152	1.243717	1.605772	3.687815	1.177456	1.562261	1.652451	1.204837	1.357157	1.38638	1.303407	1.263166	1.185336
	std	0.026145	0.054588	0.225014	0.546669	0.054825	0.162107	0.217617	0.024619	0.113889	0.070005	0.098491	0.074806	0.031898
	median	1.079743	1.169944	1.446642	3.170114	1.100989	1.322845	1.34008	1.176555	1.211127	1.330173	1.23378	1.212539	1.127786
	rank	1	4	12	13	2	10	11	5	8	9	7	6	3
C19-F10	mean	18.13078	18.03091	21.05206	21.48397	21.0165	21.42982	21.22793	21.04321	21.49175	21.44677	22.69648	21.04806	21.28325
	best	6.932382	7.874518	20.99262	21.37514	21.003	21.37636	21.06805	21.00739	21.46041	21.39861	21.01899	20.99969	21.12875
	worst	22.06025	21.54158	21.19324	21.56887	21.021	21.55954	21.61559	21.06165	21.51818	21.52354	23.49109	21.15059	21.41332
	std	7.47515	6.778286	0.094957	0.080448	0.009009	0.08687	0.259764	0.024362	0.025405	0.054463	1.135734	0.069157	0.122919
	median	21.76525	21.35378	21.0112	21.49593	21.021	21.39169	21.11404	21.0519	21.49421	21.43248	23.13793	21.02097	21.29547
	rank	2	1	6	11	3	9	7	4	12	10	13	5	8
Sum rank		12	38	63	103	19	94	103	64	68	83	96	63	71
Mean rank		1.2	3.8	6.3	10.3	1.9	9.4	10.3	6.4	6.8	8.3	9.6	6.3	7.1
Total rank		1	3	4	11	2	9	11	5	6	8	10	4	7

**Table 7:** Wilcoxon rank sum test results.

Compared Algorithm	Objective Function Type				
	CEC 2017				CEC 2019
	m=10	m=30	m=50	m=100	
GAO vs. WSO	5.4 × 10^−15^	3.18 × 10^−21^	2.07 × 10^−21^	2.29 × 10^−21^	5.91 × 10^−6^
GAO vs. AVOA	2.8 × 10^−20^	6.88 × 10^−21^	1.97 × 10^−21^	2.41 × 10^−21^	4.49 × 10^−6^
GAO vs. RSA	3.6 × 10^−21^	1.97 × 10^−21^	1.97 × 10^−21^	1.97 × 10^−21^	2.79 × 10^−7^
GAO vs. MPA	2.43 × 10^−5^	4.35 × 10^−10^	1.49 × 10^−11^	3.34 × 10^−15^	0.006264
GAO vs. TSA	7.23 × 10^−21^	1.97 × 10^−21^	1.97 × 10^−21^	1.97 × 10^−21^	1.37 × 10^−7^
GAO vs. WOA	9.04 × 10^−21^	1.97 × 10^−21^	1.97 × 10^−21^	1.97 × 10^−21^	1.84 × 10^−7^
GAO vs. MVO	8.04 × 10^−17^	5.13 × 10^−19^	5.68 × 10^−20^	6.41 × 10^−20^	4.64 × 10^−7^
GAO vs. GWO	1.44 × 10^−18^	9.98 × 10^−21^	5.64 × 10^−21^	9.5 × 10^−21^	1.49 × 10^−6^
GAO vs. TLBO	7.06 × 10^−20^	1.97 × 10^−21^	1.97 × 10^−21^	1.97 × 10^−21^	2.28 × 10^−7^
GAO vs. GSA	3.02 × 10^−20^	2.87 × 10^−21^	1.97 × 10^−21^	1.97 × 10^−21^	3.57 × 10^−8^
GAO vs. PSO	6.25 × 10^−20^	9.98 × 10^−21^	1.72 × 10^−20^	2.24 × 10^−21^	7.06 × 10^−7^
GAO vs. GA	3.09 × 10^−20^	1.97 × 10^−21^	2.02 × 10^−21^	1.97 × 10^−21^	1.59 × 10^−7^

**Table 8:** Optimization results of the CEC 2011 test suite.

		GAO	WSO	AVOA	RSA	MPA	TSA	WOA	MVO	GWO	TLBO	GSA	PSO	GA
C11-F1	mean	3.300478	15.02557	25.74562	26.00412	3.521917	20.93273	24.34703	20.30298	13.87947	19.79529	25.67295	19.27164	25.88474
	best	4.16 × 10^−10^	11.75673	24.83901	25.15683	4.53 × 10^−10^	12.96597	22.0416	16.91438	1.775842	17.00127	23.71442	8.416087	24.27333
	worst	13.20191	19.29177	26.94225	27.78769	14.08767	25.02322	25.15126	24.93986	21.48584	22.33001	27.19836	24.58904	27.26328
	std	6.607557	3.132729	1.052163	1.210689	7.050878	5.524893	1.5387	3.858004	8.592199	2.515545	1.728391	7.344787	1.456137
	median	2.19 × 10^−9^	14.52688	25.60061	25.53597	2.31 × 10^−9^	22.87087	25.09763	19.67884	16.12811	19.92494	25.88952	22.04072	26.00117
	rank	1	4	11	13	2	8	9	7	3	6	10	5	12
C11-F2	mean	−26.4809	−22.3944	−11.6301	−8.06028	−25.3005	−8.61772	−13.6102	−8.31426	−23.7019	−9.22187	−7.19675	−23.0507	−11.4007
	best	−27.2293	−22.8813	−14.0599	−8.64276	−26.6172	−13.6641	−20.5858	−10.1138	−25.4598	−9.68401	−8.44895	−25.5945	−14.0856
	worst	−25.364	−21.4054	−9.06418	−7.42364	−24.2799	−4.97971	−8.78687	−6.02627	−21.5016	−9.00778	−6.49843	−20.5637	−9.44663
	std	0.825599	0.675642	2.045502	0.551116	1.068355	3.956082	4.985352	2.113823	1.696846	0.31722	0.911664	2.056796	2.156855
	median	−26.6652	−22.6454	−11.6981	−8.08736	−25.1525	−7.91351	−12.534	−8.55848	−23.9231	−9.09784	−6.9198	−23.0223	−11.0353
	rank	1	5	7	12	2	10	6	11	3	9	13	4	8
C11-F4	mean	−35.3025	−29.4024	−17.5227	−16.7272	−32.5006	−27.7957	−23.1492	−27.1284	−32.0504	−13.2769	−27.5051	−7.4195	−4.2567
	best	−35.4432	−33.8574	−19.4025	−19.341	−34.2508	−30.6982	−26.4941	−31.727	−34.1547	−20.0052	−34.1725	−10.5338	−7.32587
	worst	−35.1685	−27.2841	−16.1919	−13.8236	−31.5536	−25.9792	−17.1297	−24.2157	−30.4483	−5.71506	−19.9298	−2.61069	0
	std	0.153785	3.07155	1.378688	2.851785	1.199952	2.140053	4.438601	3.220723	1.563641	6.08285	6.544209	3.393814	3.200788
	median	−35.2992	−28.234	−17.2482	−16.8722	−32.099	−27.2526	−24.4866	−26.2855	−31.7992	−13.6936	−27.9591	−8.26678	−4.85046
	rank	1	4	9	10	2	5	8	7	3	11	6	12	13
C11-F5	mean	−28.2978	−19.3848	−10.0921	−10.5136	−27.3199	−12.1459	−18.3796	−5.08497	−22.3633	0	−16.3851	0	−0.8672
	best	−29.1661	−23.0059	−14.7254	−11.5854	−28.0821	−21.209	−23.0034	−20.3399	−26.5965	0	−23.0059	0	−3.46879
	worst	−27.4293	−16.8457	−7.09818	−9.51564	−26.5001	0	−15.0879	0	−19.5117	0	−8.64304	0	0
	std	1.003611	2.649821	3.386136	1.15452	0.874335	9.192985	3.399914	10.18012	3.19482	0	5.905717	0	1.736128
	median	−28.2979	−18.8438	−9.27249	−10.4768	−27.3487	−13.6873	−17.7135	0	−21.6726	0	−16.9457	0	0
	rank	1	4	9	8	2	7	5	10	3	12	6	12	11
C11-F6	mean	0.785253	1.14754	2.024755	2.155071	0.867687	1.452179	2.188145	1.068278	1.208421	1.940935	1.044281	1.257057	2.212929
	best	0.665035	0.998087	1.6614	1.83069	0.765584	1.177085	2.075871	0.954839	0.820258	1.889914	0.649331	0.85164	1.95787
	worst	0.875085	1.258533	2.187947	2.345592	1.064378	1.650897	2.297034	1.214887	1.950702	1.998965	1.272956	1.715245	2.385961
	std	0.088232	0.129965	0.247268	0.227312	0.135582	0.206732	0.091469	0.129917	0.523394	0.045935	0.272997	0.354366	0.192515
	median	0.800446	1.16677	2.124836	2.222001	0.820394	1.490368	2.189838	1.051694	1.031362	1.937431	1.127418	1.230672	2.253942
	rank	1	5	10	11	2	8	12	4	6	9	3	7	13
C11-F7	mean	220	220	276.5	349.25	220	425.5043	291.5	224.5	229	227	276.3886	826.2645	239.25
	best	220	220	230	299	220	220	251	220	220	220	231	251	220
	worst	220	220	323	404	220	1042.017	351	238	256	238	350.5545	2184.43	259
	std	0	0	40.06085	43.11181	0	411.4195	45.36272	9.009	18.018	8.726516	55.21181	913.4908	15.96116
	median	220	220	276.5	347	220	220	282	220	220	225	262	434.8141	239
	rank	1	1	7	9	1	10	8	2	4	3	6	11	5
C11-F8	mean	9852.127	735,450.4	1,029,600	1,301,689	9549.131	69,153.55	548,774.3	93,478.54	28,444.15	625,190.3	955,208.4	981,885.4	2,206,877
	best	6182.4	547,467.2	914,559	848,186.4	6045.388	47,218.02	446,320.4	46,944.39	21,243.75	506,258.2	710,857.7	561,536	2,183,812
	worst	12,039.41	873,535.9	1,112,084	1,528,229	11,684.98	78,837.01	612,232.3	166,066.1	34,209.17	713,782.7	1,193,111	1,351,642	2,234,596
	std	2542.993	147,235.4	86,895.62	308,607.9	2436.763	14,807.58	71,801.99	51,002.17	5811.795	97,974.59	198,631	328,589.5	25,018.57
	median	10,593.35	760,399.2	1,045,878	1,415,170	10,233.08	75,279.58	568,272.3	80,451.8	29,161.84	640,360.1	958,432.4	1,007,182	2,204,550
	rank	2	8	11	12	1	4	6	5	3	7	9	10	13
C11-F9	mean	−22.2297	−16.4886	−8.89083	−10.1972	−21.5337	−11.918	−10.1325	−17.3129	−16.9511	−10.2886	−11.6632	−10.3517	−9.99235
	best	−22.2933	−18.7915	−9.07622	−10.5958	−21.6259	−15.7644	−10.7556	−21.5793	−21.4972	−10.3194	−11.8653	−10.3775	−10.0444
	worst	−22.1512	−14.5174	−8.70287	−9.81793	−21.3959	−9.99611	−9.63545	−10.3361	−12.1207	−10.243	−11.1938	−10.2988	−9.94759
	std	0.072979	2.101764	0.195165	0.318176	0.109809	2.63253	0.583689	5.242781	5.152578	0.034325	0.315282	0.036848	0.041836
	median	−22.2372	−16.3228	−8.89211	−10.1875	−21.5565	−10.9557	−10.0695	−18.668	−17.0932	−10.296	−11.7969	−10.3653	−9.9887
	rank	1	5	13	10	2	6	11	3	4	9	7	8	12
C11-F10	mean	275,953.6	295,554.2	1,728,003	10,733,179	658,101.1	8,232,813	1,532,582	1,074,459	4,504,623	5,689,705	1,581,079	5,704,387	6,706,720
	best	111,906.1	119,989.6	1,671,593	10,440,242	457,716.5	5,796,617	1,337,713	821,072.7	4,262,675	5,689,705	1,296,552	5,689,705	6,664,292
	worst	435,202.3	462,364	1,774,439	10,907,934	876,974.8	11,926,409	1,706,194	1,263,682	4,637,288	5,689,705	1,730,846	5,723,987	6,745,470
	std	133,433.5	141,153.2	43,998.11	203,866.3	219,334.8	2,613,703	171,410.9	198,494.1	173,309.1	0	193,907.3	17,440.09	40,315.03
	median	278,353	299,931.5	1,732,991	10,792,271	648,856.6	7,604,113	1,543,210	1,106,542	4,559,265	5,689,705	1,648,460	5,701,929	6,708,560
	rank	1	2	7	13	3	12	5	4	8	9	6	10	11
C11-F11	mean	1,255,580	3,917,084	7,200,862	15,954,845	1,339,465	6,290,493	6,632,909	1,360,726	1,564,264	15,605,750	6,891,201	2,353,372	15,830,924
	best	1,163,262	3,701,366	6,994,873	14,807,307	1,251,074	5,759,789	6,399,676	1,265,380	1,294,515	14,467,675	6,772,346	2,135,592	15,403,057
	worst	1,319,053	4,098,419	7,301,082	16,959,124	1,419,141	7,038,850	6,789,678	1,434,252	1,817,236	16,563,952	6,988,061	2,733,600	16,293,068
	std	65,830.74	176,751.4	139,435.4	883,563.4	69,069.69	556,954.8	167,909.8	71,081.87	231,779.7	890,323.8	93,690.64	272,693.2	385,833
	median	1,270,002	3,934,275	7,253,747	16,026,475	1,343,823	6,181,665	6,671,142	1,371,635	1,572,653	15,695,687	6,902,198	2,272,149	15,813,785
	rank	1	6	10	13	2	7	8	3	4	11	9	5	12
C11-F12	mean	15,444.21	15,689.16	15,454.67	16,506.66	16,407.44	15,508.05	15,512.23	15,485.82	15,505.75	16,060.09	131,124.1	15,519.94	18,237.52
	best	15,444.19	15,444.23	15,451.9	15,996.18	15,444.19	15,481.63	15,477.65	15,455.13	15,478.25	15,557.26	80,466.2	15,498.93	15,484.07
	worst	15,444.23	16,387.09	15,457.72	17,774.94	16,970.37	15,533.33	15,548.93	15,509.95	15,552.24	16,984.35	234,290	15,531.07	25,305.01
	std	0.020683	465.9733	2.453554	853.3277	700.8544	24.77537	30.85129	22.75544	32.19287	640.2204	70,818.17	14.34255	4739.424
	median	15,444.21	15,462.66	15,454.53	16,127.77	16,607.61	15,508.62	15,511.18	15,489.09	15,496.25	15,849.38	104,870.1	15,524.88	16,080.5
	rank	1	8	2	11	10	5	6	3	4	9	13	7	12
C11-F13	mean	18,766.8	18,401.87	18,913.78	276,941.8	18,236.95	19,424.61	19,313	19,268.63	19,403.55	194,506.5	19,156.17	19,347.18	19,047.76
	best	18,634.88	18,212.02	18,679.04	202,857.5	18,194.76	19,121.09	18,855.02	18,783.36	19,265.81	61,049.2	18,921.5	19,111.92	18,922.68
	worst	18,882.86	18,524.11	19,086.98	400,961	18,297.22	19,971.19	19,580.38	19,581.93	19,546.94	387,895.1	19,457.67	19,602.18	19,231.56
	std	102.2456	134.2627	181.9858	88,823.39	47.91151	374.7453	317.2627	341.1623	134.9524	145,574.9	223.2481	265.7884	134.1612
	median	18,774.74	18,435.69	18,944.54	251,974.4	18,227.92	19,303.08	19,408.29	19,354.62	19,400.72	164,540.9	19,122.76	19,337.31	19,018.39
	rank	3	2	4	13	1	11	8	7	10	12	6	9	5
C11-F14	mean	32,845.08	33,006.71	239,954.4	2,343,498	35,130.85	35,121.25	106,277.4	33,151.84	33,126.78	11,213,596	345,205.2	33,338.43	10,339,440
	best	32,809.43	32,844.61	61,174.85	975,713.9	34,595.97	33,144.97	33,167.07	32,986.32	32,995.68	3,908,323	256,209.7	33,323.21	3,973,087
	worst	32,880.63	33,126.57	439,827.3	6,129,448	35,975.27	40,825.27	215,083.4	33,336.37	33,260.65	17,145,622	392,625.8	33,355.63	18,906,172
	std	32.99729	135.0197	203,624.7	2,530,421	594.1606	3806.85	89,156.14	145.1283	133.9616	6,524,456	60,919.36	14.07213	6,314,495
	median	32,845.14	33,027.82	229,407.7	1,134,415	34,976.08	33,257.39	88,429.47	33,142.34	33,125.38	11,900,220	365,992.7	33,337.45	9,239,251
	rank	1	2	9	11	7	6	8	4	3	13	10	5	12
C11-F15	mean	133,287.9	140,692.8	140,076.2	2,362,861	141,598.2	149,095.6	146,593	141,880.2	141,577	98,977,989	21,150,022	94,639,175	85,867,595
	best	131,118.5	134,258.9	137,666.4	551,007.8	134,966.6	146,561.1	141,445.7	137,935.6	136,714	88,655,574	2,767,679	74,104,169	33,265,483
	worst	134,522.7	151,586.1	142,366.4	5,916,825	145,841.3	152,103.6	156,853.3	147,252.9	149,667.1	1.19 × 10^8^	42,558,036	1.07 × 10^8^	1.09 × 10^8^
	std	1526.05	7718.994	2597.529	2,415,484	4710.937	2385.079	7075.949	4394.903	6061.095	13,890,967	16,645,999	14,574,652	35,688,282
	median	133,755.2	138,463.1	140,136	1,491,805	142,792.5	148,858.9	144,036.6	141,166.2	139,963.5	94,111,524	19,637,186	98,813,738	1.01 × 10^8^
	rank	1	3	2	9	5	8	7	6	4	13	10	12	11
C11-F16	mean	1,941,587	4,117,826	6.39 × 10^9^	1.91 × 10^10^	2,040,837	9.08 × 10^8^	1.14 × 10^10^	2,613,192	2,499,625	2.23 × 10^10^	1.45 × 10^10^	1.94 × 10^10^	2.1 × 10^10^
	best	1,931,795	1,945,829	5.81 × 10^9^	1.37 × 10^10^	1,966,761	1.5 × 10^8^	9.92 × 10^9^	2,226,241	2,159,827	1.83 × 10^10^	1.24 × 10^10^	1.55 × 10^10^	1.76 × 10^10^
	worst	1,954,752	9,745,854	7 × 10^9^	2.33 × 10^10^	2,071,974	1.41 × 10^9^	1.25 × 10^10^	3,051,146	3,268,005	2.66 × 10^10^	1.89 × 10^10^	2.36 × 10^10^	2.53 × 10^10^
	std	10,357.32	3,769,921	5.24 × 10^8^	4.13 × 10^9^	49,645.15	5.57 × 10^8^	1.16 × 10^9^	377,328.4	520,697.6	3.47 × 10^9^	3.04 × 10^9^	3.31 × 10^9^	3.41 × 10^9^
	median	1,939,901	2,389,810	6.38 × 10^9^	1.96 × 10^10^	2,062,306	1.03 × 10^9^	1.16 × 10^10^	2,587,691	2,285,334	2.21 × 10^10^	1.32 × 10^10^	1.93 × 10^10^	2.05 × 10^10^
	rank	1	5	7	10	2	6	8	4	3	13	9	11	12
C11-F17	mean	971,895.4	1,616,414	16,449,493	1.45 × 10^8^	942,207	2,194,127	8,381,389	1,024,624	1,131,755	34,761,039	9,159,466	1.79 × 10^8^	1.25 × 10^8^
	best	966,588.8	1,094,971	9,228,474	1 × 10^8^	939,995.7	1,628,158	2,842,721	958,818.7	977,032.3	27,241,740	942,142.4	1.57 × 10^8^	1.22 × 10^8^
	worst	977,835.8	2,285,712	29,396,167	1.66 × 10^8^	947,316	2,469,335	17,189,839	1,193,542	1,328,731	37,388,284	20,419,966	2.05 × 10^8^	1.27 × 10^8^
	std	6043.012	494,783.1	9,399,624	30,732,120	3441.709	385,943.9	6,235,833	113,169.6	170,025	5,019,190	8,622,080	23,693,281	1,843,552
	median	971,578.6	1,542,486	13,586,665	1.57 × 10^8^	940,758.1	2,339,508	6,746,499	973,067.3	1,110,628	37,207,066	7,637,878	1.78 × 10^8^	1.25 × 10^8^
	rank	2	5	9	12	1	6	7	3	4	10	8	13	11
C11-F18	mean	1,015,441	2,590,116	16,582,334	1.42 × 10^8^	988,564.9	2,966,643	7,039,845	1,550,619	1,435,166	35,274,115	17,309,844	1.72 × 10^8^	1.25 × 10^8^
	best	970,440.6	1,954,981	14,730,898	1.23 × 10^8^	943,556.8	2,458,931	4,995,010	1,347,378	1,246,367	28,101,008	15,122,456	1.6 × 10^8^	1.21 × 10^8^
	worst	1,082,763	3,891,235	20,548,870	1.79 × 10^8^	1,060,958	3,238,732	9,466,270	1,926,756	1,590,758	46,848,216	18,652,552	1.84 × 10^8^	1.28 × 10^8^
	std	52,663	880,283.7	2,680,868	26,129,675	54,245.33	363,495.4	2,012,868	259,593.2	174,182.5	8,801,161	1,578,438	12,279,513	3,177,603
	median	1,004,281	2,257,125	15,524,785	1.34 × 10^8^	974,872.4	3,084,455	6,849,050	1,464,171	1,451,771	33,073,619	17,732,185	1.72 × 10^8^	1.26 × 10^8^
	rank	2	5	8	12	1	6	7	4	3	10	9	13	11
C11-F19	mean	974,847.4	3,058,450	14,619,890	1.54 × 10^8^	981,440.7	2,527,801	7,589,122	965,971	1,036,557	30,666,064	12,400,485	1.92 × 10^8^	1.29 × 10^8^
	best	944,022.2	1,019,342	12,702,812	1.34 × 10^8^	945,830.8	2,410,961	2,746,274	951,663.6	960,231.2	28,071,917	11,125,050	1.72 × 10^8^	1.26 × 10^8^
	worst	1,014,915	8,406,301	16,683,672	1.83 × 10^8^	1,019,498	2,730,807	13,705,947	1,001,277	1,185,908	36,835,950	15,782,650	2.21 × 10^8^	1.31 × 10^8^
	std	36,191.37	3,584,352	1,653,942	20,589,549	39,571.94	152,159.2	4,553,151	23,629.47	101,531.9	4,176,821	2,261,768	21,032,934	2,385,169
	median	970,226.3	1,404,078	14,546,539	1.49 × 10^8^	980,217.1	2,484,719	6,952,133	955,471.6	1,000,045	28,878,195	11,347,120	1.88 × 10^8^	1.3 × 10^8^
	rank	2	6	9	12	3	5	7	1	4	10	8	13	11
C11-F20	mean	10.49163	18.43484	37.38995	91.01355	11.21275	29.05794	40.96432	29.92222	25.24142	129.9098	44.83753	121	95.16216
	best	8.851506	17.10456	35.08192	66.45898	9.358565	19.49597	28.1708	22.61783	24.07106	106.0516	31.58387	109.7915	78.9184
	worst	13.71088	20.77307	38.49468	115.651	14.72046	37.54154	56.17231	36.91662	26.50252	168.6126	53.55251	130.3309	126.8521
	std	2.187018	1.638565	1.604061	21.75983	2.3932	7.98459	14.64249	5.883188	0.997189	26.96817	9.367526	10.30455	21.87485
	median	9.702059	17.93087	37.9916	90.97211	10.386	29.59713	39.75708	30.07722	25.19605	122.4875	47.10687	121.9389	87.43907
	rank	1	3	7	10	2	5	8	6	4	13	9	12	11
C11-F21	mean	13.73264	27.48089	47.41507	74.30445	14.63561	34.45979	38.14793	32.33235	23.10522	99.56062	59.11908	114.8943	114.5445
	best	12.11314	25.26953	41.00099	52.03457	12.85559	30.74852	36.12422	26.44499	21.55421	67.47136	47.68592	110.8849	84.39572
	worst	17.85657	32.64845	56.32061	85.38791	18.951	36.50066	41.20724	38.10353	25.6474	131.7346	70.84651	119.2162	147.2729
	std	2.759916	3.47892	7.092501	15.12056	2.890697	2.552249	2.1656	6.458469	1.934922	28.15673	9.498156	3.70323	27.02112
	median	12.48042	26.00279	46.16933	79.89766	13.36792	35.295	37.63014	32.39043	22.60963	99.51824	58.97195	114.7381	113.2546
	rank	1	4	8	10	2	6	7	5	3	11	9	13	12
C11-F22	mean	3.300478	15.02557	25.74562	26.00412	3.521917	20.93273	24.34703	20.30298	13.87947	19.79529	25.67295	19.27164	25.88474
	best	4.16 × 10^−10^	11.75673	24.83901	25.15683	4.53 × 10^−10^	12.96597	22.0416	16.91438	1.775842	17.00127	23.71442	8.416087	24.27333
	worst	13.20191	19.29177	26.94225	27.78769	14.08767	25.02322	25.15126	24.93986	21.48584	22.33001	27.19836	24.58904	27.26328
	std	6.607557	3.132729	1.052163	1.210689	7.050878	5.524893	1.5387	3.858004	8.592199	2.515545	1.728391	7.344787	1.456137
	median	2.19 × 10^−9^	14.52688	25.60061	25.53597	2.31 × 10^−9^	22.87087	25.09763	19.67884	16.12811	19.92494	25.88952	22.04072	26.00117
	rank	1	4	11	13	2	8	9	7	3	6	10	5	12
Sum rank		27	88	160	222	54	142	152	100	84	201	167	193	219
Mean rank		1.285714	4.190476	7.619048	10.57143	2.571429	6.761905	7.238095	4.761905	4	9.571429	7.952381	9.190476	10.42857
Total rank		1	4	8	13	2	6	7	5	3	11	9	10	12
Wilcoxon: *p*-value			4.8 × 10^−12^	8.49 × 10^−15^	1.71 × 10^−15^	0.001914	5.36 × 10^−15^	5.76 × 10^−15^	1.75 × 10^−11^	2.11 × 10^−12^	3.66 × 10^−15^	8.8 × 10^−15^	1.71 × 10^−15^	2.5 × 10^−15^

## Data Availability

Not applicable.
